# Robust biexciton preparation for entangled photon-pair generation by single-shot Floquet driving

**DOI:** 10.1038/s41467-026-77221-9

**Published:** 2026-09-12

**Authors:** Jun-Yong Yan, Paul C. A. Hagen, Hang-Yu Ge, Fang-Yuan Li, Hans-Georg Babin, Wei E. I. Sha, Bo Wang, Zhi-hua Gan, Andreas D. Wieck, Arne Ludwig, Chao-Yuan Jin, Vollrath M. Axt, Da-Wei Wang, Moritz Cygorek, Feng Liu

**Affiliations:** 1 State Key Laboratory of Extreme Photonics and Instrumentation, College of Information Science and Electronic Engineering, Zhejiang University, Hangzhou, China; 2 Theoretische Physik III, Universität Bayreuth, Bayreuth, Germany; 3 Experimental Physics VI, Ruhr University Bochum, Bochum, Germany; 4 Cryogenic Center, Hangzhou City University, Hangzhou, China; 5 Zhejiang Key Laboratory of Refrigeration and Cryogenic Technology, Zhejiang University, Hangzhou, China; 6 ZJU-Hangzhou Global Scientific and Technological Innovation Center, Zhejiang University, Hangzhou, China; 7 Zhejiang Key Laboratory of Micro-Nano Quantum Chips and Quantum Control, School of Physics, Zhejiang University, Hangzhou, China; 8 Condensed Matter Theory, Department of Physics, TU Dortmund, Dortmund, Germany; 9 Present Address: Department of Electrical and Computer Engineering, National University of Singapore, Singapore, Singapore

**Keywords:** Single photons and quantum effects, Quantum dots, Quantum optics

## Abstract

Quantum emitters driven by resonant two-photon excitation are a leading source for deterministically generated entangled photon pairs, essential for scalable photonic quantum technologies. However, conventional excitation schemes pose various constraints, limiting their scalability and practicability. Here, we demonstrate how biexciton preparation schemes with significantly improved robustness and reduced experimental demands can be identified using a novel design principle: *ultrafast single-shot Floquet driving*. This is achieved by employing two strongly and symmetrically detuned pulses, whose superposition generates a stroboscopic Hamiltonian that enables direct coupling between ground and biexciton states. Moreover, a pulse delay serves as a tuning knob, introducing an effective magnetic field making biexciton preparation particularly robust. Experimentally, we achieve a biexciton occupation exceeding 96% and preserve photon-pair entanglement with a fidelity of 93.4%. Our work opens avenues for the design of quantum control protocols with superior performance across diverse quantum emitters and qubit platforms.

## Abstract (web summary)

Deterministic generation of entangled photon pairs from quantum dots requires robust preparation of the biexciton state. Here, the authors demonstrate single-shot Floquet driving using symmetrically detuned optical pulses to prepare the biexciton with high efficiency and reduced sensitivity to pulse-area fluctuations, enabling the generation of polarization-entangled photon pairs with high fidelity.

## Introduction

Quantum entanglement, one of the most intriguing phenomena in the quantum world, lies at the heart of quantum information science. A deterministic source of entangled photons is a fundamental building block for modern quantum photonics applications, such as quantum communications^1,2^, optical quantum computing^3,4^, quantum imaging and sensing^5,6^. A semiconductor quantum dot (QD), often referred to as an artificial atom due to its three-dimensional electronic confinement and discrete energy levels, is a well-established source of highly entangled photons^7–9^. The on-demand high-fidelity preparation of the biexciton state is the starting point for the radiative cascade decay process that generates entangled photon pairs. Moreover, cascade-emitted photons have been shown to induce simultaneous excitation of two independent quantum emitters^10^, enabling the synthesis of effective multi-qubit interactions and coherent generation of entangled states^11^.

**Fig. 1:**
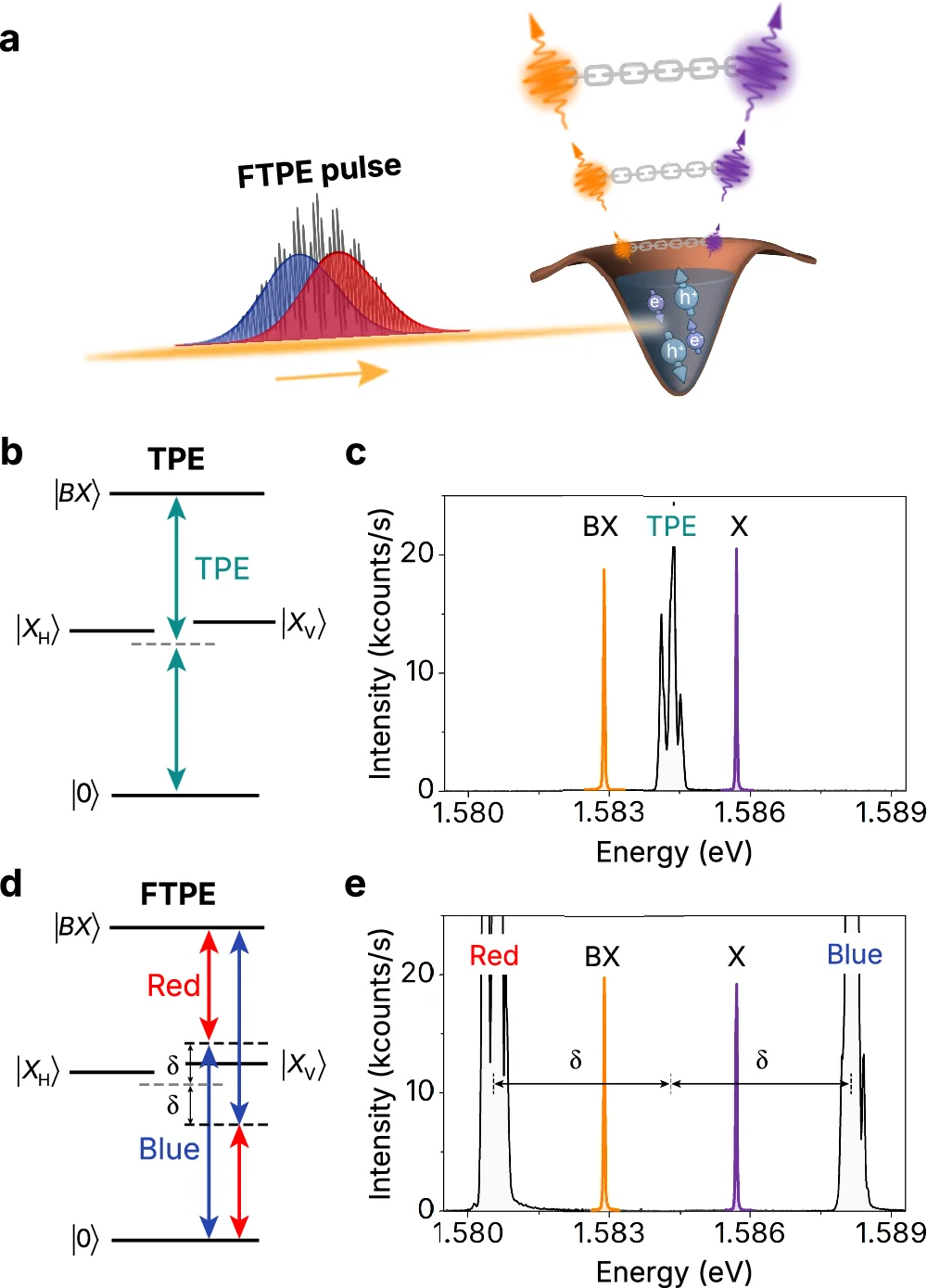
TPE and FTPE schemes. **a** Biexciton state prepared by FTPE emits pairs of entangled photons. **b** Schematic of the TPE process. **c** Laser excitation spectrum under TPE, where the energy of the TPE pulse is set at the average energy of the *BX* and *X* photons. **d** Schematic of FTPE. **e** Laser excitation spectrum under FTPE, where the blue and red pulses are detuned symmetrically to meet the two-photon resonance condition.

A degenerate resonant two-photon excitation (TPE) scheme, where two photons with identical frequencies are absorbed simultaneously, is commonly used to coherently prepare the biexciton state with minimal dephasing and time jitter^12^ (see Fig. 1b). This excitation scheme has shown significant potential in generating polarization-entangled photon pairs^13–15^, time-bin entangled photons^16,17^ and indistinguishable single photons^18–20^. However, conventional TPE relies on Rabi oscillations of biexciton populations, making its excitation efficiency highly sensitive to variations in the excitation laser power and the dipole moment of the emitters. Furthermore, tuning the laser to the average frequency of the biexciton (*BX*) and exciton (*X*) photons is unfavorable for QDs with small or negative *BX* binding energy. In the former case^21,22^, the tiny detuning between the laser and the entangled photon pairs makes it particularly difficult to perform effective laser filtering. In the latter case, the TPE process competes with phonon-assisted excitation of *X*, reducing the preparation efficiency of *BX*^23^. These limitations hinder the performance and scalability of on-demand entangled photon sources. Although several alternative approaches based on a single excitation pulse, such as rapid adiabatic passage^24–26^ and LA-phonon-assisted excitation^27–29^, have been explored, the simultaneous realization of robust state preparation and the generation of high-fidelity entangled photon pairs has not yet been achieved. Moreover, while multi-colour excitation schemes such as the swing-up of quantum emitter (SUPER)^30–32^ for biexciton preparation have been proposed, their experimental implementation for entangled-photon generation has not yet been demonstrated.

Floquet engineering, which employs periodic driving to achieve desired quantum dynamics, offers a fundamentally different route for overcoming the limitations of existing excitation schemes. According to Floquet’s theorem^33^, when a quantum system is periodically driven with a period *T*, the time evolution operator evaluated at stroboscopic times (integer multiples of *T*) takes the same form as for a time-independent Hamiltonian (see Methods Section). This stroboscopic Hamiltonian governs the behaviour of the quantum system on a coarse-grained time scale ≳ *T*. Moreover, if the driving oscillates periodically within a slowly varying envelope, the system approximately follows the instantaneous stroboscopic Hamiltonian, which is then time-dependent on the scale of the envelope^34^. The ability to control and tune the properties of quantum systems by Floquet engineering has enabled the realization of photonic topological insulators^35^ and synthetic gauge fields in photonic systems^36^ and in ultracold atomic systems^37^.

Existing Floquet engineering approaches typically utilize modulated continuous-wave laser fields or repetitive laser pulse trains. In the context of on-demand semiconductor quantum light sources, Floquet engineering has focused mainly on engineering excitonic wave functions by periodic microwave driving^38^. Despite these advances, all-optical Floquet engineering with *single-cycle pulses* to manipulate the optical excitation dynamics remains unexplored.

In this article, we propose and demonstrate a scheme for robust on-demand biexciton preparation and entangled photon generation enabled by all-optical single-shot Floquet driving [Fig. 1a]. Our key observation is that one can realize a time-dependent stroboscopic Hamiltonian that directly drives the ground-to-biexciton transition of a semiconductor quantum dot simply by dichromatic excitation with laser pulses symmetrically detuned from the degenerate two-photon resonance by a detuning *δ* [Fig. 1d, e]. Further requirements are that the detuning is large enough so that oscillations with frequency *δ* are fast compared to changes in the pulse envelope and that the pulses overlap temporally. Control of the phase relation between the pulses is not required (see Supplementary Sect. XI). These requirements are well achievable within current state-of-the-art experimental techniques for two-colour excitation^39–42^. Moreover, the dichromatic pulses can be detuned far from the *X* and *BX* photons, relaxing the requirement for laser filtering, especially for QDs with small or negative *BX* binding energy. Hence, our proposed excitation scheme, which we refer to as *Floquet-engineered Two-Photon Excitation* (FTPE), can be readily implemented for a wide range of QDs. In our experiments, we demonstrate a *BX* preparation efficiency of 96.6% and a polarization-entanglement fidelity of 93.4%, proving its reliability.

## Results

### Theory on Floquet-engineered two-photon excitation

Figure 1a illustrates the proposed FTPE scheme applied to a semiconductor QD. A pair of temporally partially overlapping pulses (depicted in blue and red) generates a periodic driving field (indicated by gray lines). This periodic driving deterministically and reliably creates a biexciton in the QD, which subsequently emits entangled photon pairs via the cascade decay process.

To model the FTPE process, we consider a ladder system composed of a ground state $\vert 0 \rangle$, a vertically (V) polarized exciton $\left\vert {X}_{{{\rm{V}}}}\right. \rangle$, and a biexciton state $\left\vert BX\right. \rangle$. We assume that both driving lasers are vertically polarized such that the horizontally (H) polarized exciton is not involved in the excitation process. The energy of $\left\vert BX\right. \rangle$ is *E*_*BX*_ = 2*E*_*X*_ − *E*_*b*_, where *E*_*X*_ is the energy of the *X* state and *E*_*b*_ is the biexciton binding energy. Figure 1b shows the schematic of conventional TPE, where the laser is tuned to be strictly in resonance with the virtual state of the $\left\vert 0\right. \rangle$ to $\left\vert BX\right. \rangle$ degenerate two-photon transition and Rabi oscillations can be observed^12^. A representative TPE laser excitation spectrum is presented in Fig. 1c. The laser frequency is tuned to the midpoint between the *BX* and *X* emission lines. In contrast, as shown in Fig. 1d, the two colour lasers used in FTPE are detuned to lie outside the *BX* and *X* lines. Thus, a large range of laser detunings is allowed as long as the two-photon resonance is maintained^43^, i.e., *E*_blue_+*E*_red_=*E*_*BX*_. The higher- and lower-energy lasers are labeled as blue and red pulses, respectively. Since the $\left\vert BX\right. \rangle$ to $\left\vert {X}_{V}\right. \rangle$ and $\left\vert {X}_{V}\right. \rangle$ to $\left\vert 0\right. \rangle$ transitions have the same optical polarization selection rule, each laser pulse simultaneously couples to both transitions [Fig. 1d]. Figure 1e shows a representative laser excitation spectrum under FTPE. The detuning of either laser from conventional TPE resonance is labeled as *δ*. Some ripples in the excitation pulse spectra are attributed to the polarization optics used in the setup. In the frame rotating with the frequency of resonant TPE (*E*_*BX*_/(2*ℏ*)), the Hamiltonian in the basis {*BX*, *X*_V_, 0} is given by

1 $$H/\hslash=\left(\begin{array}{lll}0&{\Omega }^{*}(t)/2&0\\ \Omega (t)/2&{E}_{b}/(2\hslash )&{\Omega }^{*}(t)/2\\ 0&\Omega (t)/2&0\end{array}\right),$$

where the light-matter interaction term contains two equally strong laser pulses *Ω*(*t*) = *f*(*t* − *τ*/2)*e*^−*iδ*(*t*−*τ*/2)^ + *f*(*t* + *τ*/2)*e*^*iδ*(*t*+*τ*/2)^, which can be delayed relative to each other by a time *τ*. The individual pulse envelopes are Gaussian $f(t)=\frac{\varTheta }{\sqrt{2\pi }s}{e}^{-{t}^{2}/2{s}^{2}}$ with single-photon transition pulse area *Θ* and Gaussian standard deviation $s=\frac{{\tau }_{0}}{2\sqrt{2\,{\mbox{ln}}\,2}}$ related to the FWHM duration of the electric field *τ*_0_ = 12.49 ps. The pulse area is defined as ${ \varTheta}=\left\vert \frac{\mu }{\hslash }\int_{-\infty }^{+\infty }{{{\mathcal{E}}}}_{0}(t){{\rm{d}}}t\right\vert$, where *μ* is the transition dipole moment for each single-photon transition, and ${{{\mathcal{E}}}}_{0}(t)$ is the electric-field amplitude of either the blue- or red-detuned pulse. The full quantum dynamics of the driven QD including phonon effects^44,45^ is simulated using the numerically exact process tensor method ^46,47^ (see Methods Section).

The working principle of our FTPE scheme can be understood by Floquet theory with respect to the period *T* = 2*π*/*δ*. For detunings *δ* large enough so that *T* ≪ *τ*_0_, fast oscillations can be eliminated and *H* is replaced by a stroboscopic Hamiltonian *H*^eff.^. If moreover *δ* is the dominant energy scale in the Hamiltonian, *δ* ≫ *E*_*b*_/*ℏ*, *f*, the effective Hamiltonian *H*^eff.^ can be obtained by systematic expansion in 1/*δ*^48,49^. This procedure yields a term that directly couples the ground to the biexciton state. The residual impact of the excitonic state is further eliminated by a Schrieffer-Wolff transformation, and we arrive at an effective Hamiltonian involving only ground and biexciton-like states with slowly varying driving envelopes. The details of this calculation can be found in the methods section. Formally, such a two-level system can be regarded as a spin precessing in a fictitious time-dependent effective magnetic field *B*(*t*), which is described by the Hamiltonian

2 $${H}^{{{\rm{eff.}}}}(t)=\frac{\hslash }{2}\left({B}_{x}(t){\sigma }_{x}+{B}_{z}(t){\sigma }_{z}\right).$$

Here *σ*_*x*_ and *σ*_*z*_ are Pauli matrices acting in the reduced two-level subspace spanned by the ground and biexciton states. The *σ*_*x*_ describes the effective coupling between these states, while the *σ*_*z*_ represents their effective detuning.

**Fig. 2:**
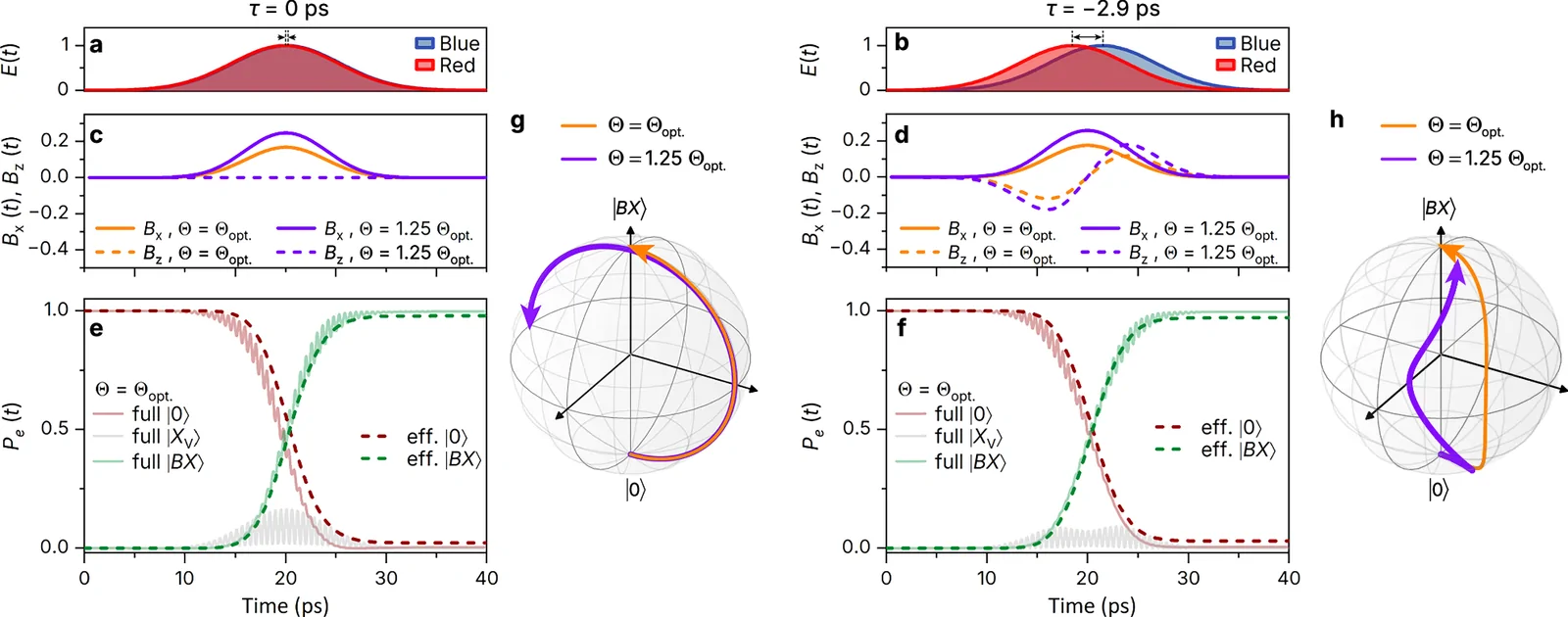
Simulated FTPE for different pulse delays *τ*. **a**, **b** Time-dependent laser amplitudes of red- and blue-detuned pulses for delays *τ* = 0 ps and *τ* = −2.9 ps, respectively. **c**, **d** Time dependence of the effective magnetic field components *B*_*x*_(*t*) and *B*_*z*_(*t*) in the stroboscopic two-level Hamiltonian in Eq. (2) for delays *τ* = 0 and  −2.9 ps. For each delay, we show the result obtained at the optimal driving strength *Θ* = *Θ*_opt._, together with the result for *Θ* = 1.25*Θ*_opt._. The detuning *ℏδ* = 3.75 meV was chosen. **e**, **f** Simulated time evolution of the QD state occupations for the optimal pulse areas corresponding to *τ* = 0 and  −2.9 ps, respectively. Full: solution from the full Hamiltonian of Eq. (1). Eff.: solution from the effective stroboscopic model of Eq. (2). **g**, **h** Corresponding trajectories on the Bloch sphere of the effective two-level system composed of ground and biexciton states.

The effective fields are shaped by a combination of parameters of the exciting lasers, the biexciton binding energy and the detuning. In the special case of zero delay *τ* = 0 between the pulses [Fig. 2a, c], we find *B*_*z*_(*t*) = 0 and thus a Rabi-like dynamics with an effective pulse area $\int_{-\infty }^{t}{B}_{x}(t^{\prime} )dt^{\prime}$ in the two-level system, which is a nonlinear function of the pulse area *Θ* of the original excitation pulses. Interestingly, to leading order, the effective pulse area *Λ* scales as  ~ *Θ*^2^ instead of the usual linear scaling (cf. Eq. (10) in the Methods Section). For finite delay *τ*, *B*_*z*_(*t*) becomes nonzero [Fig. 2b, d], which facilitates the engineering of more complex dynamics.

The time evolution of ground, exciton, and biexciton occupations is shown in Fig. 2e, f for FTPE without and with time delay, respectively, at the optimal pulse area (*Θ*_opt._), which is defined as the pulse area corresponding to the first maximum of the final *BX* occupation. The solid faded lines correspond to the solution of the full Hamiltonian Eq. (1), whereas the dashed lines describe the evolution according to the stroboscopic model of Eq. (2). Except for small-amplitude oscillations, the full numerical solution is well reproduced by the effective stroboscopic model. Thus, the behaviour of the physical system is indeed governed by the engineered effective magnetic field components *B*_*x*_(*t*) and *B*_*z*_(*t*). Finally, Fig. 2g, h show the respective behaviour on the Bloch sphere, of which the south pole corresponds to the ground state $\left\vert 0\right. \rangle$ and the north pole to the *BX* state $\left\vert BX\right. \rangle$. To analyze the robustness of FTPE, we consider not only the optimal driving strengths *Θ*_opt._ but also driving with increased pulse areas 1.25*Θ*_opt._ by scaling the envelopes *f*(*t*) correspondingly. Without delay, *τ* = 0, (left panel) the Bloch sphere trajectories run along a great circle (due to *B*_*z*_ = 0). Thus, if the driving strength deviates from *Θ*_opt._, the *BX* population decreases maximally, as the great circle describes the straightest line on the Bloch sphere. With finite delay *τ* = −2.9 ps, the component *B*_*z*_(*t*) is found to be antisymmetric in time. This component twists the trajectories out of the *yz*-plane. Nevertheless, the *BX* state is reached for pulse area *Θ*_opt._. Further increasing the driving strength not only increases the off-diagonal elements *B*_*x*_ of the stroboscopic model but also the effective detuning *B*_*z*_. The enhanced twisting leads to an elongation of the trajectory from the ground to the *BX* state, such that overshooting, as found for *τ* = 0 ps, is compensated. This mechanism is responsible for the significantly enhanced robustness found for certain delays *τ*.

### Experimental implementation

To verify the experimental feasibility of the proposed FTPE scheme, we carry out the experiment with a single neutral droplet-etched GaAs/AlGaAs QD^50^. The sample is placed in a closed-cycle cryostat and cooled to 3.6 K. Transform-limited Gaussian pulses are generated from a femtosecond laser and stretched into two tunable 12.49-ps pulses using 4-*f* pulse shapers. The time delay *τ* between blue and red pulses is controlled by an optical delay line with a resolution of  ~ 15 fs. The exciton fine-structure splitting (FSS)^51^ due to the QD’s natural anisotropy is effectively eliminated by a continuous-wave (CW) laser via the ac Stark effect^8^. As considered in the theoretical model, the polarizations of lasers are all aligned with the QD’s V dipole orientation, thereby minimizing coupling with the H-polarized exciton $\left\vert {X}_{{{\rm{H}}}}\right\rangle$. More experimental details are provided in the Supplementary Section I.

### Robust biexciton preparation using FTPE

**Fig. 3:**
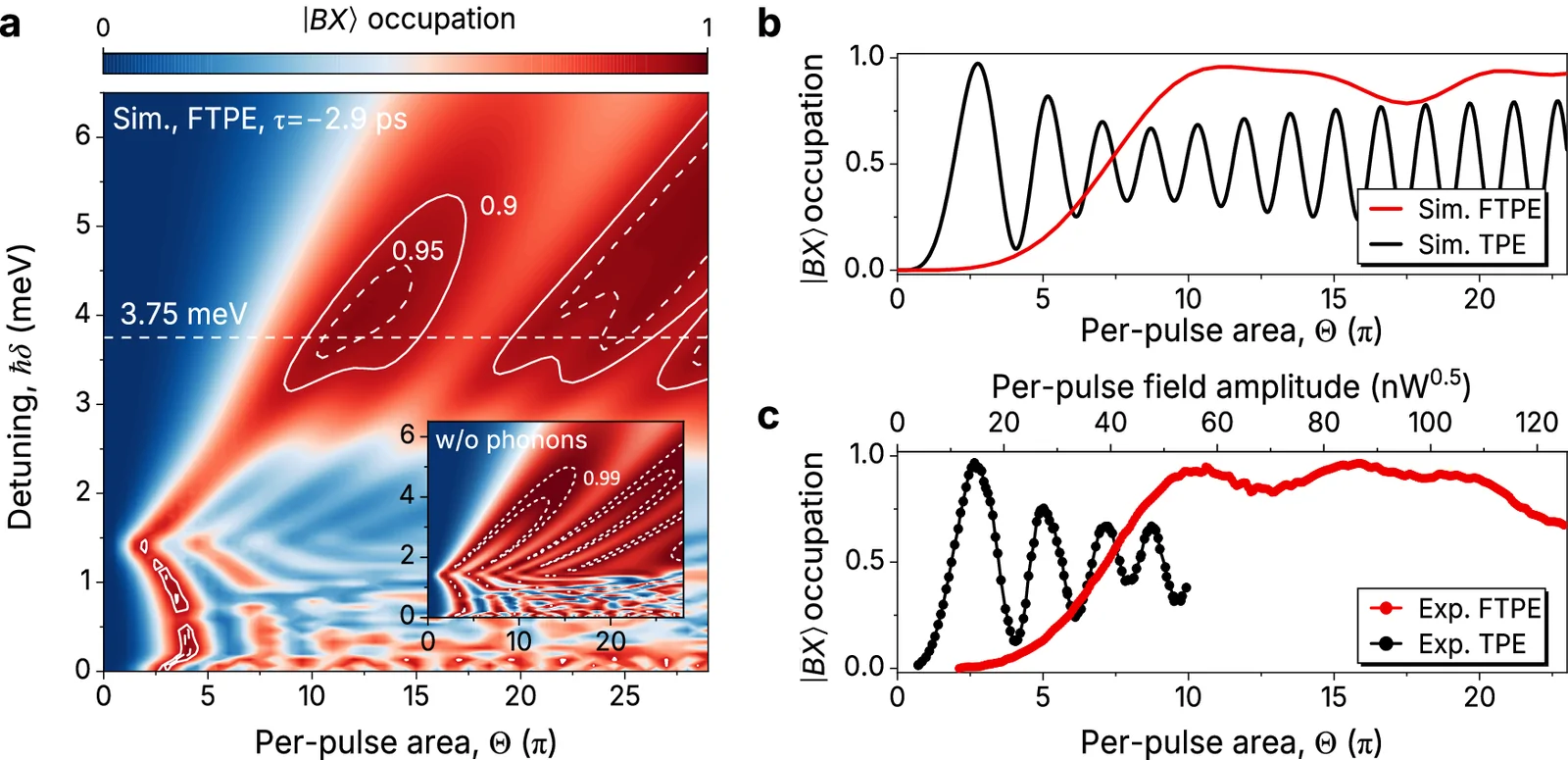
Deterministic and robust biexciton state preparation via FTPE. **a** Calculated *BX* occupation versus per-pulse area *Θ* and detuning *δ* with *τ* = −2.9 ps. The horizontal line is where *ℏδ* = 3.75 meV as used in **b**, **c**. Inset: calculated values without phonon interaction. **b** Theoretically calculated *BX* occupation as a function of the area of each pulse *Θ*, where exciton-phonon interaction and radiative decay are taken into account. **c** Measured intensity of *BX* emission as a function of *Θ* under TPE and FTPE. The experimental *BX* occupation and per-pulse area are normalized with two scaling factors by fitting the TPE curve to simulations (see details in Supplementary Fig. 4).

We first perform a two-dimensional simulation by scanning over the laser pulse area *Θ* and detuning *δ* to map the *BX* state preparation dynamics (Fig. 3a). This allows us to identify parameter regimes that enable efficient and robust *BX* state preparation. Qualitatively different parameter regimes can be identified: For TPE with small detunings, biexciton population is found at certain spots but the results are very sensitive to parameter fluctuations. At larger detunings, cusps at *ℏδ* = *E*_*b*_/2 indicate the simultaneous resonance of the lasers with the $\left\vert 0\right. \rangle$ to $\left\vert {X}_{V}\right. \rangle$ and $\left\vert {X}_{V}\right. \rangle$ to $\left\vert BX\right. \rangle$ transitions. Increasing the detuning further to about *ℏδ* ≈ *E*_*b*_, one reaches the FTPE regime. We note that low-order Magnus expansion captures only the FTPE regime well. The stroboscopic Floquet Hamiltonian describes the dynamics appropriately, down to small detunings where the pulse envelope no longer changes slowly with respect to 1/*δ* (see Supplementary Section VII). Notably, TPE theory^12^ involves an adiabatic approximation that breaks down for the large detunings required for FTPE (see Supplementary Section X A).

The FTPE results show that near-unity preparation efficiency is achievable despite the presence of phonons, and high efficiency ( > 90%) can be maintained across a wide range of excitation conditions, demonstrating the robustness of the scheme. FTPE can also be robust against spectral shifts, as we show in Supplementary Section IX B. The theoretical model is described in the Methods Section, and it includes the full time-dependent Hamiltonian, radiative decay, as well as non-Markovian phonon effects, which are captured within the numerically exact process tensor framework ACE^46,47^. The inset of Fig. 3a shows calculated results without phonon effects, confirming that phonon assistance is not essential for *BX* preparation. We attribute detrimental effects due to phonon coupling to phonon-assisted population of the exciton state, which are significant for detunings below about 3 meV, where the coupling to phonons is strongest. Choosing a larger detuning is therefore advisable to decouple from phonon effects. Without phonons, the reachable biexciton occupations are even higher, making the scheme even more attractive for emitters not affected by phonons, such as atomic systems.

Having identified the suitable laser pulse parameters, we next perform an experimental demonstration of FTPE. Figure 3c shows experimentally measured *BX* emission (red dots) obtained under FTPE with *ℏδ* = 3.75 meV. With increasing laser pulse area, the *BX* emission exhibits a gradual increase followed by a slightly fluctuating plateau. As a comparison, the *BX* emission (black dots) obtained under conventional TPE shows the well-known Rabi oscillation. The main damping source of the Rabi oscillations comes from the QD-phonon coupling^52^. The experimentally achieved occupations and pulse area are calibrated by fitting the TPE Rabi oscillation data to numerical results with two scaling factors, as shown in Supplementary Fig. 4. Both excitation schemes are well reproduced by our model [Fig. 3b]. As shown in Fig. 3c, the first maximum of the *BX* occupation under TPE (black dots, 96.8%) occurs at a per-pulse area of 2.7*π*. The maximum *BX* occupation achieved under FTPE is 96.6% and appears at a per-pulse area of 15.9*π*. Importantly, the achieved final occupation shows significantly reduced sensitivity to variations in laser amplitude. This robustness to experimental fluctuations underscores the advantages of the FTPE scheme for practical quantum information applications. The small discrepancy between the theoretical and experimental peak occupations may arise from the uncertainty in the effective phonon-coupling coefficient extraction, as discussed in Supplementary Section IV. The slight decrease of the experimental curve in the high-power regime may be related to additional excited-state channels, such as hole-state excitation (Supplementary Section VI), that are not included in the present model.

**Fig. 4:**
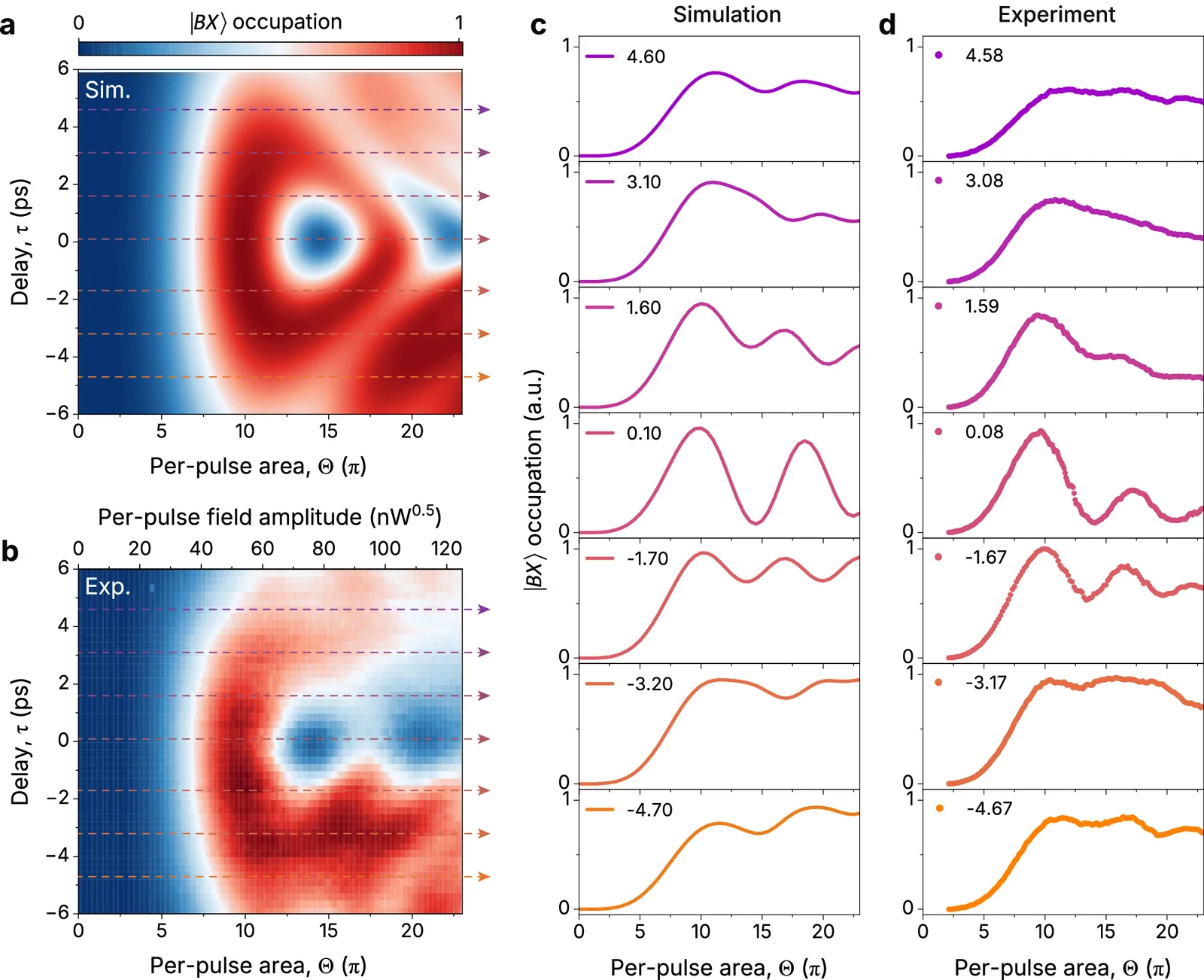
Pulse delay dependence of FTPE. **a**, **b** Two-dimensional maps of the calculated **a** and measured **b** *BX* occupation as functions of the per-pulse area *Θ* and pulse delay *τ*, with *ℏδ* = 3.75 meV. **c** Line cuts extracted from the calculated map in **a**, showing the calculated *BX* occupation as a function of the per-pulse area for selected pulse delays ranging from 4.60 to −4.70 ps. **d** Line cuts extracted from the measured map in **b**, showing the measured *BX* occupation as a function of the per-pulse area for selected pulse delays ranging from 4.58 to −4.67 ps.

The temporal separation between the two pulses plays a key role in achieving the robustness of FTPE. To comprehensively investigate the influence of this delay on FTPE performance, we simulate and measure the dependence of *BX* occupation on the pulse area for various two-pulse delays. Figure 4a presents a simulated two-dimensional colour map of *BX* occupation as a function of pulse area and time delay. The detuning *ℏδ* is fixed at 3.75 meV, consistent with the experimental condition. The asymmetry with respect to zero delay observed in the colour map arises from the LA-phonon-assisted excitation of the exciton^27^. For delays *τ* > 0, the blue pulse arrives first, populating the exciton state and thereby hindering the two-photon excitation process. A simulation excluding phonon effects is shown in Supplementary Fig. 2, where the delay dependence recovers a symmetric pattern. The corresponding experimentally measured two-dimensional map is presented in Fig. 4b, which reproduces the main features predicted by the simulation. Figure 4c, d show line cuts taken from Fig. 4a, b, respectively, at selected delays. For *τ* < 0, the red-detuned pulse arrives first, such that competition from phonon-assisted excitation becomes negligible. In contrast to the pronounced Rabi-oscillation behavior observed near *τ* = 0 ps, the *BX* occupation becomes more robust against variations in pulse area over a finite range of negative delay. From the experimental results in Fig. 4d, we identify *τ* = −2.5 to −4.2 ps as the experimentally optimal delay range.

### On-demand generation of entangled photons

**Fig. 5:**
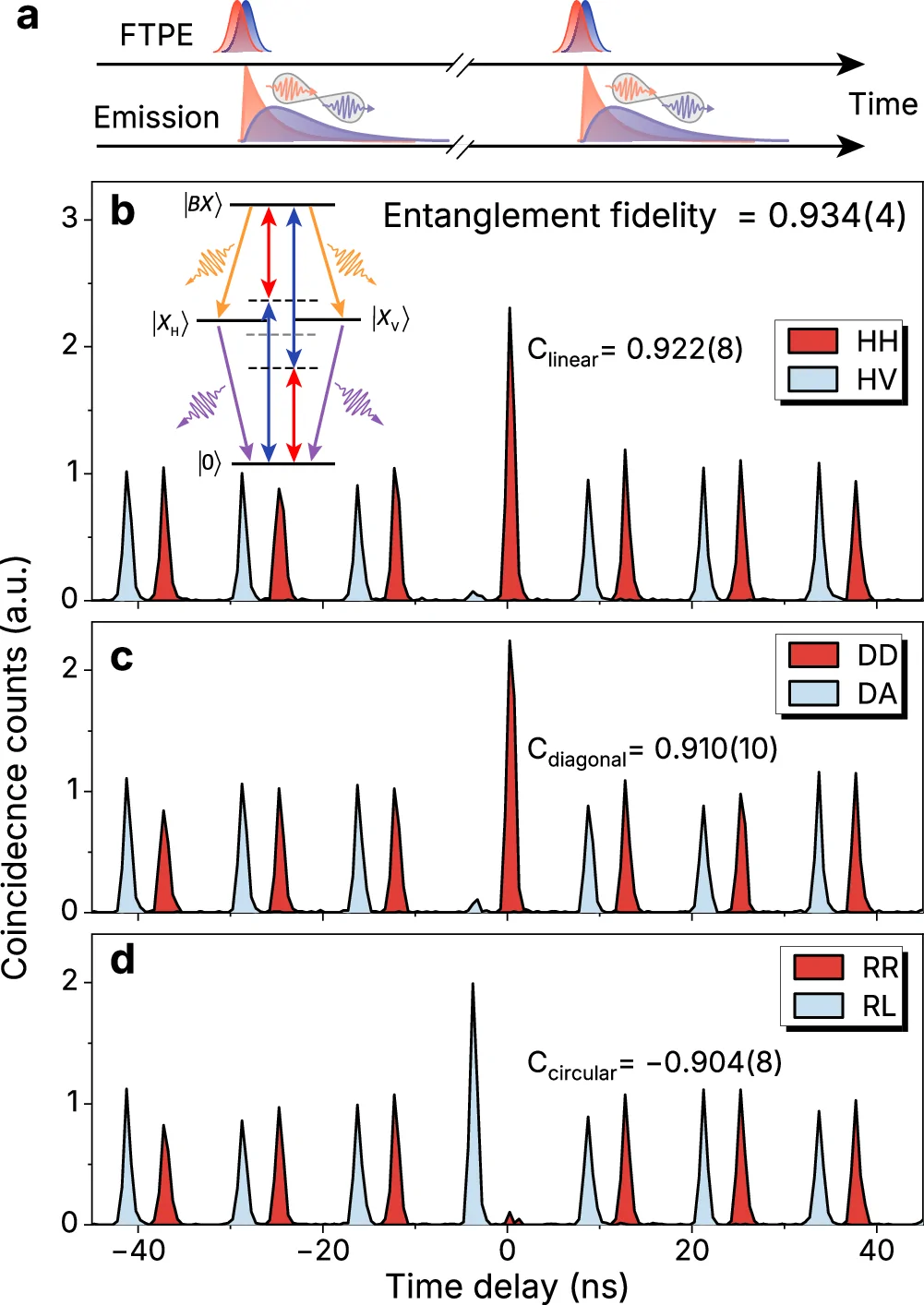
Entanglement characterization under FTPE. **a** Schematic of polarization entangled photon pairs generated by FTPE. **b** Detected *BX*-*X* photon cross-correlation coincidence counts under FTPE in linear basis (H, horizontal; V, vertical). Inset: The cascade radiative recombination of *BX* state provides two polarization-entangled photons. **c** In diagonal basis (D, diagonal; A, anti-diagonal). **d** In circular basis (R, right circular; L, left circular).

One promising application of our FTPE scheme is to deterministically generate entangled photon pairs. To assess the practicality, it is crucial to examine both the entanglement fidelity of the FTPE-generated photon pairs and the compatibility of the FTPE scheme with experimental techniques required for the collection and verification of entangled photons. To this end, we investigate the cascade emission from the *BX* state prepared by FTPE, which is operated as a source of polarization-entangled photon pairs [Fig. 5a, b inset]. Since the FTPE pulses are far detuned from *BX* and *X* photon emission peaks, they can be removed simply by spectral filters without affecting the polarization state of entangled photons. The entanglement fidelity is then characterized by separating *BX* and *X* photons using a polarization-insensitive grating, followed by the measurement of the polarization-dependent second-order correlation functions. The intrinsic FSS of 3.4(2) μeV is eliminated by the ac Stark effect induced by the 13.5 μW CW laser with a red detuning of 300 μeV ^8^. Figure 5 shows six cross-correlation measurements in the rectilinear [HH and HV, Fig. 5b], diagonal [DD and DA, Fig. 5c], and circular [RR and RL, Fig. 5d] polarization bases under FTPE, with each histogram composed of 500-ps time bins. By analyzing the degrees of polarization correlation (*C*_*μ*_, where *μ* is the polarization basis), the fidelity to the Bell state $\left\vert {\psi }^{+}\right\rangle=\frac{1}{\sqrt{2}}\left(\left\vert {H}_{BX}\right\rangle \left\vert {H}_{X}\right\rangle+\left\vert {V}_{BX}\right\rangle \left\vert {V}_{X}\right\rangle \right)$ is obtained as:

3 $$f=\frac{1+{C}_{{{\rm{linear}}}}+{C}_{{{\rm{diagonal}}}}-{C}_{{{\rm{circular}}}}}{4}=0.934(4).$$

The uncertainties are calculated using error propagation, assuming Poisson statistics for the coincidence counts. In the idealized calculation without FSS and the CW laser, the fidelity reaches 97.7% (Supplementary Section VIII), with the remaining 2.3 percentage-point reduction from unity caused by the pulse-induced transient spectral shift. Including the CW laser reduces the calculated fidelity further to 95.3%, close to the experimentally measured value of 93.4%. This additional reduction is associated with CW-induced state dressing, which is also supported by the power dependence of the autocorrelation measurements (see Supplementary Section III). Importantly, the FTPE protocol is compatible with other established FSS-erasure techniques, such as strain^7^, magnetic-field^53^, and static electric-field^54^ tuning, which could avoid the associated state dressing and thereby enable higher entanglement fidelity. These results demonstrate that the FTPE protocol is a reliable method for generating highly entangled photon pairs.

## Discussion and conclusion

We have demonstrated the design and implementation of a robust biexciton state preparation scheme for a semiconductor quantum dot based on our concept of ultrafast all-optical Floquet engineering. Appropriately choosing the parameters of two differently coloured laser pulses lets us arrange a situation where the quantum dot experiences periodic driving within the laser duration. This allows us to engineer a stroboscopic Hamiltonian connecting the initial state with the target state of the protocol and to control the effective time-dependent driving between them. Thereby, we can identify a suitable parameter regime where FTPE is robust against fluctuations in the laser power and where detrimental phonon effects are also of minor importance. In particular, we have identified the delay between the two pulses to be a pivotal tuning knob for reaching the robust regime. The theoretical predictions are well reproduced in our experiments.

The selectable laser detuning of FTPE can provide greater experimental flexibility and relax the demand for laser filtering. In the present weakly confined GaAs/AlGaAs QD, the practical detuning window is finite but remains sizable ( ≳ 1 meV, Supplementary Section VI). This restriction is specific to the present QD system rather than an intrinsic limitation of FTPE. Moreover, the high entanglement fidelity achieved suggests that the FTPE scheme does not induce additional dephasing channels, further validating its practicality for quantum information applications. Notably, the laser pulse parameters used in this work, such as duration, waveform, and chirp, can be further optimized. Future improvements could be realized through quantum optimal control^55^ by systematically tailoring these parameters to enhance the efficiency and robustness of state preparation.

In Table 1, we provide a comparison of FTPE with representative excitation schemes used for entangled-photon generation in QDs. FTPE offers a highly robust and filtering-friendly route for generating entangled photon pairs, while maintaining high fidelity and preparation efficiency comparable to those of conventional TPE. As also detailed in the Supplementary Section X, the proposed FTPE scheme is distinct from other excitation schemes: the parameter regime for FTPE with detuning *δ* > *E*_*b*_/(2*ℏ*) is opposite to the limit of resonant TPE, which can be generalized to small but nonzero detuning *δ* ≪ *E*_*b*_/(2*ℏ*). FTPE works almost the same when the binding energy is negative. As binding energy decreases, more laser power is needed and for zero binding energy no robust excitation can be obtained. Moreover, although robustness is also a feature found in adiabatic protocols, especially in the most robust region of finite pulse delays, FTPE is not adiabatic on an instantaneous eigenstate basis. The optical selection rules differ from stimulated Raman adiabatic passage (STIRAP), and the resonance conditions of FTPE differ from those in other two-colour excitation schemes such as SUPER. These unique characteristics collectively highlight the distinctions of FTPE. Hence, the concept of all-optical single-shot Floquet driving provides a basis for developing platform-tailored excitation protocols for quantum emitters with suitable ladder-type structure, whenever one wants to drive transitions between states that are only indirectly coupled via off-resonant intermediate states. The essential requirements are compatible optical selection rules, sufficiently large but experimentally accessible detunings to suppress real occupation of the intermediate state while retaining effective coupling, and coherence times long compared with the single-shot driving window. Beyond the semiconductor QD system demonstrated here, this concept may also be applicable to selected molecular^56^, atomic^57^, colour-center^58^, and superconducting artificial-atom systems^59^.

**Table 1:** Comparison of existing *BX* preparation schemes in semiconductor QDs

Scheme	Preparation efficiency		Entanglement fidelity	Ref.	Pulse-area robustness	Filtering difficulty
	Theory	Experiment	Experiment			
TPE	> 99% without phonons	~ 82%	–	[^12^]	Weak	Moderate
	–	~ 90%	88%	[^14^]		
	–	n.q.	97.8%	[^7^]		
Phonon-assisted TPE	> 99%	–	–	[^44^]	Strong	Difficult
	–	80%	90%	[^29^]		
	–	95%	–	[^28^]		
	–	> 80%	–	[^67^]		
ARP	> 90% at 4 K > 98% at 1 K	–	–	[^24^]	Strong	Difficult
	–	> 95%	–	[^68^]		
SUPER	> 99% without phonons	–	–	[^69^]	Moderate	Easy
	> 95% at 4 K	–	–	[^30^]		
	–	n.q.	–	[^70^]		
FTPE	> 99% without phonons 96.7% at 3.6 K	96.6%	93.4% (97.7%)^*a*^	This work	Strong	Easy

^*a*^ Ideal fidelity without FSS and CW laser. n.q., not quantified. –, not reported.

## Methods

### Numerical methods

In our simulations, we include longitudinal acoustic (LA) phonons. Their coupling to the QD is included using the Hamiltonian

4 $${H}_{{{\bf{ph}}}}/\hslash={\sum}_{{{\bf{q}}}}{\omega }_{{{\bf{q}}}}{b}_{{{\bf{q}}}}^{{\dagger} }{b}_{{{\bf{q}}}}+{\sum}_{{{\bf{q}}},\nu }{n}_{\nu }\left({g}_{{{\bf{q}}}}{b}_{{{\bf{q}}}}+{g}_{{{\bf{q}}}}^{*}{b}_{{{\bf{q}}}}^{{\dagger} }\right)\left\vert \nu \right\rangle \left\langle \nu \right\vert,$$

where ${b}_{{{\bf{q}}}}^{{\dagger} }\,({b}_{{{\bf{q}}}})$ is the creation (annihilation) operator for a LA phonon with wave vector **q** and frequency *ω*_**q**_, $\left\vert \nu \right\rangle \in \left\{\left\vert {X}_{V}\right\rangle,\left\vert BX\right\rangle \right\}$ is the excitonic state and *n*_*ν*_ is the number of excitons present in the state $\left\vert \nu \right\rangle$. *g*_**q**_ denotes the coupling constant, which depends on crystal mass density, mode volume, material deformation potential and so on. We use these material parameters taken from the literature^60^ and leave the electron (hole) wavefunction radius as the only free parameter, where the wavefunctions correspond to a harmonic confinement potential.

The radius of the electronic wavefunction is determined by fitting the calculated pulse-area dependence of the $\left\vert BX\right\rangle$-occupation for TPE to the experimental TPE results, which are displayed in Fig. 3b, c. We obtain the radius *a*_*e*_ = 5.5 nm. For the corresponding hole radius, we use *a*_*h*_ = *a*_*e*_/1.15. All subsequent FTPE calculations were done using these values.

We solve the combined QD-phonon Hamiltonian using a state-of-the-art process tensor method^47,61,62^. This is necessary to accurately include phonons because it allows for short time-step lengths, which is necessary in this case due to the potentially large detunings *δ*. Other numerically complete methods, in particular, path-integral methods^63^ would demand unfeasibly large amounts of memory.

### Derivation of the effective Hamiltonian

Here, we summarize the mapping from the two-colour two-photon excitation of the three-level system composed of ground, exciton and biexciton states to an effective two-level system consisting predominantly of ground and biexciton states. We focus on the regime where the detuning *δ* is the dominant frequency scale. Then, the envelopes *f*(*t* ± *τ*/2) in the driving term *Ω*(*t*) = *f*(*t* − *τ*/2)*e*^−*iδ*(*t*−*τ*/2)^ + *f*(*t* + *τ*/2)*e*^*iδ*(*t*+*τ*/2)^ vary slowly during a single oscillation period *T* = 2*π*/*δ*.

To separate the time scales, we first assume the envelopes *f*(*t*) ≈ *f* to be constant over time *T*. We seek a stroboscopic Hamiltonian $\bar{H}$, which is similarly constant over short times *t* ≲ *T*, but reproduces the time evolution over a single oscillation period *T*. It is defined by the condition

5 $$U(T)={e}^{-\frac{i}{\hslash }\bar{H}T}{=}^{!}{{\mathcal{T}}}e^{-\frac{i}{\hslash }{{\int}_{0}^{T}}H(t)dt},$$

where ${{\mathcal{T}}}$ denotes the time-ordering operator. The stroboscopic Hamiltonian $\bar{H}=\bar{H}(f)$ parametrically depends on the pulse envelope *f*.

Thus, the dependence of $\bar{H}$ on a coarse-grained time *t* can be reintroduced via the parametric dependence $\bar{H}(t)=\bar{H}(f(t))$.

While it is straightforward to obtain $\bar{H}$ for a fixed value of *f* numerically, analytic insights can be gained by considering the Magnus expansion $\bar{H}=\mathop{\sum }_{n=0}^{\infty }{\bar{H}}^{(n)}$. The leading-order terms are given by^49^

6a $${\bar{H}}^{(0)}=\frac{1}{T}{{\int}_{0}^{T}}d{t}_{1}\,H({t}_{1})$$

6b $${\bar{H}}^{(1)}=\frac{-i}{2\hslash T}{{\int}_{0}^{T}}d{t}_{1}{{\int}_{0}^{t1}}d{t}_{2}\,\left[H({t}_{1}),H({t}_{2})\right],$$

6c $$\begin{array}{l}{\bar{H}}^{(2)}=\frac{-1}{6{\hslash }^{2}T}{{\int}_{0}^{T}}d{t}_{1}{{\int}_{0}^{{t}_{1}}}d{t}_{2}{\int}_{0}^{{t}_{2}}d{t}_{3}\,\left(\left[H({t}_{1}),[H({t}_{2}),H({t}_{3})]\right]\right.\\ \left.+\left[H({t}_{3}),[H({t}_{2}),H({t}_{1})]\right]\right),\end{array}$$

6d $$\begin{array}{l}{\bar{H}}^{(3)}=\frac{i}{12{\hslash }^{3}T}{{\int}_{0}^{T}}d{t}_{1}{{\int}_{0}^{{t}_{1}}}d{t}_{2}{{\int}_{0}^{{t}_{2}}}d{t}_{3}{{\int}_{0}^{{t}_{3}}}d{t}_{4}\\ \left(\left[\left[\left[H({t}_{1}),H({t}_{2})\right],H({t}_{3})\right],H({t}_{4})\right]\right.\\+\left[H({t}_{1}),\left[[H({t}_{2}),H({t}_{3})],H({t}_{4})\right]\right]\\+\left[H({t}_{1}),\left[H({t}_{2}),[H({t}_{3}),H({t}_{4})]\right]\right]\\ \left.+\left[H({t}_{2}),\left[H({t}_{3}),[H({t}_{4}),H({t}_{1})]\right]\right]\right).\end{array}$$

One finds that, to zeroth order, the dynamics on a coarse-grained timescale is governed by the time-averaged Hamiltonian ${\bar{H}}^{(0)}$. Yet the commutators in the higher-order terms can lead to qualitatively different transitions that are not directly visible from the original Hamiltonian *H*(*t*).

For example, if there is no delay between the two pulses, $\Omega (t)=2f(t)\cos (\delta t)$ and

7a $${\bar{H}}^{(0)}=\left(\begin{array}{lll}0&0&0\\ 0&{E}_{b}/2&0\\ 0&0&0\end{array}\right),$$

7b $${\bar{H}}^{(1)}=\,0,$$

7c $${\bar{H}}^{(2)}=\,\frac{{E}_{b}f}{4\hslash {\delta }^{2}}\left[\hslash f\left(\begin{array}{ccc}1&0&1\\ 0&-2&0\\ 1&0&1\end{array}\right)-\frac{{E}_{b}}{2}\left(\begin{array}{ccc}0&1&0\\ 1&0&1\\ 0&1&0\end{array}\right)\right].$$

The zero-order term ensures that the exciton state remains off-resonant by energy *E*_*b*_/2. Because *n*-th order terms scale as 1/*δ*^*n*^ with the detuning *δ*, the zero-order term ensures that the excitonic state only becomes weakly occupied during the dynamics in the regime of interest where *δ* dominates. The first-order term vanishes when there is no delay between the pulses. Thus, the dynamics is mainly determined by the second-order term ${\bar{H}}^{(2)}$, which itself consists of two contributions. The first provides corrections to the diagonal energies but more importantly drives direct transitions between the ground and the biexciton state with an effective Rabi frequency *E*_*b*_*f*^2^(*t*)/(2*ℏδ*^2^). The second contribution describes conventional transitions between ground and exciton and between exciton and biexciton states. However, as the exciton state is strongly off-resonant, the latter processes remain largely virtual and only lead to small quantitative corrections to the dynamics.

In fact, we find that the exciton state remains very weakly occupied even in the more complex case of finite delay between the pulses. This makes it possible to eliminate the exciton state altogether by a Schrieffer-Wolff transformation^64,65^. There, the basis is slightly rotated by a unitary transformation such that coupling terms between the third, predominantly excitonic state and the remaining two states, which are predominantly composed of the ground and biexciton state, vanish to leading order. To the second order, the effective two-level Hamiltonian is constructed from the stroboscopic Hamiltonian $\bar{H}$ by

8 $${H}^{{{\rm{eff}}}.}_{m,n}={{\bar{H}}_{m,n}}+\frac{1}{2}\left(\frac{{\bar{H}}_{m,{X}_{V}}{\bar{H}}_{{X}_{V},n}}{{\bar{H}}_{m,m}^{(0)}-{\bar{H}}_{{X}_{V},{X}_{V}}^{(0)}}+\frac{{\bar{H}}_{m,{X}_{V}}{\bar{H}}_{{X}_{V},n}}{{\bar{H}}_{n,n}^{(0)}-{\bar{H}}_{{X}_{V},{X}_{V}}^{(0)}}\right),$$

where *n*, *m* ∈ {*BX*, 0}. Higher-order Schrieffer-Wolff contributions are doubly suppressed, on the one hand by factors 1/*δ*^*n*^ in the numerator, which stems from higher-order Magnus expansion terms (valid for *δ* ≫ *E*_*b*_/(2*ℏ*)), and on the other hand by factors ${E}_{b}^{n}$ in the Schrieffer-Wolff denominators (valid when *E*_*b*_ is much larger than the prefactors in ${\bar{H}}^{(m)}$ for *m* > 0). Doing this and introducing the time dependence of the laser envelopes, *f* → *f*(*t*), we obtain the effective Hamiltonian ${H}^{{{\rm{eff}}}.}=\frac{\hslash }{2}({B}_{x}(t){\sigma }_{x}+{B}_{z}(t){\sigma }_{z})$ with components *B*_*x*_(*t*) and *B*_*z*_(*t*) obtained by calculating the inner product

9 $${B}_{x/z}(t)=\frac{1}{\hslash }{{\rm{Tr}}}[{\sigma }_{x/z}{H}^{{{\rm{eff.}}}}].$$

For the case without delay, we can now also derive analytical formulas for the approximate biexciton occupation for different pulse amplitudes, using the well-known expressions from resonant Rabi systems. As was seen in Fig. 2 of the main text, the diagonal elements of (8) are zero for *τ* = 0 ps, which gives an effective pulse area of

10 $${\varLambda} :=\frac{2}{\hslash }\int_{-\infty }^{\infty }{H}_{BX,0}^{{{\rm{eff.}}}}(t)\,{{\rm{d}}}t=\frac{{E}_{b}}{4\sqrt{\pi }\hslash {\delta }^{2}s}\left(1-\frac{{E}_{b}^{2}}{8{\hslash }^{2}{\delta }^{2}}\right) {\varTheta }^{2}.$$

Using this, it is possible to calculate the final biexciton occupation using

11 $$| \langle BX| \psi (t=\infty )\rangle {| }^{2}={\sin }^{2}\left(\frac{\varLambda }{2}\right).$$

Importantly, the effective pulse area *Λ* scales with the square of the original pulse area *Θ*, which was used for the two pulses.

## Supplementary information

Supplementary Information

Transparent Peer Review file

## Data Availability

The raw data that support the findings of this study are available on Zenodo at https://doi.org/10.5281/zenodo.21787444^66^.

## Code availability

The code that has been used for this work is available from the corresponding authors upon request.
