# Supplementary material for: Robust biexciton preparation for entangled photon-pair generation by single-shot Floquet driving
Source: Nat Commun. 2026 Sep 12;17:9745. doi: 10.1038/s41467-026-77221-9 (PMC13570888; doi:10.1038/s41467-026-77221-9)
Supplement: Supplementary file 1 [file 41467_2026_77221_MOESM1_ESM.pdf]

Supplementary information: Robust biexciton preparation for entangled photon-pair generation by single-shot Floquet driving

Jun-Yong Yan*, Paul C. A. Hagen*, Hang-Yu Ge, Fang-Yuan Li, Hans-Georg Babin, Wei E. I. Sha, Bo Wang, Zhi-hua Gan, Andreas D. Wieck, Arne Ludwig, Chao-Yuan Jin, Vollrath Martin Axt, Da-Wei Wang, Moritz Cygorek†, and Feng Liu‡

CONTENTS

I. Experimental setup 2

II. Biexciton preparation efficiency at different pulse delays without phonon 3

III. Tuning QD FSS via ac Stark effect 3

IV. Calibration of pulse area, occupation, and electronic wavefunction radius 4

V. Parameters used in the calculations 4

VI. Determination of suitable detuning for FTPE in GaAs QD 5

VII. Validity of the Floquet model 6

VIII. Theoretical entanglement fidelity 7

IX. Robustness of FTPE against parameter variations 8
- A. Influence of the pulse delay 8
- B. Influence of spectral shifts 9
- C. Influence of pulse-area imbalance 9

X. Comparison with other excitation mechanisms 11
- A. Nearly monochromatic two-photon resonant excitation 11
- B. Adiabatic protocols 11
- C. STIRAP 12
- D. SUPER and dichromatic excitation 13

XI. Relative phase difference between the lasers 14

References 15

I. EXPERIMENTAL SETUP

A schematic of the optical characterization setup is illustrated in Supplementary Fig. 1. The QD sample is placed in the 3.6-K cryostat (attocube). Transform-limited femtosecond pulses are generated by a Ti:sapphire oscillator (Coherent) and subsequently split and shaped into a pair of pulses with different colours (denoted blue and red) by two pulse shapers. The temporal delay between the blue and red pulses is controlled by an optical delay line (Newport). A tunable CW laser (M squared) is set to be slightly red-detuned with respect to the $|BX\rangle \rightarrow |X_V\rangle$ transition and used to tune the QD FSS. The polarization of both the pulsed and CW lasers is aligned with the V dipole orientation of the QD, ensuring minimal coupling to the H dipole.

The emitted photons are collected by an aspheric objective lens (Thorlabs) and directed to a custom-built entanglement measurement setup. A filtering system, comprising a volume phase holographic transmission grating (Coherent) and notch filters (Optigrate), is employed to spectrally eliminate laser scattering and separate the BX and X photons. A set of wave plates (Thorlabs), including two QWPs and one HWP, is used to compensate for polarization misalignment between the QD emission and the tomography basis [S1, S2]. Finally, the entangled photons are projected onto different polarization bases and then detected by two single-photon avalanche detectors (Excelitas) for cross-correlation measurements.

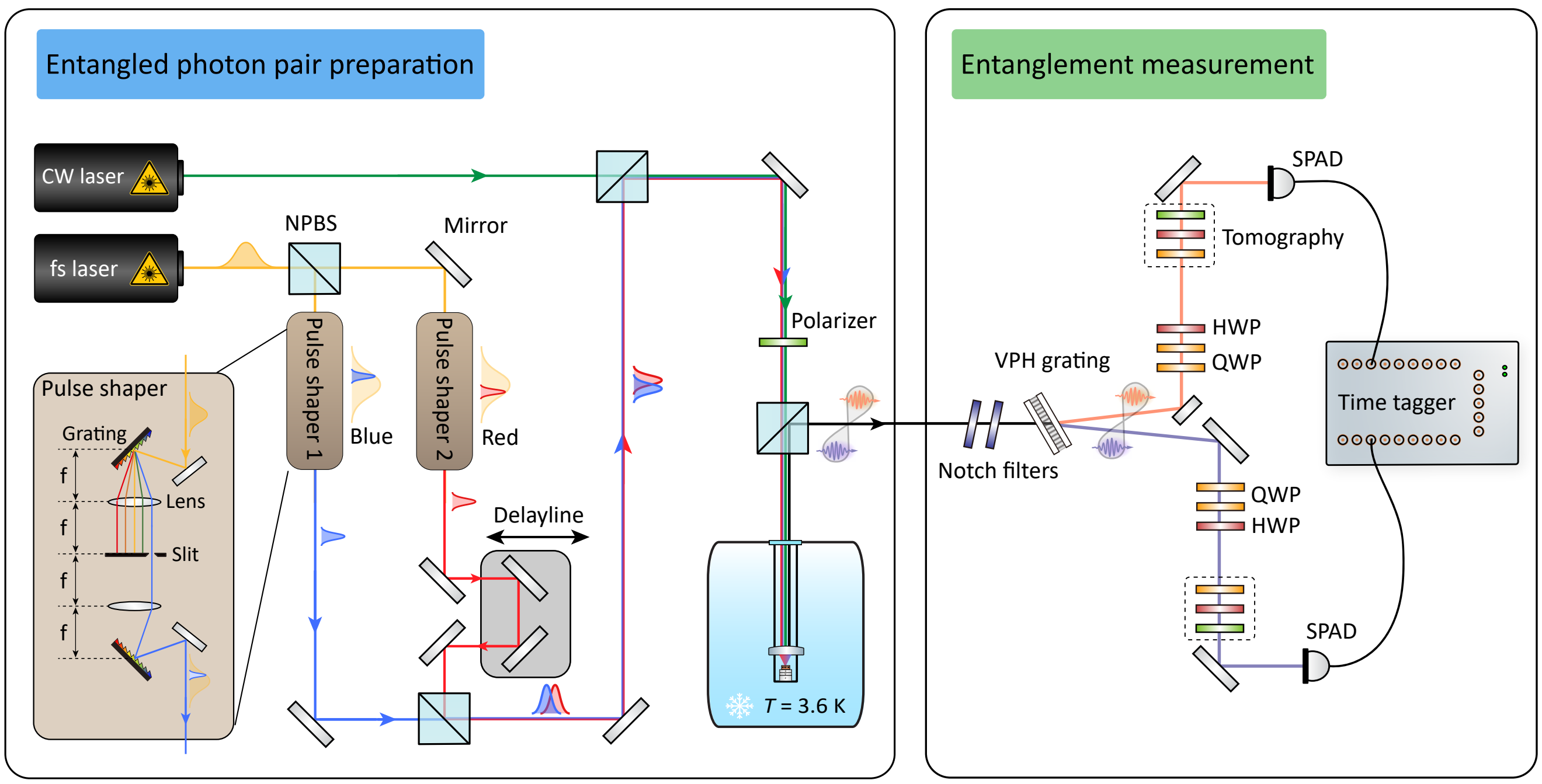

Supplementary Figure 1. **Sketch of the experimental setup.** The setup consists of two main parts: entangled photon pair preparation and entanglement measurements. NPBS: non-polarizing beam splitter; VPH grating: volume phase holographic grating; HWP: half-wave plate; QWP: quarter-wave plate; SPAD: single-photon avalanche diode.

II. BIEXCITON PREPARATION EFFICIENCY AT DIFFERENT PULSE DELAYS WITHOUT PHONON

We also simulated the BX occupation's dependence on pulse area at different pulse delays, without considering QD-phonon coupling. As shown in Supplementary Fig. 2, a symmetric two-dimensional pattern about delay $\tau = 0$ ps is recovered.

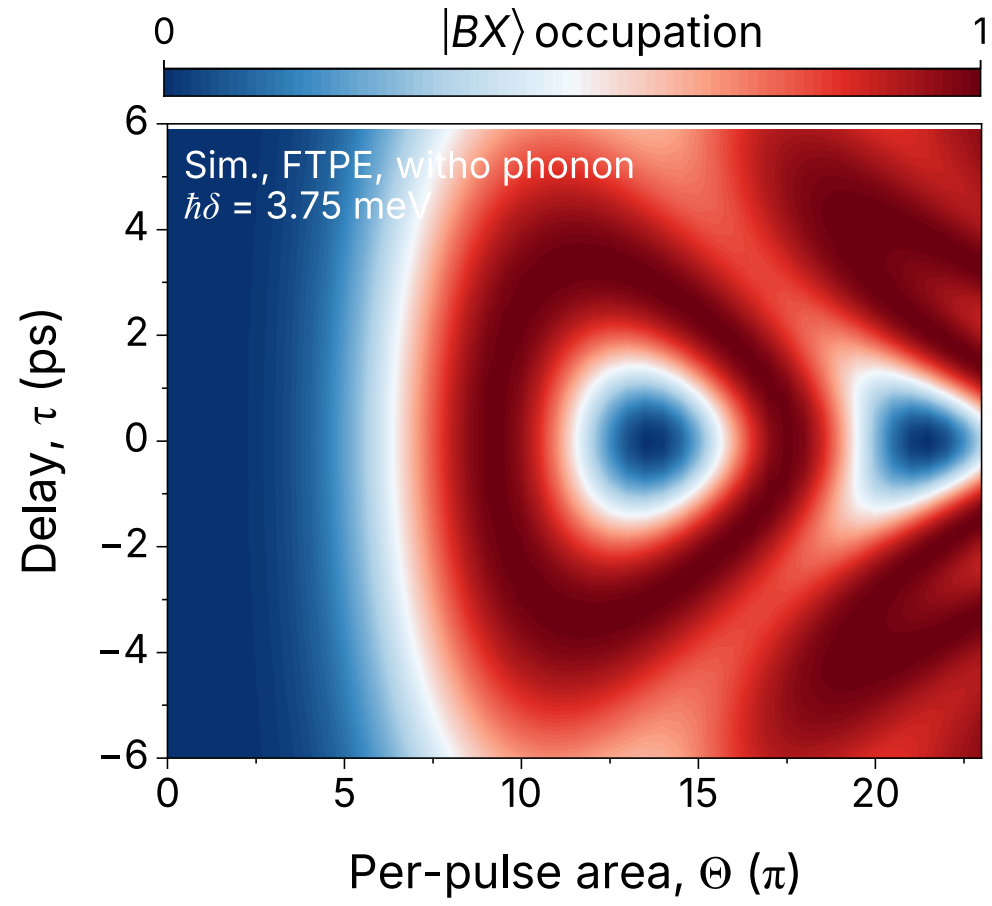

Supplementary Figure 2. **Biexciton preparation efficiency at different pulse delays** Calculated BX occupation versus Θ and τ without considering phonon effect.

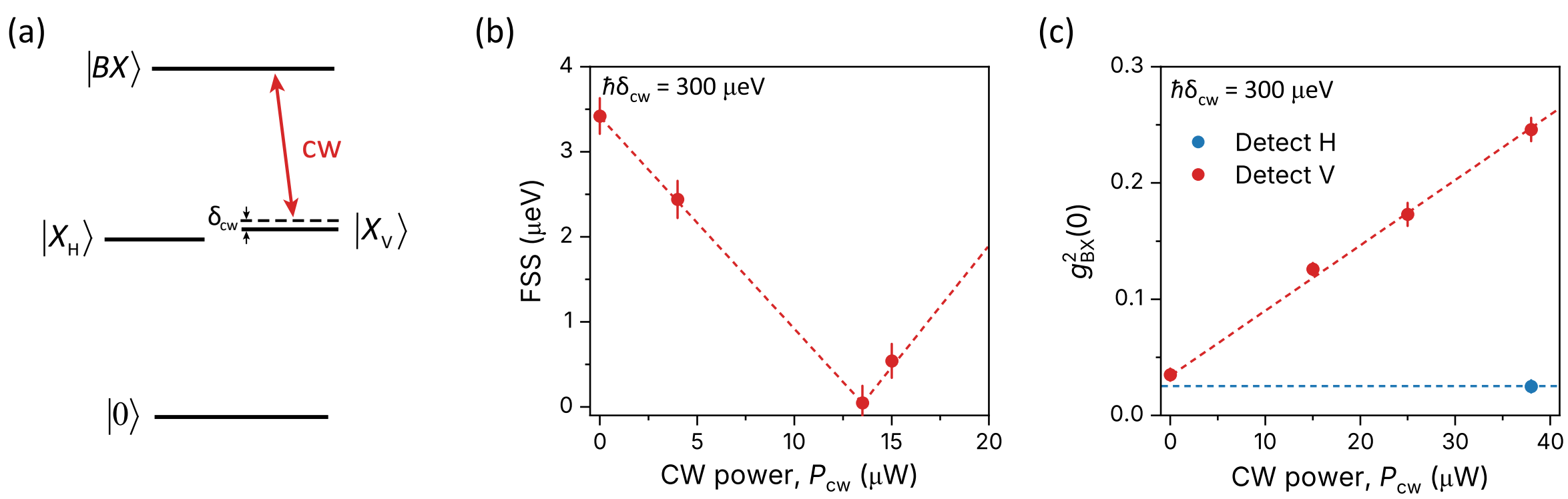

Supplementary Figure 3. **FSS erasure via ac Stark effect.** **(a)**, ac Stark effect and QD energy level diagram. **(b)**, FSS as a function of applied CW laser power. Error bars arise from the standard error of sine fit residuals. **(c)**, Polarization-resolved autocorrelation measurements of $|BX\rangle \rightarrow |X\rangle$ photons.

III. TUNING QD FSS VIA AC STARK EFFECT

To eliminate the FSS of the QD, we apply a vertical linear polarized CW laser slightly detuned ($\hbar\delta_{\text{cw}} = 300\ \mu eV$) from the $|BX\rangle \leftrightarrow |X_{\text{V}}\rangle$ transition (Supplementary Fig. 3a). This allows us to tune the FSS by varying the power of the CW laser according to:

$$\Delta_{\text{FSS}} = \frac{\hbar}{2}\left(\delta_{\text{CW}} - \sqrt{{\delta_{\text{CW}}}^2 + \Omega^2}\right). \tag{S1}$$

The QD investigated in the main text is QD A. Supplementary Fig. 3(b) presents the experimentally measured FSS as a function of CW laser power. An optimal operating power of 13.5 μW is identified, where the FSS is almost eliminated. However, we observe a reduction in the single-photon purity of the $|BX\rangle \rightarrow |X_{\text{V}}\rangle$ photon pair with

increasing CW power (Supplementary Fig. 3(c)). We attribute this effect to CW-induced state dressing between $|X_V\rangle$ and $|BX\rangle$, which can lead to twice $|BX\rangle$ emissions within a single excitation cycle. Since the CW laser exclusively couples to the V-polarized transition, the single-photon purity of the $|BX\rangle \rightarrow |X_H\rangle$ photon remains unaffected. This limit can be overcome by further increasing the CW-laser detuning, selecting QDs with a smaller intrinsic FSS, or using other FSS compensation techniques.

IV. CALIBRATION OF PULSE AREA, OCCUPATION, AND ELECTRONIC WAVEFUNCTION RADIUS

To quantitatively compare the experimentally measured emission intensity with the simulated occupation, we calibrated the experimental TPE data by establishing the correspondence between the experimental observables and the theoretical parameters. As shown in Supplementary Fig. 4, the measured TPE emission intensity was fitted to the simulated occupation as a function of pulse area. In this fitting procedure, the conversion from measured photon counts to occupation and the conversion from the square root of the excitation power to pulse area were treated as free scaling parameters. In addition, the radius of the electronic wavefunction (a_e) was also included as a fitted parameter in the simulation model.

The fit yields a conversion factor of (0.0188 kcounts^{-1}) from the measured emission intensity to the occupation, a pulse-area calibration factor of (0.1832 $\pi/\mathrm{nW}^{0.5}$), and an electronic wavefunction radius of ($a_e = 5.5$ nm). These fitted parameters were then used in all relevant analyses throughout this work.

This calibration procedure relies on the following assumptions. First, within the TPE reference dataset, the integrated emission intensity is proportional to the final occupation under fixed collection and detection conditions. Second, the experimentally controlled pulse amplitude is related to the pulse area used in the simulation by a single global scaling factor. Third, once the calibration is established from the TPE dataset, the same normalization can be consistently applied to the FTPE measurements, allowing direct comparison between experiment and theory on the same scale.

We note that since phonon effects are not very pronounced at the moderate excitation powers used for this fitting, the extracted effective phonon-coupling parameters may be subject to some uncertainty, which could introduce a small error in the calibration.

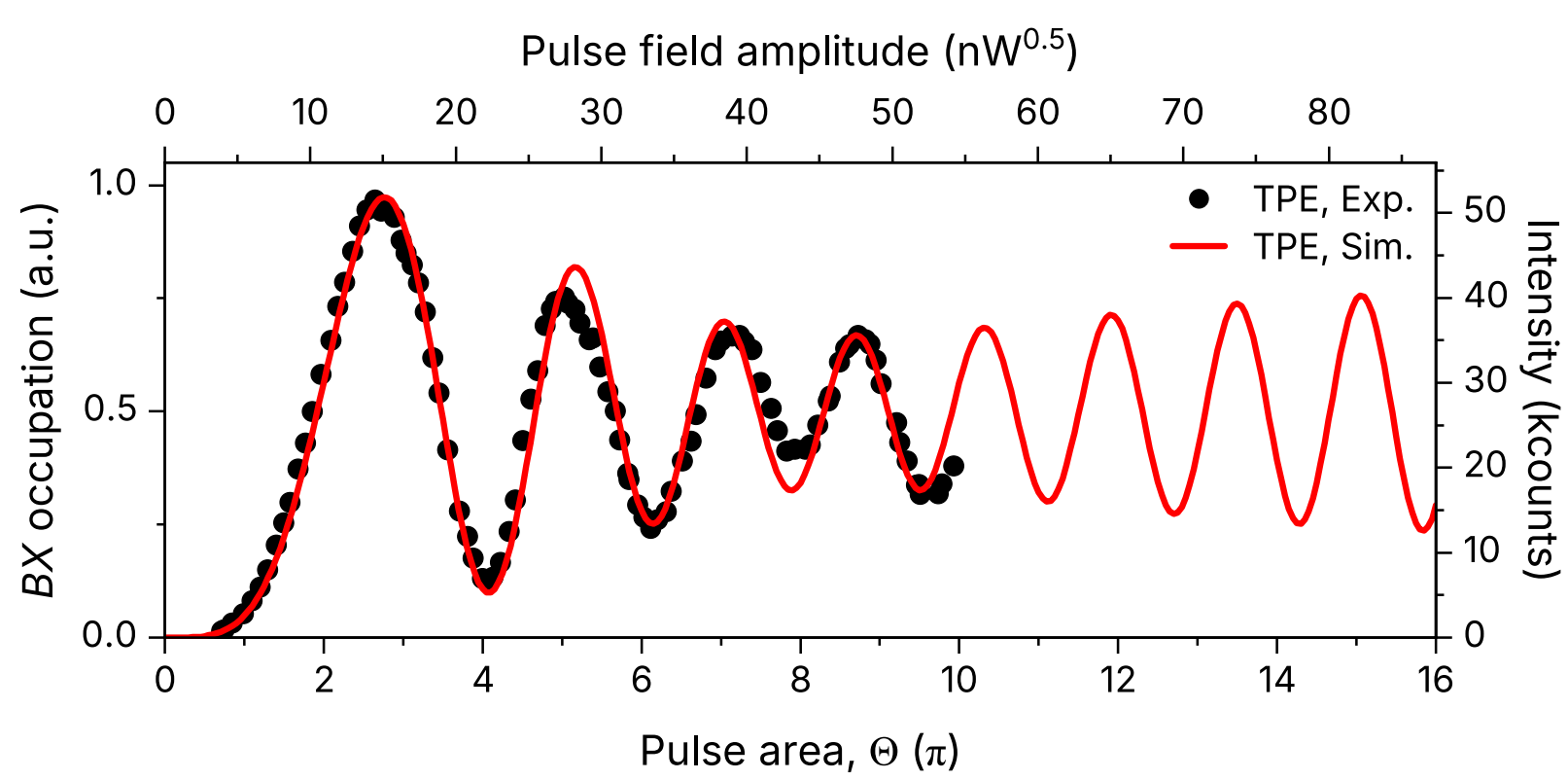

Supplementary Figure 4. **Calibration of the TPE Rabi oscillation.** Experimental TPE Rabi oscillation data are fitted to the simulated BX occupation as a function of pulse area. The fitting procedure determines the conversion factor from measured intensity to BX occupation, the calibration factor from pulse field amplitude to pulse area, and the radius of the electronic wavefunction.

V. PARAMETERS USED IN THE CALCULATIONS

The main parameters used in the calculations are summarized in Supplementary Table 1. These include the biexciton binding energy, the intrinsic fine-structure splitting, the effective electronic and hole radii, the pulse duration, the pulse detuning, and the detuning of the CW laser used for FSS compensation.

Unless otherwise specified, these parameters are fixed in the simulations presented in the main text and Supplementary Information.

Supplementary Table 1. Parameters used for the calculations.

Parameter	Symbol	Value
Biexciton binding energy	E_b	2.82 meV
Intrinsic fine-structure splitting	FSS	3.4 μeV
Electronic radius	a_e	5.5 nm
Hole radius	a_h	$a_e/1.15$
Pulse electric-field duration	τ_0	12.49 ps
Pulse detuning	$\hbar\delta$	3.75 meV
CW laser detuning	$\hbar\delta_{CW}$	300 μeV

VI. DETERMINATION OF SUITABLE DETUNING FOR FTPE IN GAAS QD

In principle, FTPE can operate over a broad range of laser detunings, as long as the two-photon resonance condition is satisfied and sufficient effective two-photon coupling is maintained. In the experiment, the practically accessible detuning range is limited by additional excitation channels in the weak-confinement GaAs/AlGaAs QDs system. To determine a suitable laser detuning for the FTPE measurements, we measured the X intensity under high-power single-pulse excitation as a function of detuning. As shown in Supplementary Fig. 5, for detunings smaller than about 3 meV, the X state can still be populated through LA-phonon-assisted excitation. At larger detunings than 4.5 meV, several resonant features appear, which we attribute to hole-state excitation [S3]. We therefore chose a detuning of 3.75 meV to demonstrate the reliability of the FTPE scheme as a compromise, where phonon-assisted excitation is largely suppressed while prominent hole-state excitation features are avoided.

The hole-state excitation could arise from structural asymmetry, strain-induced band mixing, and many-body configuration mixing [S4, S5], and is not included in our four-level theoretical model. This restriction is specific to the present material system rather than an intrinsic limitation of the FTPE scheme. For more strongly confined InAs/GaAs QD systems, the unwanted hole-state excitation is expected to be weaker, which should allow FTPE to be implemented at larger detunings and over a broader practical detuning window.

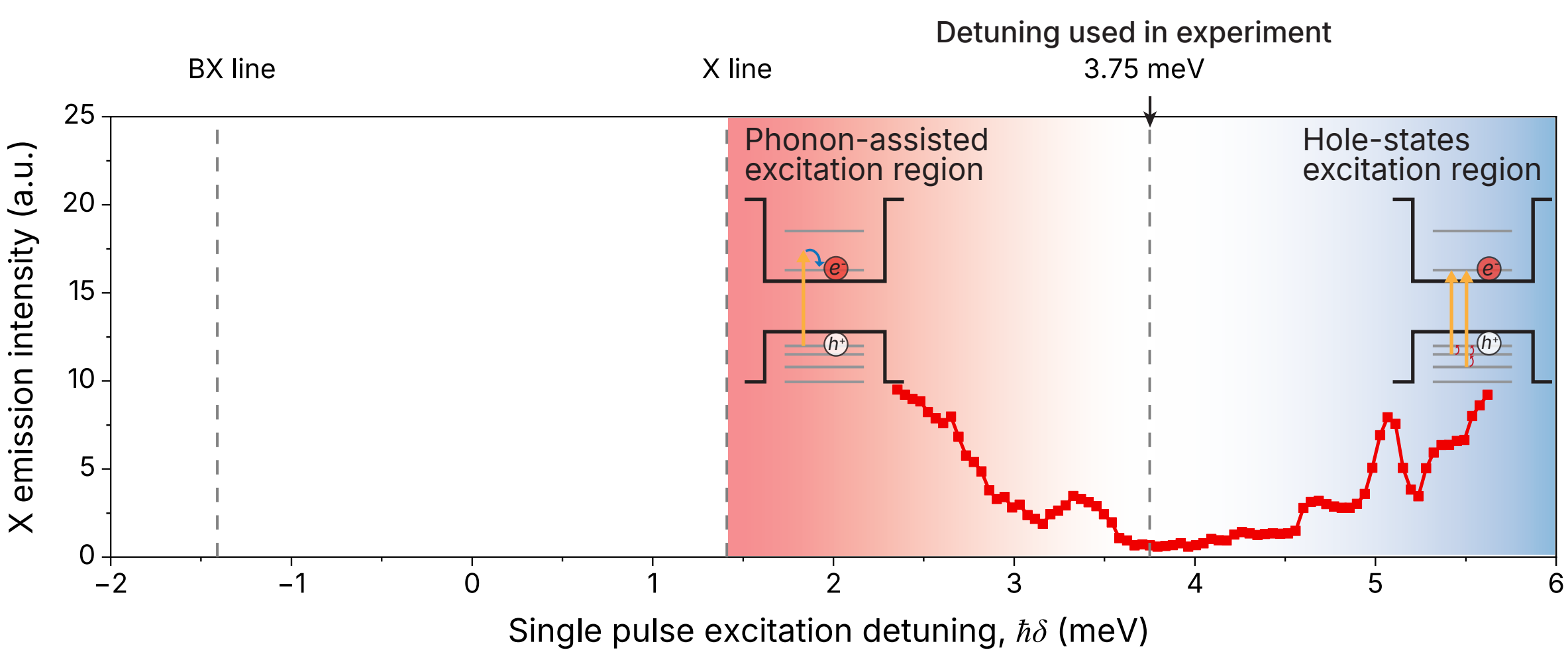

Supplementary Figure 5. **High-power monochromatic pulse excitation with different detunings.** For detunings smaller than ∼ 3 meV, the X state can be populated through LA-phonon-assisted processes, whereas at larger detunings, some population is created via hole-state excitation [S3].

VII. VALIDITY OF THE FLOQUET MODEL

Supplementary Fig. 6 shows the biexciton occupation for different levels of approximation used in our analytical investigation. In Supplementary Fig. 6 a) the full time dependence is accounted for, while a numerically calculated stroboscopic Floquet Hamiltonian is used for Supplementary Fig. 6 b). For Supplementary Fig. 6 c), the stroboscopic Hamiltonian was obtained using second-order Magnus expansion Eq. (7). For the detuning used in the experiment, which is denoted by the gray line, all three essentially match, confirming the validity of our approach.
Interestingly, the Floquet approximation, which assumes that the period $T = \frac{2\pi}{\delta}$ is much shorter than the time scales over which the laser envelopes change, is valid for almost the entire range of values shown in Supplementary Fig. 6. In contrast, the Magnus expansion, which expands the Floquet Hamiltonian in orders of $E_b/\hbar\delta$ and/or $\Omega_{1/2}/\delta$ breaks down for $\hbar\delta \simeq E_b$ and sufficiently large pulse areas Θ. The second-order expansion is sufficient to describe the first Rabi rotation, but does not reproduce higher pulse areas. Thus, if higher pulse areas or smaller binding energies are of interest, one would need to calculate the Floquet Hamiltonian numerically or use a higher order of the Magnus expansion.

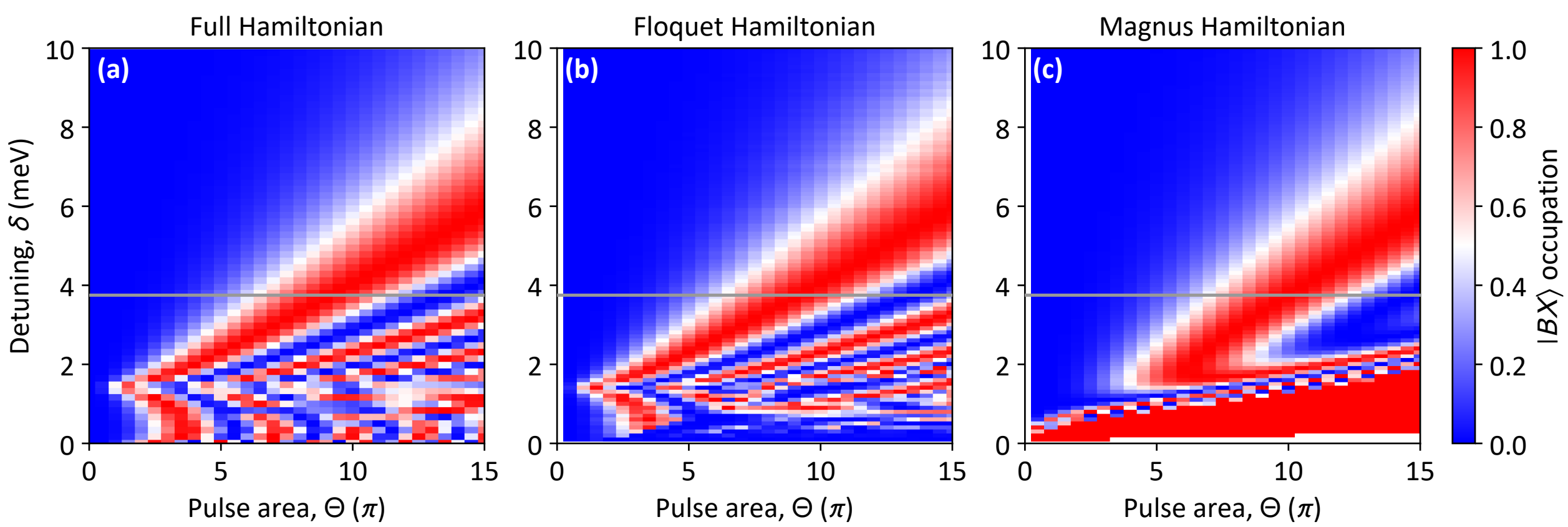

Supplementary Figure 6. Pulse-area and detuning dependence of the biexciton occupation for three different levels of approximation: (**a**) the full time-dependent Hamiltonian, (**b**) the results for the numerically calculated Floquet Hamiltonian and (**c**) the results for the second-order Magnus Hamiltonian in Eq. (7). All calculations are done with $\tau = 0$ ps and without phonons or losses. The gray line indicates the value of δ that is used in the experiment.

VIII. THEORETICAL ENTANGLEMENT FIDELITY

In this section, we simulate the entanglement fidelity of FTPE for $\tau = 0$ ps and $\tau = -2.9$ ps and compare the result with TPE. This is done for two cases: First, the FSS from the experiment is compensated for using a CW laser. In the second case, neither FSS nor CW laser is present. The resulting fidelities and density matrices in the polarization basis are shown in Supplementary Fig. 7. The simulated entanglement fidelities achieved by FTPE are close to those of TPE, though a small decrease of a fraction of a percent can be observed when using delayed pulses. Without FSS and the CW laser, the calculated fidelities are 98.2% for TPE, 98.0% for FTPE at $\tau = 0$ ps, and 97.7% for FTPE at $\tau = -2.9$ ps. The slightly lower fidelity of delayed FTPE arises from the longer effective driving duration and larger pulse areas, which lead to a slightly larger pulse-induced transient spectral shift.

When the FSS is compensated for by the CW laser, the fidelity generally decreases for all considered protocols. We attribute this to the laser-induced dressing of the QD states. In particular, the exciton gains a slight biexcitonic component, which enables transitions to the second exciton state, which decreases the entanglement fidelity. This shows that the experimentally obtained value for the fidelity may be increased considerably by using other mechanisms to compensate for the FSS.

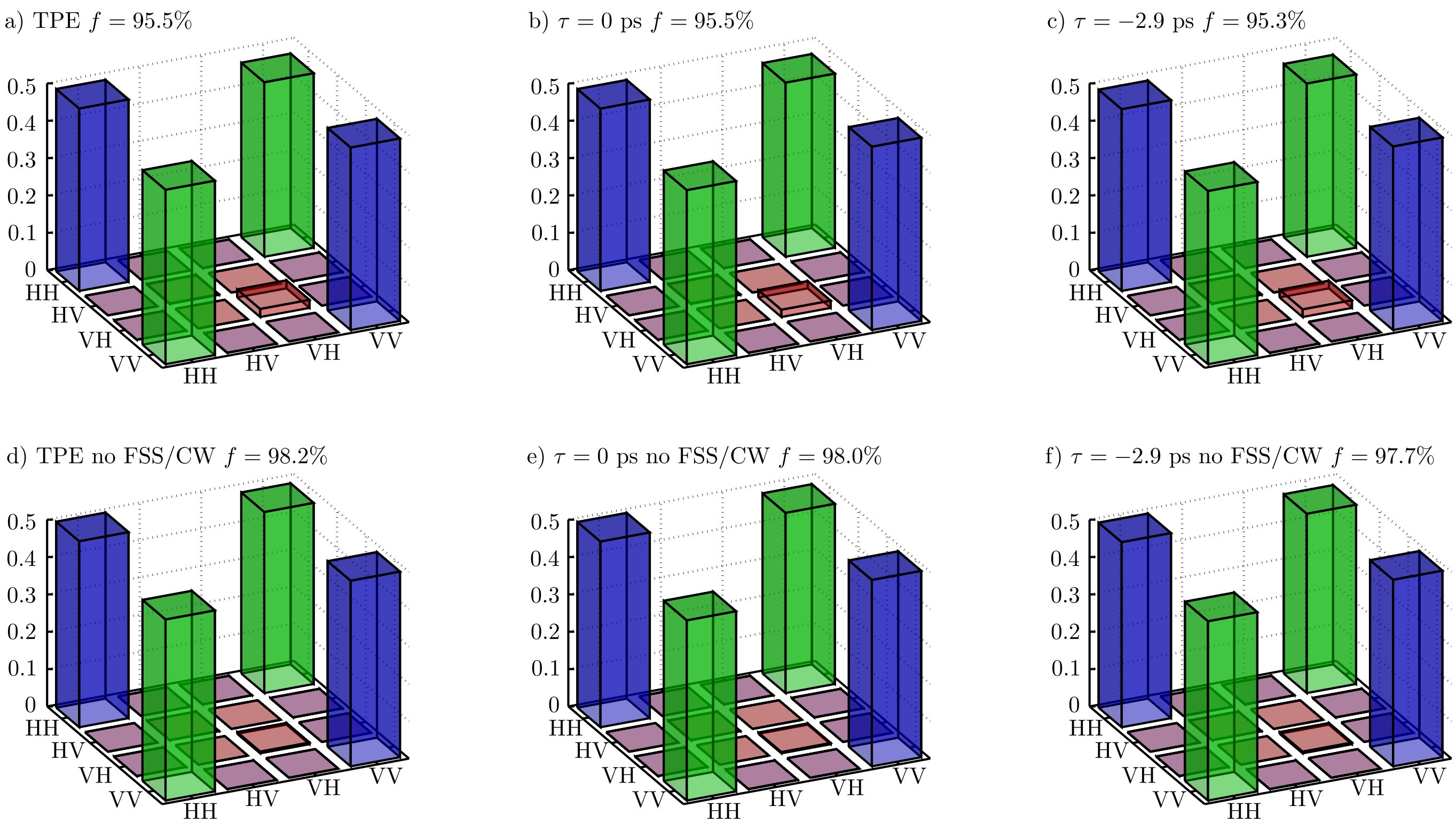

Supplementary Figure 7. The density matrices and entanglement fidelities for TPE and FTPE for $\tau = 0$ ps and -2.9 ps with the FSS compensated by a CW laser (**a-c**) and without any FSS (**d-f**). The calculations do not account for phonons.

IX. ROBUSTNESS OF FTPE AGAINST PARAMETER VARIATIONS

A. Influence of the pulse delay

As shown throughout this paper, a suitable choice of the delay τ allows for robust excitation. In Supplementary Fig. 8 we show the biexciton population as a function of pulse area and detuning for different delays. Qualitatively, we consider the excitation to be robust, when a large patch of parameters still produces high occupations. This leads to a trade-off between the demanded excitation fidelity and the size of the patch of parameters. In Supplementary Fig. 8 we see that for our experimentally used δ, which is displayed by the gray line, the excitation is robust against changes in the pulse area for delays roughly in the interval $[-2.9\text{ ps}, -3.5\text{ ps}]$. Supplementary Fig. 9 displays the excitation fidelity as a function of delay time and pulse area for different pulse durations τ_0. A longer pulse offers robustness at a larger pulse area.

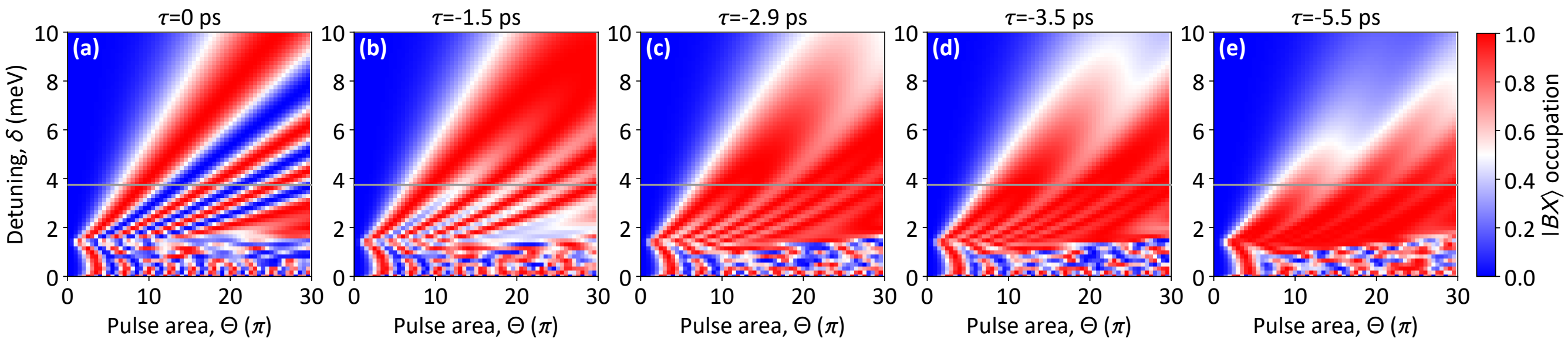

Supplementary Figure 8. The pulse area-detuning heatmaps of the biexciton occupation for five different delays $\tau = 0, -1.5, -2.9, -3.5$ ps and -5.5 ps. The gray line indicates the detuning δ used throughout this publication. The calculations do not account for phonons.

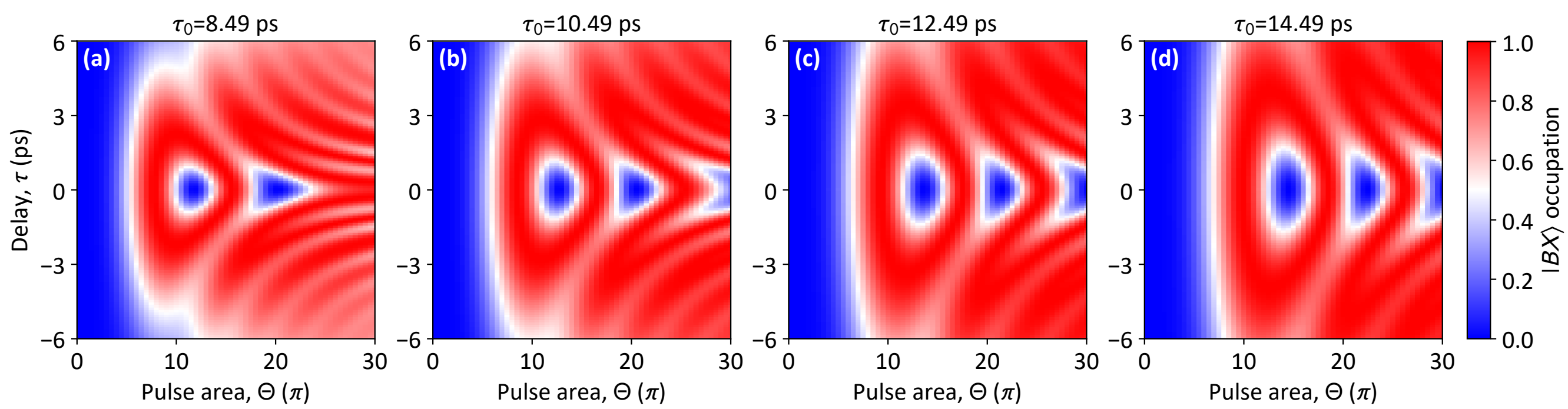

Supplementary Figure 9. The pulse area-delay heatmaps of the biexciton occupation for four different pulse durations $\tau_0 = 8.49, 10.49, 12.49$ ps and 14.49 ps. The calculations do not account for phonons.

B. Influence of spectral shifts

If charges near the QD shift the energy levels slightly, then the two-photon resonance that FTPE depends on can be broken. To investigate the severity of spectral wandering, we calculate the influence of such a detuning by shifting both lasers by a frequency δ_e. Biexciton population as a function of δ_e and the pulse area is shown in Supplementary Fig. 10. Without delay $\tau = 0$, detunings of $\delta_e \simeq 0.1$ ps^{-1} significantly reduce excitation efficiency. If, however, a finite delay, such as $\tau = -2.9$ ps, is used, the excitation becomes much more robust not just against changes in the pulse area, but also against deviations from the two-photon resonance.

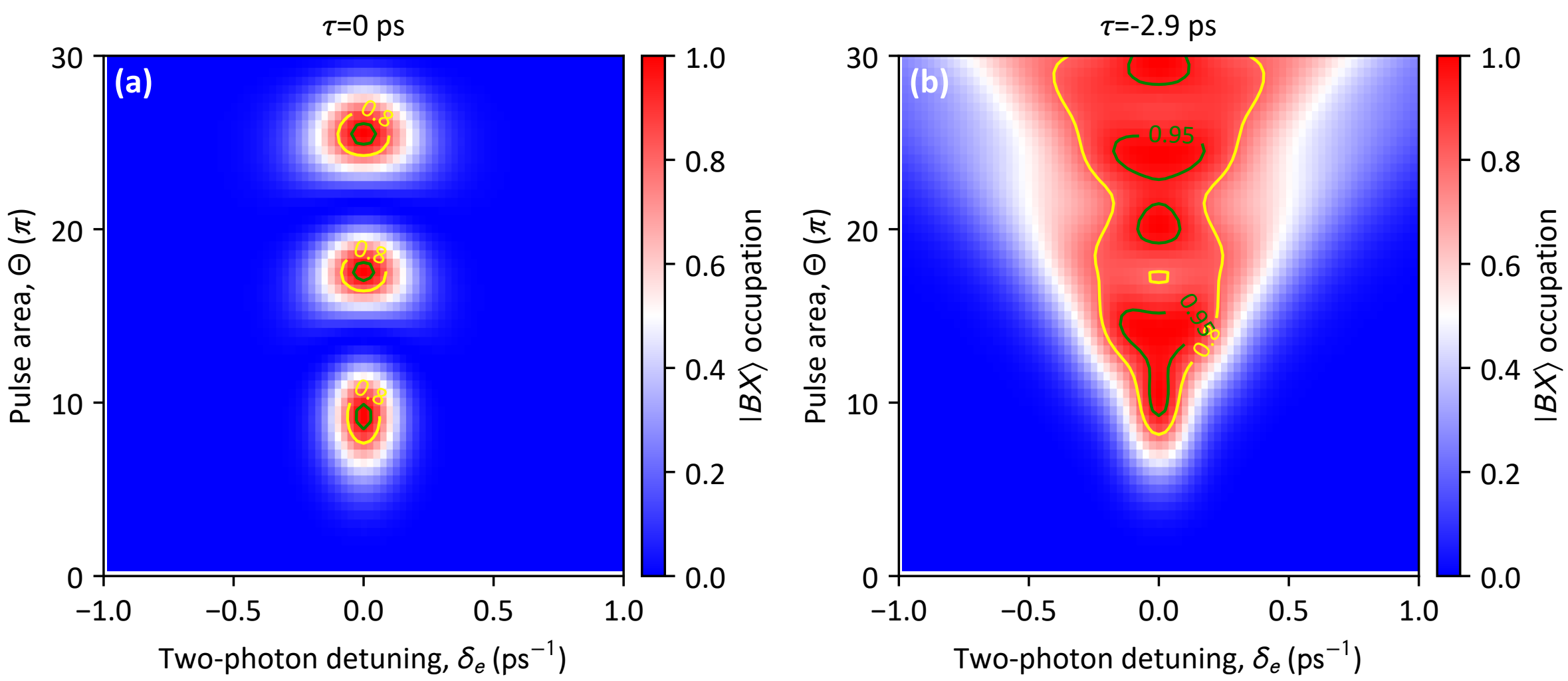

Supplementary Figure 10. Biexciton occupation for different pulse areas and detunings from the 2-photon resonance for $\tau = 0$ ps and $\tau = -2.9$ ps. These calculations were done without phonons or losses.

C. Influence of pulse-area imbalance

Equal peak intensities of the red- and blue-detuned pulses are not a strict requirement for FTPE. Nevertheless, equal driving strengths constitute the natural symmetric operating condition and are typically close to optimal. An imbalance between the two pulse amplitudes breaks this symmetry and modifies both the effective coupling and the associated stroboscopic evolution. Consequently, the optimal working point and the corresponding robustness window may shift, while high-fidelity biexciton preparation can still be achieved.

To quantify the tolerance of FTPE to such an imbalance, we independently vary the driving strengths of the red- and blue-detuned pulses. Supplementary Fig. 11 shows the resulting final biexciton population for simultaneous pulses, $\tau = 0$ ps, and for a finite delay, $\tau = -2.9$ ps. The calculations are performed without phonon coupling or radiative losses.

For $\tau = 0$ ps, the high-population region is relatively narrow under independent variations of the two pulse strengths. By contrast, for $\tau = -2.9$ ps, the region with high biexciton occupation extends over a substantially wider range of pulse-strength combinations. To quantify this difference more directly, Supplementary Fig. 11(c,d) shows line cuts through the corresponding maps at a fixed red-pulse area of $\Theta_r = 25\pi$. For $\tau = 0$ ps, the range of blue-pulse areas over which the biexciton occupation remains above 80% is only 5.5π. For $\tau = -2.9$ ps, this range increases to 29π.

These results show that the pulse-area robustness of FTPE is not restricted to simultaneous variations of both pulse strengths, but is also retained when the two pulse areas vary independently. Equal peak intensities should therefore be regarded as a convenient near-optimal operating condition rather than a fundamental requirement of FTPE.

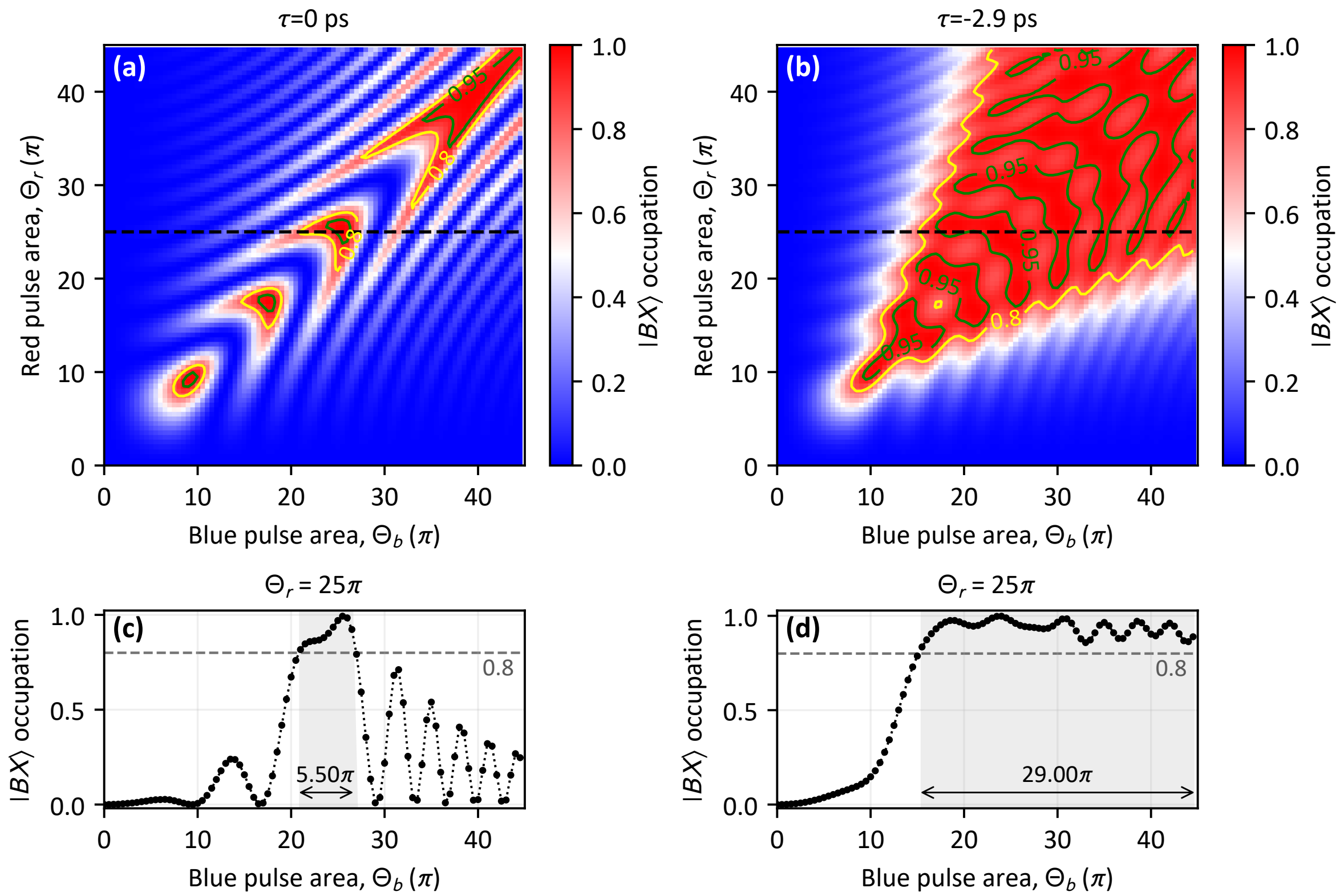

Supplementary Figure 11. Final biexciton population after FTPE as a function of the independently varied driving strengths of the red- and blue-detuned pulses for $\tau = 0$ ps (**a**) and $\tau = -2.9$ ps (**b**). The calculations were done without phonons or losses. Contour lines for the biexciton occupation 80% and 95% are shown using yellow and green lines, respectively. (**c,d**) Line cuts of (**a,b**), respectively, at a fixed red-pulse area of $\Theta_r = 25\pi$, showing the biexciton occupation as a function of the blue-pulse area. The shaded regions indicate the ranges of blue-pulse area over which the biexciton occupation exceeds 80%.

X. COMPARISON WITH OTHER EXCITATION MECHANISMS

A. Nearly monochromatic two-photon resonant excitation

While conventional monochromatic two-photon excitation is described by the same Hamiltonian as in Eq. (1) in the limit of zero detuning $\delta \to 0$, it is important to note that FTPE and TPE are qualitatively different and appear in opposite parameter regimes. It is instructive to consider the prediction of the usual description of TPE [S6] when a finite detuning δ is accounted for. Here, we focus on the case of zero delay $\tau = 0$ between the pulses, where the driving is given by

$$\Omega(t) = 2f(t)\cos(\delta t). \tag{S2}$$

In contrast to the description of FTPE valid for large detunings, TPE is described using an adiabatic approximation [S6]. There the initial state $|G\rangle$ is decomposed into laser-dressed eigenstates. Over the pulse duration, the occupation of the instantaneous eigenstates is assumed to be constant by the adiabatic theorem, while the respective components of the wave function acquire dynamical phases corresponding to the time integral over the respective energy eigenvalues. The final biexciton state occupation $|\langle BX|\Psi\rangle|^2$ is then the result of the interference of the different eigenstate components of the wave function. One finds [S6]

$$|\langle BX|\psi\rangle|^2 = \sin^2\frac{\Lambda}{2}, \tag{S3}$$

with

$$\Lambda = \frac{1}{4\hbar}\int_{-\infty}^{\infty}\left[E_b - \sqrt{E_b^2 + 4\cdot 8\hbar^2\Omega^2(t)}\right]\mathrm{d}t. \tag{S4}$$

The final biexciton state occupations are depicted in Supplementary Fig. 12 for both direct numerical simulations of the dynamics and the results of the adiabatic approximation. Indeed, for small detunings $\delta \ll E_b/(2\hbar)$, the adiabatic approximation captures the Rabi-like behaviour of the full dynamics well. However, for larger detunings, here $\delta \gtrsim E_b/(4\hbar)$, the adiabatic approximation starts to break down and the distribution of parameter sets for which a significant biexciton population is achieved becomes erratic.

Clearly, at $\delta = E_b/(2\hbar)$ the lasers are resonant with direct transitions involving the single exciton state. Thus, even though near TPE and FTPE have in common that they involve virtual transitions without significant occupation of the exciton state, they are clearly distinguished by the parameter regimes $\delta < E_b/(2\hbar)$ and $\delta > E_b/(2\hbar)$. Thus, the physics in both regimes is different, which is why a different theoretical approach is required to explain FTPE.

B. Adiabatic protocols

A number of state preparation protocols for quantum dots have been proposed that rely on adiabatic driving, such as adiabatic rapid passage using a chirped laser pulse [S7], whose frequency changes over time, or ultrafast electric tuning [S8]. Also phonon-assisted state preparation employs adiabatic undressing [S9] as a working principle. In particular, adiabatic protocols with the state of the systems confined to the lowest instantaneous eigenstate for most of the evolution are robust against small variations, e.g., of the pulse area. This raises the question of whether the robustness found for FTPE is related to adiabatic transitions.

To this end, we depict in Supplementary Fig. 13 the occupations of instantaneous eigenstates of the stroboscopic Hamiltonian for parameters $\hbar\delta = 3.75$ meV and $E_b/2 = 1.41$ meV for cases without ($\tau = 0$ ps) and with ($\tau = -1.5$ ps) a delay between the pulses for FTPE. For finite delay, it can be seen that the dynamics is clearly nonadiabatic in the instantaneous eigenbasis of the stroboscopic Hamiltonian. Nevertheless, the dynamics is found to be robust. For vanishing delay, the occupations of instantaneous eigenstates remain nearly constant, similar to the behaviour expected in the adiabatic limit. But because the system is in a coherent superposition between two eigenstates, the relative phase is generally sensitive to variations in the pulse area, making the protocol not more robust than, e.g., Rabi driving. To summarize, in the Floquet regime, the dynamics may or may not be adiabatic, depending on the pulse delay, and the enhanced robustness is mainly found in the diabatic regime. This shows that the physics of FTPE differs from that of conventional adiabatic protocols.

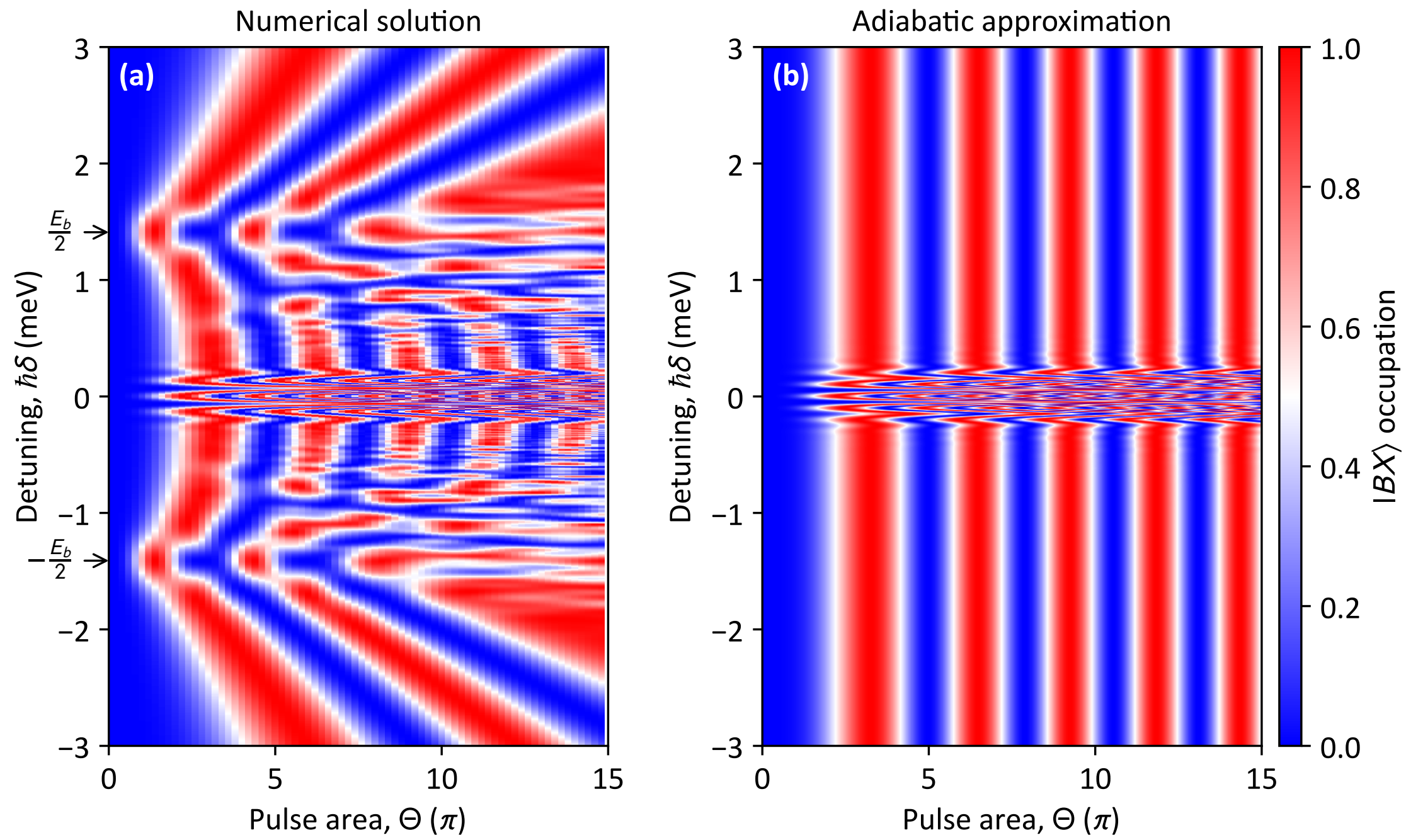

Supplementary Figure 12. Numerically calculated final biexciton population under near-two-photon resonant excitation as a function of detuning δ and pulse area per pulse Θ obtained by (**a**) direct numerical simulation of the three-level dynamics without phonons or losses and (**b**) using the adiabatic approximation Eq. (S3). Half the biexciton binding energy is chosen as $E_b/2 = 1.41$ meV and quantum dot-phonon interactions are neglected in both calculations.

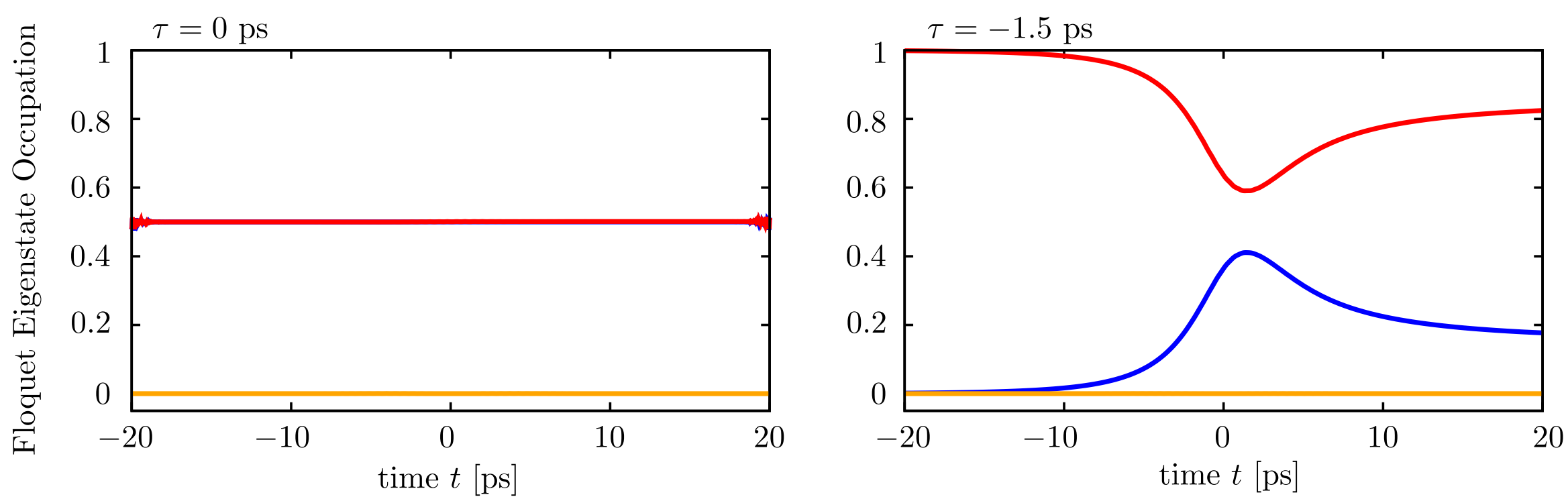

Supplementary Figure 13. Time evolution of the occupation of all three eigenstates of the stroboscopic Hamiltonian $\bar{H}$. Displayed are cases without any delay, $\tau = 0$ ps, as well as a small negative delay, $\tau = -1.5$ ps. Adiabatic transitions require occupations of instantaneous eigenstates to be constant in time, which is not fulfilled for finite delays.

C. STIRAP

A state preparation scheme whose setup, on first glance, resembles FTPE is stimulated Raman adiabatic passage (STIRAP) [S10]. STIRAP is designed to drive transitions between two ground states via a third, intermediate state. One assumes selection rules where the first (pump) pulse couples only the first ground state to the intermediate state and the second (Stokes) pulse couples the intermediate state to the second ground state. Furthermore, both lasers are detuned from the respective transition with the same detuning Δ. In the basis of the second ground, intermediate, and first ground states, the corresponding Hamiltonian is given by

$$H^{\text{STIRAP}} = \hbar \begin{pmatrix} 0 & \frac{\Omega_p}{2} & 0 \\ \frac{\Omega_p}{2} & \Delta & \frac{\Omega_s}{2} \\ 0 & \frac{\Omega_s}{2} & 0 \end{pmatrix}. \tag{S5}$$

where $\Omega_p(t)$ and $\Omega_s(t)$ are the (real) amplitudes of the pump and Stokes pulses, respectively. The Hamiltonian is similar to Eq. (1) when the detunings are identified by $\Delta = \delta - E_b/(2\hbar)$. The delay and pulse area dependence of both STIRAP and FTPE for these parameters are depicted in Supplementary Fig. 14. Both show Rabi rotations for zero delay and broad parameter regimes with large final state occupations.

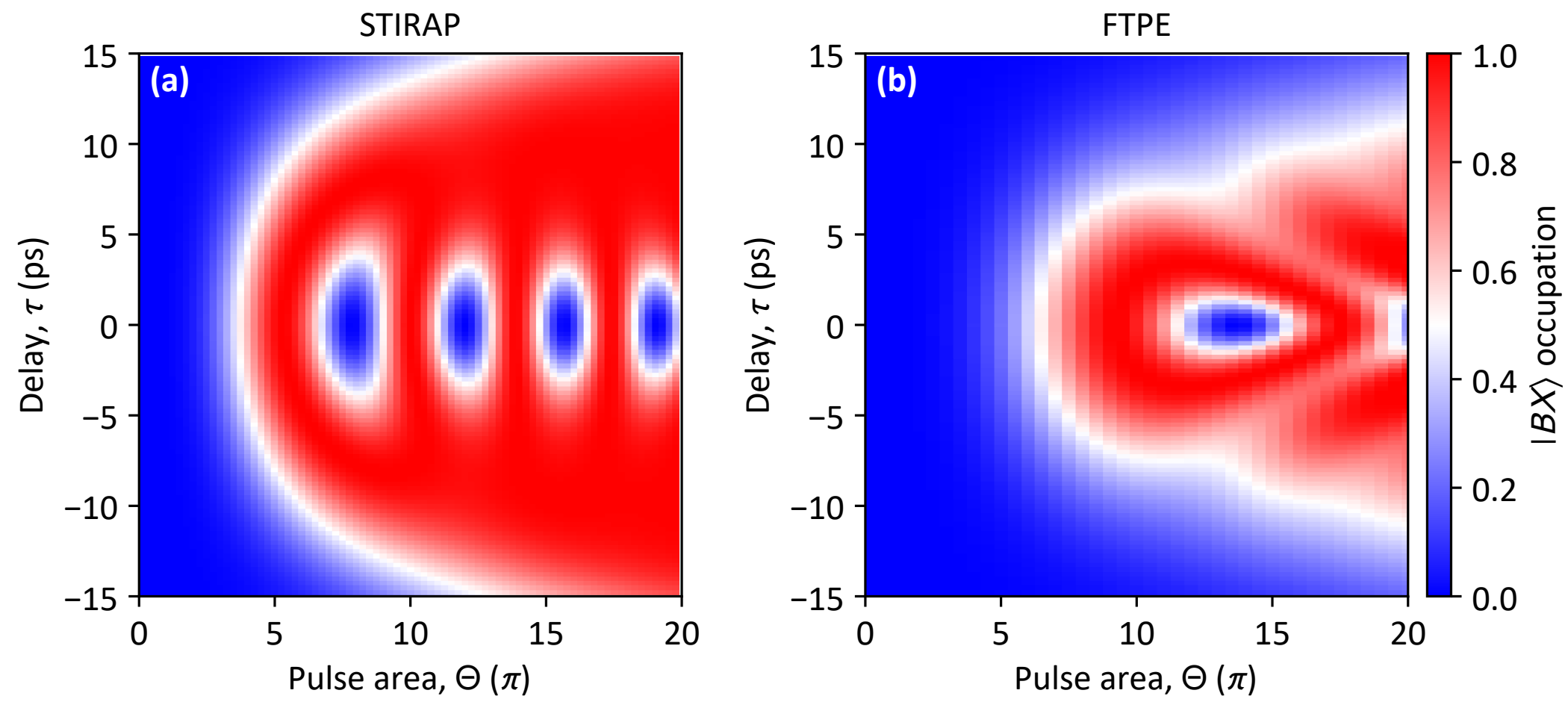

Supplementary Figure 14. Comparison of the τ-Θ dependence of the biexciton occupation for regular STIRAP and FTPE. Θ is the pulse area of either individual laser. The detuning to the central level was chosen $\hbar\Delta = 2.34$ meV in both cases. The calculations were done without phonons or losses.

However, there are also clear differences between STIRAP and FTPE. Most importantly, for STIRAP, the transitions can be controlled separately by the two laser pulses. The optical selection rules in the biexciton-exciton system of a quantum dot, on the other hand, are such that linearly polarized lasers couple both transitions simultaneously by both pulses. Even in the case of two lasers with oppositely circularly polarized pulses, for which the first pulse couples the ground state to an intermediate exciton state in the circular polarization basis and the second pulse couples the same exciton state to the biexciton state, the situation deviates from STIRAP, since for finite overlap of the pulses transitions to the exciton state with opposite polarization (a fourth level) are possible. Either way, the optical selection rules for semiconductor quantum dots prohibit the straightforward implementation of STIRAP for biexciton state preparation.

D. SUPER and dichromatic excitation

Recently, two-colour excitation schemes have been explored for state preparation of quantum dots, in particular, exciton state preparation for applications in on-demand single-photon generation [S11, S12]. An important goal of off-resonant excitation is that photons from the excitation laser can be easily spectrally separated from the emitted quantum state of light. A detailed analysis of the Swing-UP of quantum Emitter Population (SUPER) scheme has revealed that parameters for which off-resonant two-colour excitation leads to near-unity population inversion are found when one laser becomes resonant with the transition between the laser-dressed states Stark-shifted by the other laser [S13].

FTPE has in common with SUPER mainly the fact that two-colour off-resonant excitation is employed. But in contrast to SUPER, Stark shifts do not set the resonance condition or constitute the working mechanism of FTPE. For the same reason, FTPE also differs from dichromatic excitation with one red- and one blue-detuned laser [S12]. In Ref. [S14], the use of SUPER for the excitation of the biexciton state was investigated. Starting with the same parameters as in Ref. [S14], we vary both pulse areas and depict the resulting biexciton population in Supplementary Fig. 15 for three different pulse delays. Compared to the corresponding results for FTPE, which are displayed in Supplementary Fig. 11(b), SUPER is more sensitive to variations in the resonance conditions, as can be seen by the fact that the area enclosed in the 80% and the 95% is considerably larger for FTPE.

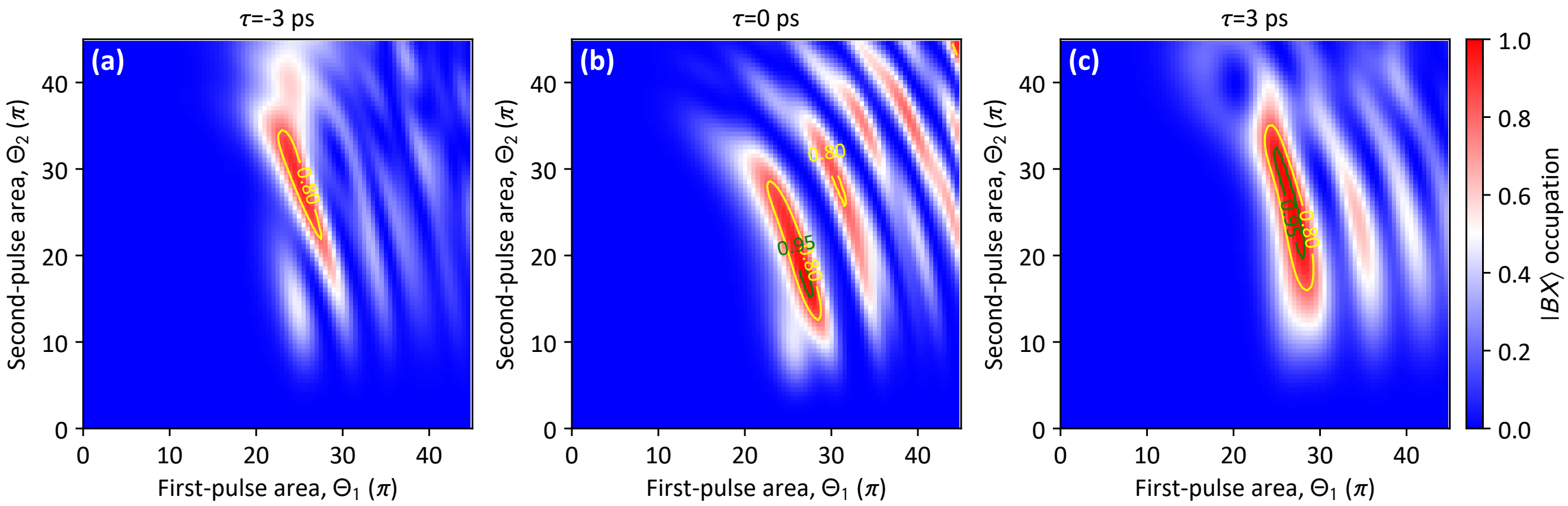

Supplementary Figure 15. Biexciton occupation of SUPER excitation using parameters from Ref. [S14] for different pulse areas and delays. The calculations were done without phonons or losses.

XI. RELATIVE PHASE DIFFERENCE BETWEEN THE LASERS

It is possible to add a relative phase ϕ to one of the lasers. Then, the laser function reads

$$\Omega(t) = f(t - \tau/2)\mathrm{e}^{-\mathrm{i}\delta(t-\tau/2)} + f(t + \tau/2)\mathrm{e}^{\mathrm{i}(\delta(t+\tau/2)+\phi)}. \tag{S6}$$

The numerical investigations in Supplementary Fig. 16(a) show that the relative phase between the lasers is irrelevant for the final occupation. Our protocol produces the same excitation regardless of the specific value of ϕ. It is similar to SUPER in this aspect. But, again like for SUPER, the oscillations during the excitation are affected, as can be seen in Supplementary Fig. 16(b).

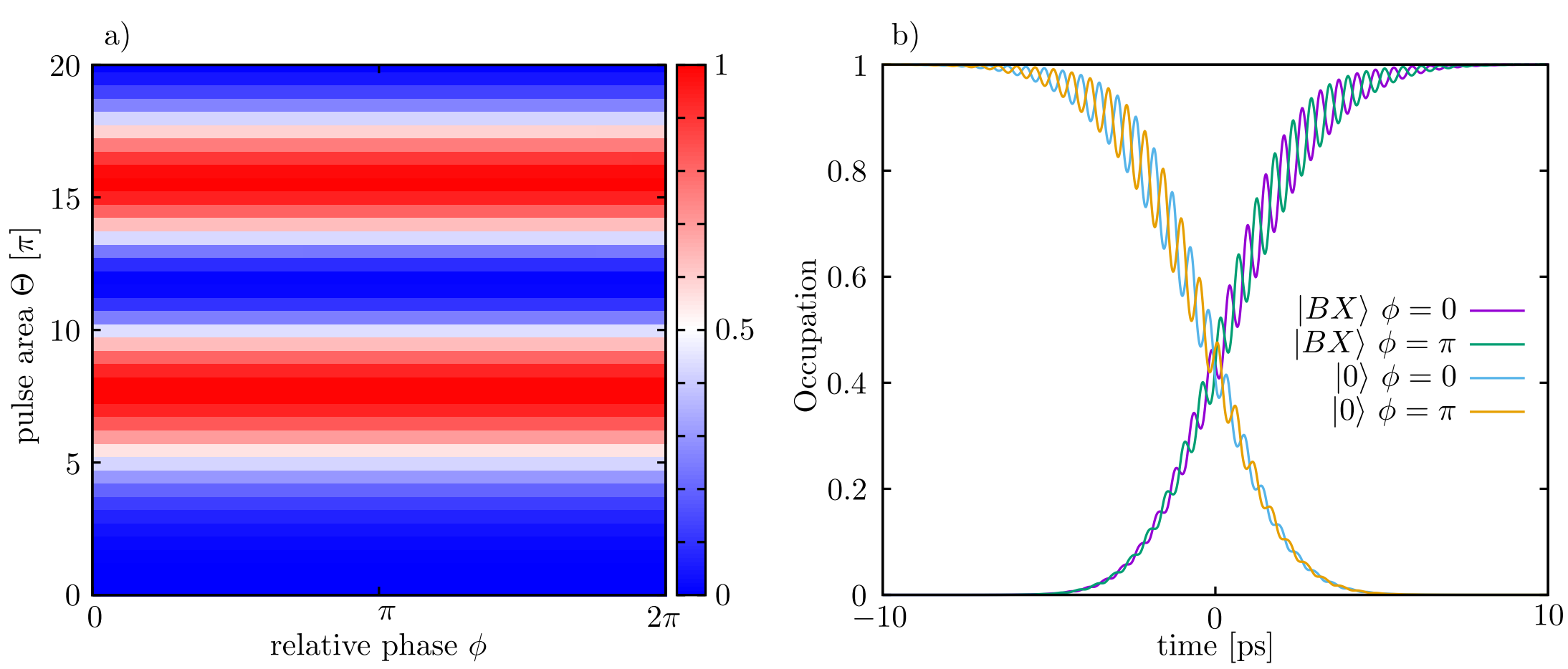

Supplementary Figure 16. **(a)**, Biexciton occupation after using two pulses with pulse area Θ in which one has the relative phase ϕ, as in Eq. (S6). $\hbar\delta = 3.75$ meV was chosen as a typical value and no delay between the lasers was used. The occupation clearly does not depend on the relative phase between the pulses. We find that the same applies in cases of $\tau \neq 0$ (not shown). **(b)**, The occupation of ground and biexciton states for two exemplary phases $\phi \in \{0, \pi\}$.

[S1] Zhou, L. *et al.* Polarization Calibration Scheme for a Practical Handheld Free Space Quantum Key Distribution Link. In *2019 IEEE Globecom Workshops (GC Wkshps)*, 1–5 (IEEE, 2019).
[S2] Shao-Kai, W., Ji-Gang, R., Cheng-Zhi, P., Shuo, J. & Xiang-Bin, W. Realization of Arbitrary Inverse Unitary Transformation of Single Mode Fibre by Using Three Wave Plates. *Chinese Physics Letters* **24**, 2471–2474 (2007).
[S3] Reindl, M. *et al.* Highly indistinguishable single photons from incoherently excited quantum dots. *Physical Review B* **100**, 155420 (2019).
[S4] Narvaez, G. A. & Zunger, A. Nominally forbidden transitions in the interband optical spectrum of quantum dots. *Physical Review B* **74**, 045316 (2006).
[S5] Holtkemper, M., Reiter, D. E. & Kuhn, T. Influence of the quantum dot geometry on p-shell transitions in differently charged quantum dots. *Physical Review B* **97**, 075308 (2018).
[S6] Stufler, S. *et al.* Two-photon Rabi oscillations in a single Inx Ga1-x As GaAs quantum dot. *Physical Review B* **73**, 125304 (2006).
[S7] Gawarecki, K. *et al.* Adiabatic rapid passage in quantum dots: phonon-assisted decoherence and biexciton generation. *physica status solidi c* **10**, 1210–1213 (2013).
[S8] Mukherjee, A. *et al.* Electrically controlled rapid adiabatic passage in a single quantum dot. *Applied Physics Letters* **116** (2020).
[S9] Barth, A. M. *et al.* Fast and selective phonon-assisted state preparation of a quantum dot by adiabatic undressing. *Phys. Rev. B* **94**, 045306 (2016).
[S10] Bergmann, K., Vitanov, N. V. & Shore, B. W. Perspective: Stimulated Raman adiabatic passage: The status after 25 years. *The Journal of Chemical Physics* **142**, 170901 (2015).
[S11] He, Y.-M. *et al.* Coherently driving a single quantum two-level system with dichromatic laser pulses. *Nature Physics* **15**, 941–946 (2019).
[S12] Koong, Z. X. *et al.* Coherent dynamics in quantum emitters under dichromatic excitation. *Phys. Rev. Lett.* **126**, 047403 (2021).
[S13] Bracht, T. K. *et al.* Swing-up of quantum emitter population using detuned pulses. *PRX Quantum* **2**, 040354 (2021).
[S14] Bracht, T. K. *et al.* Dressed-state analysis of two-color excitation schemes. *Physical Review B* **107**, 035425 (2023).
