# Supplementary material for: Robust biexciton preparation for entangled photon-pair generation by single-shot Floquet driving
Source: Nat Commun. 2026 Sep 12;17:9745. doi: 10.1038/s41467-026-77221-9 (PMC13570888; doi:10.1038/s41467-026-77221-9)
Supplement: Supplementary file 2 [file 41467_2026_77221_MOESM2_ESM.pdf]

nature portfolio

Peer Review File

Robust biexciton preparation for entangled photon-pair generation by single-shot Floquet driving

Corresponding Author: Professor Feng Liu

This file contains all reviewer reports in order by version, followed by all author rebuttals in order by version.

Version 0:

Reviewer comments:

Reviewer #1

(Remarks to the Author)
The manuscript Robust entangled photon generation enabled by single-shot Floquet driving by Yan et al. is investigating a dichromatic scheme to excite the biexciton state resonantly.
I do find the manuscript well written and it adresses a timely topic. The presented results are also backed up by a sound theory and the data is analysed and presented well. However, I do have quite some comments, where I don't understand stuff or things I think should be addressed.

Firstly, while I am not an expert on Floquet physics, I am not sure that the presented physics has anything to do with Floquet theory. In Floquet theory (as far as I know, as a non-expert), the drive system is continuous and periodic. This is not the case in a pulsed scheme. That two lasers at different frequencies create a beating amplitude is not too unexpected an I am not sure it is a Floquet drive.
Furthermore, I also don't see the difference to the SUPER scheme, which does exactly the same. The only difference seems to be that in the SUPER scheme, both pulses are detuned red while here one pulse is detuned red and one is detuned blue. The super scheme is dealing with a two level system, thus the analyis of the excitation of the biexciton is interesting, but the SUPER scheme also uses beating lasers and there it is not a Floquet drive (which I believe is correct - that it is not).
That a dicromatic excitation with two photons will be able to excite the biexciton state is not too unexpected. Momentum and energy are conserved as long as it is a 2-photon process, as the authors mention. Dichromatic exitaiton of a single line, as e.g. in https://journals.aps.org/prl/abstract/10.1103/PhysRevLett.126.047403, with one of the coautors as coautor, is much more unintuitive, as you can't just absorb one photon from each color as this would break momentum as well as energy conservation.

Besides this very fundamental concern about the title and the use of the word Floquet drive, I do have some more comments on the actual content of the paper:
- in eq. 1: what is E_b? I assume it is the detuning of the laser to the Exciton level, but it is nowhere described.

- I don't understand how the authors arrive at Eq. 2. Furhtermore, I do not understand if the hamiltonian is now an effective magnetic field hamiltonian, where I don't know why electric fields can be neglected or if the magnetic field is just an anology. Maybe this could be discussed better.

- As a positive comment: I do really like the explanation of the popolation transfer in Fig. 2 and the corresponding part in the main text. I think this gives some understanding of what is happening instead of just presenting the outcome.

- I wonder why in figure 3 the lowest pulse area to achieve high BX occupation is at around 1.7 meV detuning. This seems to be even lower pulse area than for the resonant TPE case with delta=0.
I further wonder how the pulse area is normalized, as I would expect system inversion in the resonant TPE case for a Pi pulse. In the presented results this seems to be at about 4pi.

- In Fig. 3 c, the experimental state preparation for FTPE seems to be significantly higher than the TPE, while in the calculation of Fig. 3 b, they seem to reach similar values. Where does this discrepancy come from?

- In the main text the authors claim 98.7 % preparation efficiency for the plot in Fig. 3a. From the figure itself it seems like the

value is not much higher than 0.9, but this is because it is very hard to tell how dark red the red actually is. I suggest to maybe plot another contour level at 95% (or even higher).

- For measuring the data of fig 3c and fig 4, I wonder why 3.75 meV detuning was chosen. It seems from Fig 3a that the optimal detuning for maximum BX excitation would be at about 5 meV. The authors report to measure 96% BX occupation at 37.7 µW, -1.5 ps and 3.75 meV detuning. From Fig 3a it seems that the theoretical maximum for this detuning and timing is at about 92%. How can the experimental value be over the theoretical achievable one? I further wonder, how the authors experimentally verify the occupation probability. It would benefit the paper a lot if they can elaborate on that. Often the experimental data is compared to a theoretical fit to determine the inversion efficiency, but here it seems that that number was found independent, as it is comparing experiment to theory.

- The authors find -1.5 ps to be the optimal delay. I wonder how this changes with excitation pulse length and detuning, as these were fixed parameters.

- On the entanglement measurement:
if the authors apply such strong power to drive the system, I wonder how this affects the AC stark shift of the driving laser itself, as found to be detrimental for entanglement generation. (https://doi.org/10.1103/PhysRevLett.131.166901). I don't know if the authors have enough counts and time resolution to look at this effect in the time domain, but it should also be observable in the spectrum as a shoulder on the biexciton line. Maybe having 2 lasers with red and blue detuning to the exciton is partly cancelling the AC stark shift of the exciton state. It would be very interesting to see whether the proposed scheme also works in the case where the laser that is less detuned with respect to the exciton is much weaker, such that the two laser's AC stark shifts cancel each other out. Maybe this could be investigated in theory?
I further wonder how far detuned the additional, quite strong cw laser is, that the authors use for the compensation of the FSS. And I wonder whether this laser interacts somehow with the exitation pulses for additional exitation mechanisms, as it will beat with the applied pulsed laser as well. Is this taken into account into the presented theory?
Addressing the AC stark shift on the entanglement is one of the major missing pieces in the manuscript, as the proposed scheme needs quite a lot of power and if it therefore introduces a large AC stark shift, it would be hindering the creation of high fidelity entanglement. This becomes even more an issue if dealing with high-performance sources such as QDs in Purcell enhanced microcavities, as when the lifetime is significantly shortened, the portion of the light that experiences the stark shift is becoming larger. A scheme that could compensate for that would be of utmost interest to the community! I think this has to be properly discussed to evaluate the real strengths and weaknesses of the proposed scheme.

I think if the above points are addressed properly, the manuscript should be published as it shows very interesting physics.

With best regards,
Prof. Dr. Tobias Huber-Loyola

Reviewer #2

(Remarks to the Author)
The manuscript describes the theory and experiment addressing a very important problem in photonic quantum information technology: the generation of robust, on-demand, high-fidelity entangled photon pairs. Although this is a well-studied topic, the manuscript introduces a genuinely new perspective to the field and merits publication in Nature Communications. The material is well presented, easy to follow, and both the experimental and theoretical methods and data appear convincing. The material is also well illustrated.
I have two minor points concerning readability:
-T=2\pi / delta should be defined upon the first mention of T, for instance, at the beginning of the section "Theory on Floquet-engineered TPE," as this clarifies its physical meaning.
-On page 3, after Eq. 2, the physical meaning of \(\sigma _{x}\) and \(\sigma _{z}\) should be properly introduced (even if known to most readers).
I also have three points—important in my view—that should be addressed before the paper is published:
a) Given the vast amount of work on entanglement in quantum dot emission (which uses similar material compositions), I—and I assume many readers not directly involved in this specific field of research—would appreciate a comparison of fidelity, preparation efficiency, and other metrics from different works/methods. Including experimental and theoretical figures in the "Discussion and Conclusion" section would help us judge the superiority of this work, as many proposals exist, also explained in the supplement.
b) Can the authors comment on the influence of zero-phonon line broadening on the scheme? This is often a critical aspect that differs from one dot to another.
c) B_{x} and B_{z} should be connected to an explicit equation in the appendix. Additionally, would it not be more reasonable to connect \(B_{x}\) to an effective optical field?

All in all, this is nice work and the authors will be able to address these question in a convincing way.

Reviewer #3

(Remarks to the Author)

This manuscript reports a novel excitation protocol—single-shot Floquet-engineered two-photon excitation (FTPE)—for

robust biexciton preparation in a semiconductor quantum dot. By using two symmetrically detuned ultrafast pulses, the authors approximate an effective stroboscopic Hamiltonian that directly couples the ground and biexciton states. Both theoretical modeling including phonon interactions and convincing experimental demonstrations are presented, achieving biexciton preparation efficiencies exceeding 96% and polarization-entanglement fidelities of 93.4%. The work addresses challenges in quantum-dot–based entangled photon sources. The proposed approach is original, and experimentally executed. Overall, the manuscript is well within the scope of Nature Communications and represents a significant advance in quantum photonics and coherent control.

That said, there are several important points that require clarification, strengthening, or deeper discussion, particularly regarding the theoretical assumptions, the generality of the protocol, and comparison with existing schemes.

1. The effective Hamiltonian relies on a higher order expansion in the 1/ delta, assuming detuning delta large. While this condition appears satisfied for the chosen parameters, the manuscript would benefit from a more explicit quantitative discussion of the regime of validity, especially since second- and third-order terms are said to be relevant. For example authors can clarify how close one can approach with smaller detunings before the effective two-level description breaks down.

2. Although the exciton is claimed to remain only weakly populated, it still plays a crucial virtual role. The Schrieffer–Wolff elimination assumes a strong separation of energy scales. Include a quantitative bound (maximum transient exciton occupation) across the parameter space explored experimentally. Discuss whether residual exciton population could contribute to dephasing or timing jitter in other regimes.

3. The manuscript states that FTPE is distinct from, phonon-assisted excitation, STIRAP, and SUPER, but the comparison remains largely qualitative. Clarifying explicitly in what sense FTPE offers a unique advantage rather than an incremental improvement, will be helpful.

4. Authors claim that the fidelity is limited by re-excitation induced by the CW ac-Stark laser used to cancel FSS, rather than by FTPE itself. It weakens the claim of intrinsic robustness. A comment on whether FTPE could be combined with alternative FSS-removal techniques (e.g., strain or magnetic fields) and an estimate of the achievable fidelity in the absence of CW-induced re-excitation, should be added.

5. The conclusion suggests applicability to molecules, atoms, color centers, and superconducting qubits, but no concrete examples are discussed. Briefly outline what physical requirements (level structure, selection rules, achievable detunings) are essential for FTPE.

Minor Comments:

1. Clarify the physical interpretation of the “effective magnetic field” analogy for readers less familiar with Floquet spin mappings.

2. Typographical issues are minor but should be corrected during revision e.g. occasional spacing and hyphenation inconsistencies.

Reviewer #4

(Remarks to the Author)
This manuscript proposes and experimentally demonstrates Floquet-engineered two-photon excitation (FTPE) as a robust protocol for biexciton preparation in semiconductor quantum dots, enabling high-fidelity entangled photon-pair generation. By employing symmetrically detuned dichromatic ultrafast pulses and interpreting the dynamics within a stroboscopic Floquet Hamiltonian framework, the authors report biexciton preparation efficiencies exceeding 96% and a polarisation-entanglement fidelity of 93.4%.
The combination of rigorous theoretical modelling, including non-Markovian phonon simulations, and convincing experimental demonstrations represents a substantial and timely contribution to the field of solid-state quantum light sources. The work is well positioned with respect to existing excitation strategies, including resonant two-photon excitation, rapid adiabatic passage, phonon-assisted excitation, and SUPER-like dichromatic schemes (“red–red”, “red–blue”, and “blue–blue”).
Subject to clarification of the points below, the manuscript might be suitable for publication in Nature Communications.

Major points
1. The two pulses used in FTPE appear formally identical to those employed in the “red–blue” SUPER-like excitation protocol (Refs.S8, S9, S10, S11). This raises the question of whether FTPE and SUPER represent equivalent physical mechanisms described in different theoretical languages, or whether they operate in genuinely distinct regimes. The authors are encouraged to explicitly clarify the conceptual and operational differences between FTPE and SUPER.
2. When discussing the detuning conditions, the manuscript frequently references the biexciton binding energy. However, since FTPE can also be applied to exciton preparation, it would seem more natural to compare the detuning scale to the exciton radiative linewidth (or decay rate). The authors are encouraged to clarify which energy scale is most relevant for

assessing the operational regime of FTPE and to justify their choice.
3. Are the two pulses in FTPE required to have equal peak intensities? If so, is this a strict requirement or merely an optimal condition? A brief discussion of the tolerance to intensity imbalance, both theoretically and experimentally, would be valuable.
4. Unlike red-detuned excitation, blue-detuned laser pulses can induce phonon-assisted population of exciton and biexciton states. How does such phonon-assisted excitation influence FTPE dynamics?
In addition, since both FTPE and SUPER-like protocols inevitably populate the exciton state during excitation, the authors should clarify how residual exciton population affects the biexciton–exciton cascade and whether this has any measurable impact on entanglement generation or fidelity.
5. The pulse delay is a key control parameter in FTPE. What physical factors determine the optimal delay values (e.g., detuning, pulse duration, amplitude)? If possible, the authors are encouraged to provide:an analytical expression, or a schematic or supplementary figure illustrating how the optimal delay depends on system parameters.
6. As the biexciton population is a central metric for FTPE performance, more experimental detail is required. The authors should clarify how the biexciton population is extracted from the measured signals, how calibration and normalization are performed, and what assumptions enter the conversion from photon counts to population.
7. The authors state that phonon effects play only a minor role in FTPE. In contrast, SUPER requires operation in the phonon-decoupling regime to reach its full potential. A clear physical explanation for this difference would significantly strengthen the manuscript and help position FTPE relative to existing off-resonant excitation schemes.

Minor points
8. Can FTPE operate efficiently in quantum dots with small or negative biexciton binding energy? A brief discussion would be helpful.
9. Reference 32 appears to be incomplete and should be corrected.
10. The acronym STIRAP should be defined upon first appearance in the main text.
11. In Fig. 1c and 1e, the laser excitation spectra exhibit three peaks. Are these features due to spectral interference between the two pulses? Clarification would be useful.
12. In the caption of Fig. 2e and 2f, the condition $\tau = 0$ is missing.
13. The curve shown in Fig. 4g appears not to be fully measured.
14. In Fig. 3b, the amplitude of the Rabi oscillations appears to increase with driving strength. A brief explanation would be appreciated.
15. In Figs. 3b,c and 4b,c, the x-axes use different parameterizations (pulse area vs laser amplitude). For clarity and consistency, the authors may consider unifying the x-axis definitions.

Version 1:

Reviewer comments:

Reviewer #1

(Remarks to the Author)
The manuscript "Robust entangled photon generation enabled by single-shot Floquet driving" by Yan et al. improved a lot in the review. Especially the supplemental, which is now showing really a lot of useful additional facts on the presented scheme, which helps a better understanding for the reader.
I still think the manuscript is extremely interesting as it presents a novel excitation scheme, but some points, especially on the claims of robustness seem to be worse than initially presented. I have some additional comments, that I think should be addressed before a publication of the manuscript:

ad Review comment 1.1:
I want to bring this up again, as the claimed Floquet-regime is one of the central novelties of the manuscript. From a certain perspective, one could write the entire paper without ever invoking the word Floquet, and instead speak about a high-frequency averaging yielding an effective Hamiltonian. On the other hand, the conscious use of the wording Floquet might in fact help the community to spot the limit of applicability of the approach laid out in the manuscript. Here, the large envelope includes more than 10 oscillations, so that a Floquet setup at inverse time scales smaller than the envelope and bigger than the individual oscillations might be justified. It would certainly not be justified with significantly less oscillations under the envelope, and the lack of generality of the approach should be stressed clearly in the manuscript.

ad Review comment 1.3:
My initial comment that it is not surprising that excitation of a spin 0 state works with two photons has not been addressed. The authors worked out differences to other existing schemes, like to SUPER and to dichromatic excitation of a spin 1 state. However, that the scheme at hand can be viewed (or maybe not) as the detuned case of TPE should be addressed or clarified.

ad Reviewer comment 1.7:
To me it is unclear whether between the 3 presented cases in Figure R2 is any fundamental difference in theory, i.e. if there is an abrupt regime change to the FTPE regime somewhere after the double resonant excitation case, or even before, or if there is a smooth transition between the 3 presented cases. This is connected to the newly presented Figure S6 and Reviewer comment 3.1. To me it is still unclear at which point the Magnus-expanded Hamiltonian is not valid any more and whether all 3 presented cases can be described with the FTPE model or if one needs different models for different

detunings? I am asking this, because to me all 3 presented cases in Figure R2 are the same thing, only with different detuning but not fundamentally different. As the main selling point of the paper is to present a new scheme, this should be clarified.

ad Reviewer comment 1.9:
In their new text the authors write about robustness of the FTPE scheme with respect to experimental fluctuations. I think in light of Figure S5, this sentence should be modified to robustness against power fluctuations and quantified, i.e. how much fluctuation can be accepted to be e.g. within 1% of the targeted BX occupation. Furthermore, given the result from Figure R14, as it seems that the power fluctuations have to be in both lasers simultaneously. If only one of the colors fluctuates, then the plateau is left rather fast, so the scheme is maybe not as robust as it is claimed.

ad Reviewer comment 1.11:
I think that the robustness of the scheme under realistic experimental conditions, i.e. the Figure S5 and the corresponding text should be in the main text. The title of the paper is "Robust" and all the presented calculations are backing the robustness with data, but then Figure S5 shows that a very specific detuning has to be used to not run into other excitation regimes such as LA-phonon assisted excitation or excited-state excitation, which is not included in the presented calculations. As this puts the presented scheme into the correct light of how robust it actually is, I believe this paragraph should be not hidden in the supplemental.

ad Reviewer comment 1.15:
I think my original comment was partly misunderstood: my question was whether there is a point on the FTPE parameters, where the effective AC stark shift cancels out and thus makes the entanglement even better than in TPE? As the authors pointed out in the answer to Reviewer Comment 1.16, maybe this is not the right picture, but then in the answer to comment 1.15, they calculated the entanglement fidelity. Thus, the theory is able to capture the effect of the lasers on the FSS, otherwise this calculation would not be feasible. It seems from the answer to comment 1.15 that FTPE is giving worse results for entanglement fidelity than TPE, weakening the presented paper. The answer to this question is thus quite central to the claims of a more robust and better scheme.

ad Review comment 1.16:
the additional figure R14 is quite interesting. Also another reviewer asked the same question. I think presenting Figure R14 with some description in the SI would be useful.

additional comments:
- in Table 1 the robustness is classified as strong, whereas it is moderate for SUPER. I do disagree with this assessment after seeing the additional presented data. I would say that the robustness against common intensity fluctuations is maybe a bit better than in SUPER, but the robustness of differential intensity fluctuations looks like it is worse or maybe comparable to SUPER and the robustness of the detuning is actually much worse than in SUPER, as in SUPER both the pulses are red detuned and one can select freely whatever detuning one wants, as there is no problem with LA-phonons or higher excited states. This is a strong limitation of the presented scheme and should be presented as such. If SUPER is classified as moderate than FTPE should also be moderate or even weak-moderate. Maybe the title should also be reconsidered, as the additional presented data shows that the scheme is not as robust as initially claimed.
- I noticed that the robustness of the entanglement generation is only claimed and not shown, as only a single point of entanglement is measured. As robust entanglement generation is in the title, the authors could maybe measure at least one second point in their parameter space to show that the entanglement generation is indeed robust, or alternatively also remove this claim from the title.

Reviewer #2

(Remarks to the Author)

Reviewer #3

(Remarks to the Author)
Dear Editors,

I have gone through the revised manuscript and authors responses. I feel the manuscript is suitable for publication and I have no further comments.

Reviewer #4

(Remarks to the Author)
The authors have satisfactorily addressed all of the concerns raised during the review process. The manuscript has been significantly improved, and I am pleased to recommend it for publication in Nature Communications.

Open Access This Peer Review File is licensed under a Creative Commons Attribution 4.0 International License, which permits use, sharing, adaptation, distribution and reproduction in any medium or format, as long as you give appropriate credit to the original author(s) and the source, provide a link to the Creative Commons license, and indicate if changes were made.
In cases where reviewers are anonymous, credit should be given to 'Anonymous Referee' and the source.
The images or other third party material in this Peer Review File are included in the article's Creative Commons license, unless indicated otherwise in a credit line to the material. If material is not included in the article's Creative Commons license and your intended use is not permitted by statutory regulation or exceeds the permitted use, you will need to obtain permission directly from the copyright holder.
To view a copy of this license, visit https://creativecommons.org/licenses/by/4.0/

Response letter

We thank the Editor and all the Reviewers for the constructive and insightful comments. We sincerely appreciate the valuable suggestions from all the reviewers, which have significantly improved our manuscript. We have carefully addressed each of the reviewers' comments point by point and revised the manuscript accordingly. Below are our detailed responses to the reviewers' comments. To increase readability, the reviewers' comments are shown in black, our responses are shown in blue, and the actions taken are shown in red.

Reviewer #1 :

The manuscript Robust entangled photon generation enabled by single-shot Floquet driving by Yan et al. is investigating a dichromatic scheme to excite the biexciton state resonantly. I do find the manuscript well written and it adresses a timely topic. The presented results are also backed up by a sound theory and the data is analysed and presented well. However, I do have quite some comments, where I don't understand stuff or things I think should be addressed.

Response: We thank Reviewer #1 for the positive assessment of our work. We greatly appreciate the reviewer's recognition that the manuscript addresses a timely topic and that the results are supported by sound theory and clearly presented data.

We have carefully considered all of the reviewer's questions and concerns, and have revised the manuscript accordingly. Below, we respond to each point in detail.

Review comment 1.1: Firstly, while I am not an expert on Floquet physics, I am not sure that the presented physics has anything to do with Floquet theory. In Floquet theory (as far as I know, as a non-expert), the drive system is continuous and periodic. This is not the case in a pulsed scheme. That two lasers at different frequencies create a beating amplitude is not too unexpected an I am not sure it is a Floquet drive.

Response: We thank the reviewer for this question. As the reviewer correctly points out, usually, Floquet theory is applied to periodic problems, which in our case would correspond to CW driving. This is precisely the novelty of *single-shot* Floquet excitation, where fast oscillations compared to changes in the pulse envelope make the driving field only effectively periodic (however, the dynamics of the state itself is not adiabatic). Here, we picked the name to give credit to established Floquet theory, e.g. [Drese & Holthaus Eur. Phys. J. D 5, 119–134 (1999)] because the physical principles are very much the same: Specifically, the key concept for understanding the efficient state preparation is the stroboscopic Hamiltonian as well as its systematic calculation using Magnus expansion, both developed in the context of Floquet theory.

The beating between the two lasers alone is insufficient to understand the robustness of the excitation for finite delays and it can merely be used as an intuitive picture for zero delay. The single-shot Floquet framework is more general and covers a much wider range of situations.

Review comment 1.2: Furthermore, I also don't see the difference to the SUPER scheme, which does exactly the same. The only difference seems to be that in the SUPER scheme, both pulses are detuned red while here one pulse is detuned red and one is detuned blue. The super scheme is dealing with a two level system, thus the analyis of the excitation of the biexciton is interesting, but the SUPER scheme also uses beating lasers and there it is not a Floquet drive (which I believe is correct - that it is not).

Response: We would like to point out that although there are similarities of SUPER and FTPE in terms of their practical implementation in experiment (two off-resonant pulses are needed), the working principles are fundamentally different.
We would like to draw the reviewer's attention to Supplementary Section XI D, where there is a brief discussion comparing our scheme to SUPER. SUPER considers the excitation of a single two-level system and works by combination of laser dressing and coherent driving of the laser dressed-states. This is not obviously related to a beating effect, and as a result the resonance conditions required for population inversion are different. For FTPE, one uses the beating of two laser frequencies to drive a two-photon transition in a three-level system, where the resonance conditions are not affected by Stark shifts due to laser-dressing. As a side note, more generally analysing SUPER using Floquet theory is technically possible (work in progress), but the resonance conditions for SUPER are such that Magnus expansion breaks down.
A case of SUPER with red and blue detuned pulses is addressed with the reviewer's next question.

Action taken: The discussion on the difference between FTPE and SUPER in Supplementary Section XI D might have been easy to overlook because the section title did not explicitly mention SUPER. We changed the title of this section and added sentences accordingly.

> **Supplementary Information:** SUPER and dichromatic excitation
> ... FTPE has in common with SUPER mainly the fact that two-colour off-resonant excitation is employed. But in contrast to SUPER, the Stark shifts do not set the resonance condition or constitute the working mechanism of FTPE. For the same reason, FTPE also differs from dichromatic excitation with one red- and one blue-detuned laser [S12].

Review comment 1.3: That a dicromatic excitation with two photons will be able to excite

the biexciton state is not too unexpected. Momentum and energy are conserved as long as it is a 2-photon process, as the authors mention. Dichromatic exitaiton of a single line, as e.g. in https://journals.aps.org/prl/abstract/10.1103/PhysRevLett.126.047403, with one of the coautors as coautor, is much more unintuitive, as you can't just absorb one photon from each color as this would break momentum as well as energy conservation.

Response: Indeed, the resonance conditions for dichromatic excitation in the paper mentioned by the reviewer is not just related to the sum of the two frequencies. It also involves the Stark shift of the levels combined with resonant driving of the Stark-shifted levels. Hence, the working principle behind the dichromatic excitation is in fact the same as SUPER, but this was not realized at the time when both articles were published. Hence, also dichromatic excitation with red- and blue-detuned pulses is different from FTPE.

Action taken: In Supplementary Information XI D, we added a statement indicating that FTPE differs from dichromatic excitation in the mentioned article in the same ways as FTPE differs from SUPER.

> **Supplementary Information:** SUPER and dichromatic excitation
> ... FTPE has in common with SUPER mainly the fact that two-colour off-resonant excitation is employed. But in contrast to SUPER, the Stark shifts do not set the resonance condition or constitute the working mechanism of FTPE. For the same reason, FTPE also differs from dichromatic excitation with one red- and one blue-detuned laser [S12].

Review comment 1.4: Besides this very fundamental concern about the title and the use of the word Floquet drive, I do have some more comments on the actual content of the paper:

- in eq. 1: what is E_b? I assume it is the detuning of the laser to the Exciton level, but it is nowhere described.

Response: E_b is the biexciton binding energy with $E_b = 2E_X - E_{BX}$. We have now introduced this quantity clearly in the main text immediately after Eq. 1 and have checked the manuscript to ensure that all symbols are defined when first introduced. In addition, we also add a table in the Supplementary Information to clearly show the physical symbols, their meanings, and their corresponding values.

Action taken: The definition of symbols E_b has been included, as well as a table in Supplementary Information (see Fig. R1 below) showing the main symbols, their meanings, and their corresponding values.

> **Main text:** "... The energy of $|BX\rangle$ is $E_{BX} = 2E_X - E_b$, where E_X is the energy of the X state and E_b is the biexciton binding energy. ..."

Supplementary Information: "The main parameters used in the calculations are summarized in Supplementary Table 1. ..."

Supplementary Table 1. Parameters used for the calculations.

Parameter	Symbol	Value
Biexciton binding energy	E_b	2.82 meV
Intrinsic fine-structure splitting	FSS	3.4 μeV
Electronic radius	a_e	5.5 nm
Hole radius	a_h	$a_e/1.15$
Pulse electric-field duration	τ_0	12.49 ps
Pulse detuning	$\hbar\delta$	3.75 meV
CW laser detuing	$\hbar\delta_{CW}$	300 μeV

Figure R1: Supplementary Table 1

Review comment 1.5: - I don't understand how the authors arrive at Eq. 2. Furhtermore, I do not understand if the hamiltonian is now an effective magnetic field hamiltonian, where I don't know why electric fields can be neglected or if the magnetic field is just an anology. Maybe this could be discussed better.

Response: The key idea behind our Floquet theory is that it allows us to replace the fast oscillating and complicated dynamics of the physical three-level system composed of ground state, exciton, and biexciton state, more abstractly as a slow evolution of a two-level on a coarse-grained time scale, where the two levels physically correspond to the ground and biexciton state. The dynamics is described by an effective stroboscopic Hamiltonian, which itself slowly changes in time, and whose matrix elements are related to the driving of the original three-level system in a way described in the Methods Section.
The "effective magnetic field" is a way to represent a general 2x2 Hamiltonian, and it helps to visualize the dynamics as the state vector on the corresponding Bloch sphere precesses about the instantaneous effective magnetic field. As a theoretical construct, it is unrelated to any physical magnetic field.

Action taken: We rephrased the corresponding paragraph in the main text to highlight the formal nature of the effective magnetic field.

Main text: ... The working principle of our FTPE scheme can be understood by Floquet theory with respect to the period $T = 2\pi/\delta$. For detunings δ large enough so that $T \ll \tau_0$, fast oscillations can be eliminated and H is replaced by a stroboscopic Hamiltonian $H^{\text{eff.}}$. If moreover δ is the dominant energy scale in the Hamiltonian, $\delta \gg E_b/\hbar, f$, the effective Hamiltonian $H^{\text{eff.}}$ can be obtained by systematic expansion in $1/\delta$ [48,49]. This procedure yields a term that directly

couples the ground to the biexciton state. The residual impact of the excitonic state is further eliminated by a Schrieffer-Wolff transformation, and we arrive at an effective Hamiltonian involving only ground and biexciton-like states with slowly varying driving envelopes. The details of this calculation can be found in the methods section. Formally, such a two-level system can be regarded as a spin precessing in a fictitious time-dependent effective magnetic field $B(t)$, which is described by the Hamiltonian

$$H^{\text{eff.}}(t) = \frac{\hbar}{2}\left(B_x(t)\sigma_x + B_z(t)\sigma_z\right), \tag{1}$$

Review comment 1.6: - As a positive comment: I do really like the explanation of the popolation transfer in Fig. 2 and the corresponding part in the main text. I think this gives some understanding of what is happening instead of just presenting the outcome.

Response: We thank the reviewer for this positive comment. We are pleased that the explanation of the population-transfer dynamics in Fig. 2 was found helpful and informative.

Review comment 1.7: - I wonder why in figure 3 the lowest pulse area to achieve high BX occupation is at around 1.7 meV detuning. This seems to be even lower pulse area than for the resonant TPE case with delta=0.

Response: The lowest pulse area to achieve high BX occupation is at around 1.41 meV, which is actually equal to $E_B/2$ (as shown in Fig. R2 b). It corresponds to blue and red lasers being on resonance with the G-X and X-BX transitions, respectively. It's a double resonant excitation process, with single-photon and two-photon resonant conditions both satisfied, thus leading to the most effective excitation of BX than TPE and FTPE (Fig. R2 a and c).

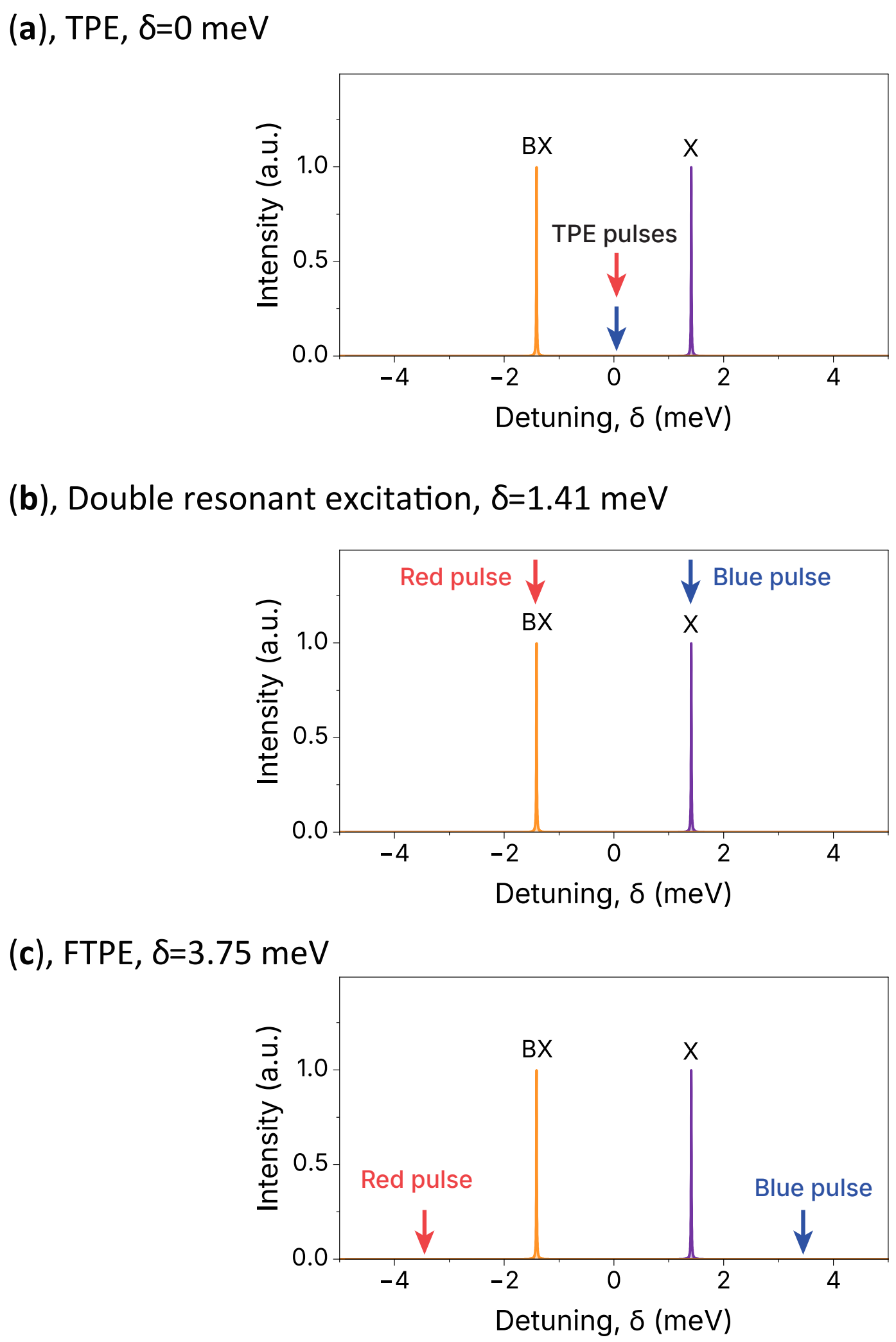

Figure R2: Comparison of pulses and transition lines under different pulse detuning δ.

Review comment 1.8: I further wonder how the pulse area is normalized, as I would expect system inversion in the resonant TPE case for a Pi pulse. In the presented results this seems to be at about 4pi.

Response: The pulse area in Fig. 3 corresponds to individual pulse coupling with a single-photon transition. The individual pulse area is defined as: $\Theta = \left|\frac{\mu}{\hbar}\int_{-\infty}^{+\infty}\mathcal{E}_0(t)\mathrm{d}t\right|$, where μ is the transition dipole moment for G-X or X-BX transitions, and $\mathcal{E}_0(t)$ is the laser electric field amplitude of the blue or red pulse. For BX preparation, the two-photon effective pulse area Λ is a nonlinear function of the original single-photon pulse area Θ. When $\tau = 0$ ps, it could be represented as:

$$\Lambda := \int_{-\infty}^{\infty} H^{\text{eff}}_{BX,0}(t)\,\mathrm{d}t = \frac{E_b}{8\sqrt{\pi}\hbar\delta^2 s}\left(1 - \frac{E_b^2}{8\hbar^2\delta^2}\right)\Theta^2. \tag{2}$$

The final BX occupation is presented as $|\langle BX|\psi(t=\infty)\rangle|^2 = \sin^2\left(\frac{\Lambda}{2}\right)$ Therefore, the π-pulse condition, i.e., the system inversion referred to by the reviewer, for resonant TPE corresponds to $\Lambda = \pi$ and $\Theta =\sim 2.7\pi$.

For clarity and to maintain a direct linear correspondence with the experimentally measured pulse amplitude, we still use the original single-photon pulse area Θ in the figures, as has also been done in some previous classic works, e.g., Ref. [Phys. Rev. B 73, 125304 (2006)]. We have added sentences in the main text that clarify the definition of the single-photon pulse area Θ, and have also included a detailed derivation of the effective two-photon pulse area Λ in the Methods section.

Action taken:

> **Main text:** ... The pulse area is defined as $\Theta = \left|\frac{\mu}{\hbar}\int_{-\infty}^{+\infty}\mathcal{E}_0(t)\mathrm{d}t\right|$, where μ is the transition dipole moment for each single-photon transition, and $\mathcal{E}_0(t)$ is the electric-field amplitude of either the blue- or red-detuned pulse. ...
>
> ... the effective pulse area Λ scales as $\sim \Theta^2$ instead of the usual linear scaling (cf. Eq.9 in the Methods Section). For finite delay τ, $B_z(t)$ becomes nonzero [Fig.2(b) and (d)], which facilitates the engineering of more complex dynamics. ...

Review comment 1.9: - In Fig. 3 c, the experimental state preparation for FTPE seems to be significantly higher than the TPE, while in the calculation of Fig. 3 b, they seem to reach similar values. Where does this discrepancy come from?

Response: We thank the reviewer for pointing this out. After carefully re-examining the measurements, we found that this discrepancy may arise from day-to-day variations in the overall system efficiency, since the FTPE and TPE datasets in the original version were acquired in separate measurement sessions. To verify this, we repeated a complete set of measurements for both FTPE and TPE within a short experimental period and a consistent experimental condition. In the updated results, the maximum biexciton occupations obtained under TPE and FTPE are 0.968 and 0.966, respectively, in agreement with the theoretical prediction that the two schemes reach comparable peak occupations.

We also state that the residual small discrepancy between the theoretical and experimental peak occupations may arise from the uncertainty in the effective phonon-coupling coefficient extraction, and attribute the slight decrease of the experimental curve in the high-power regime to additional excited-state channels, such as hole-state excitation, that are not included in the present model.

Action taken: We have replaced the original Fig. 3c with a new TPE and FTPE dataset acquired under the same experimental conditions and using a consistent calibration procedure. The revised figure now shows that TPE and FTPE reach comparable maximum biexciton

occupations, while FTPE retains the enhanced robustness against pulse-area variations. We have also revised the corresponding discussion in the manuscript accordingly.

> **Main text:** ... As shown in Fig. 3(c), the first maximum of the BX occupation under TPE (black dots, 96.8%) occurs at a per-pulse area of 2.7π. The maximum BX occupation achieved under FTPE is 96.6% and appears at a per-pulse area of 15.9π. Importantly, the achieved final occupation shows significantly reduced sensitivity to variations in laser amplitude. This robustness to experimental fluctuations underscores the advantages of the FTPE scheme for practical quantum information applications. The small discrepancy between the theoretical and experimental peak occupations may arise from the uncertainty in the effective phonon-coupling coefficient extraction, as discussed in Supplementary Section IV. The slight decrease of the experimental curve in the high-power regime may be related to additional excited-state channels, such as hole-state excitation (Supplementary Section VI), that are not included in the present model.

Review comment 1.10: - In the main text the authors claim 98.7 % preparation efficiency for the plot in Fig. 3a. From the figure itself it seems like the value is not much higher than 0.9, but this is because it is very hard to tell how dark red the red actually is. I suggest to maybe plot another contour level at 95% (or even higher).

Response: We thank the reviewer for this constructive suggestion. We agree that, in the previous version, it was difficult to visually distinguish the highest-efficiency region from the color scale alone. To improve clarity, we have revised the plot by adding a higher contour level at 95%, as shown in Fig. R3, which makes the high-efficiency region easier to identify.

In addition, because the relevant datasets were remeasured and the phonon-coupling parameters were correspondingly updated, the maximum population values have also been revised. In the updated model, the maximum biexciton occupation reaches 96.7% when phonon coupling is included, and 100% in the absence of phonons. We note that phonon coupling is the only mechanism in the present model that prevents unity occupation.

Action taken: We have revised Fig. 3(a) by adding a 0.95 contour line to make the high-efficiency region more clearly visible. We have also updated the experimental data and model parameters based on the remeasured dataset.

Review comment 1.11: - For measuring the data of fig 3c and fig 4, I wonder why 3.75 meV detuning was chosen. It seems from Fig 3a that the optimal detuning for maximum BX excitation would be at about 5 meV.

Response: We thank the reviewer for this question. In principle, FTPE can operate over

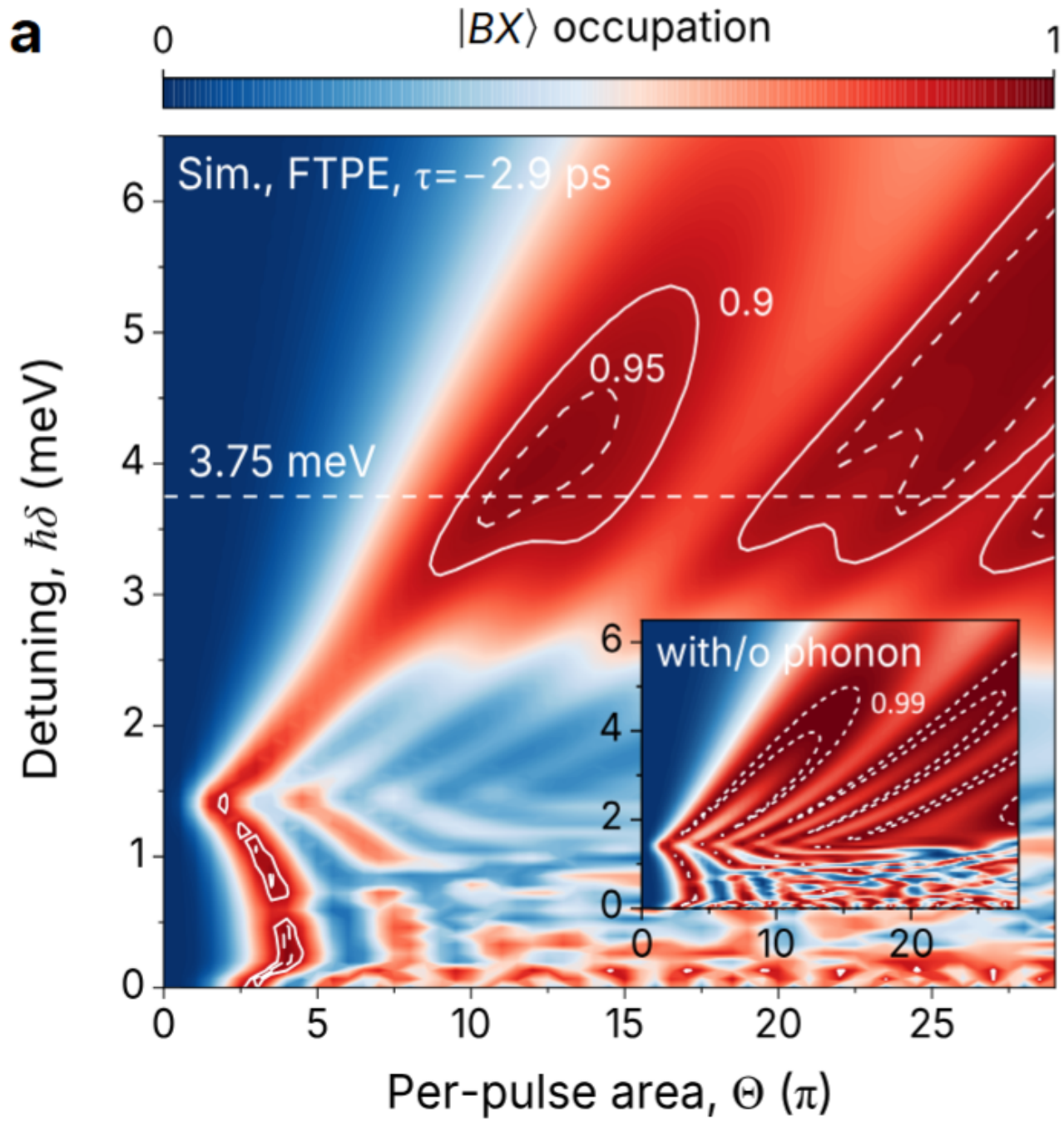

Figure R3: The revised Fig. 3(a).

a relatively broad detuning range within the four-level model. In a practical QD system, however, higher excited states may also participate and induce unwanted population of the exciton state, such as the electron p-shell to hole p-shell transition and the electron s-shell to hole p-shell transition. While the former is typically about 25 meV above the lowest transition (i.e., electron s-shell to hole s-shell), the latter can be only about 5 meV higher in energy [Phys. Rev. B 100, 155420 (2019)].

Experimentally, the choice of a detuning of 3.75 meV was guided by high-power photoluminescence excitation (PLE) measurements, in which we applied a monochromatic pulse and continuously swept the detuning while monitoring the exciton (X) emission intensity. We observed that, when the detuning exceeds 4.5 meV, several additional resonances associated with the electron s-shell to hole p-shell transition begin to appear, as shown in the blue regime of Fig. R4. These interband cross-shell transitions are nominally forbidden by the envelope-function parity/orthogonality selection rule but weakly allowed in realistic QDs [Phys. Rev. B 74, 045316 (2006)]. Similar behavior has also been reported in a closely related QD system [Phys. Rev. B 100, 155420 (2019)]. Such hole-state excitation is expected to be less pronounced in more strongly confined InAs quantum-dot systems, where the transition selection rules are typically stricter. On the other hand, for smaller detunings, phonon-assisted excitation becomes increasingly strong, as also captured by our simulations.

Therefore, to suppress both phonon-assisted excitation at small detuning and higher-state excitation at larger detuning, we chose 3.75 meV as the working detuning for demonstrating

the reliability of the FTPE scheme. To make this point clearer, we have also added a Supplementary Section discussing this trade-off and the experimental basis for choosing the detuning.

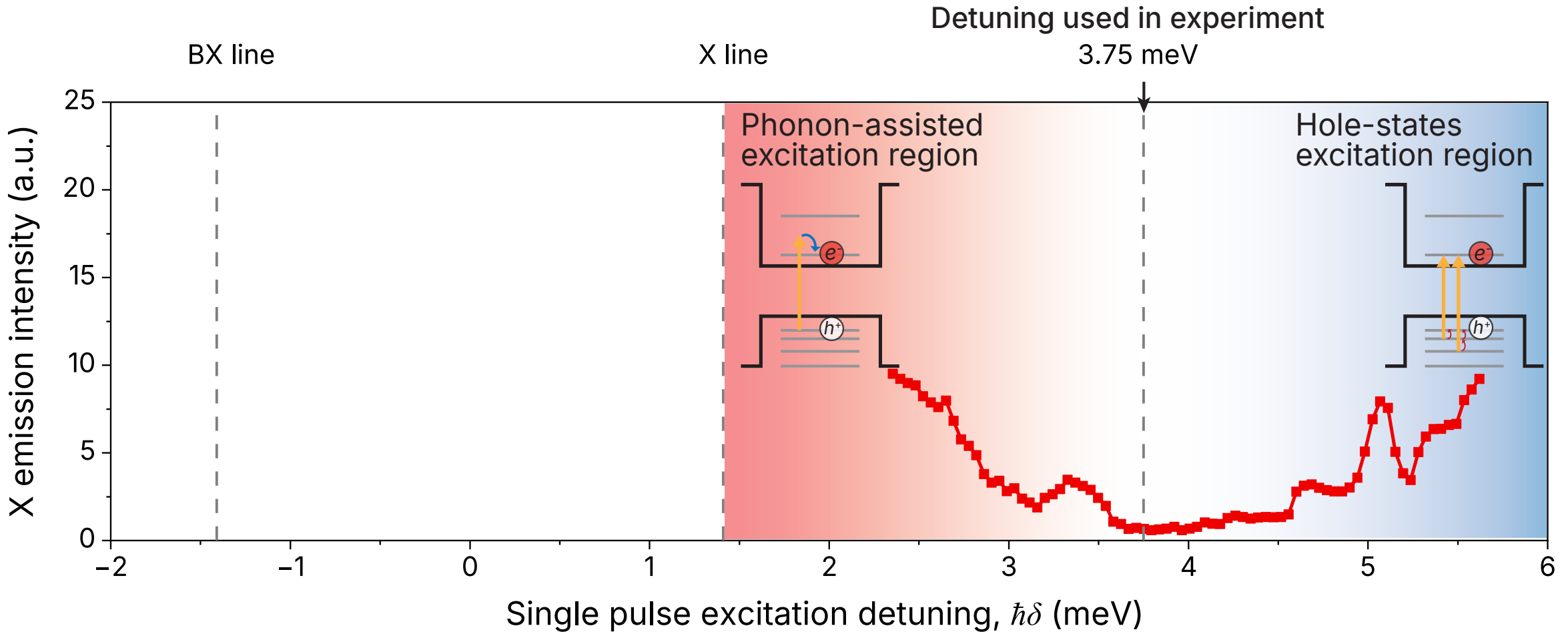

Figure R4: High-power monochromatic pulse PLE measurement.

Action taken: We have added a discussion in Supplementary Information to clarify that the choice of 3.75 meV reflects a practical compromise between suppressing phonon-assisted excitation at smaller detuning and avoiding higher-state excitation at larger detuning.

> **Supplementary Information:** ... In principle, FTPE can operate over a broad range of laser detunings, as long as the two-photon resonance condition is satisfied and sufficient effective two-photon coupling is maintained. In the experiment, the practically accessible detuning range is limited by additional excitation channels in the weak-confinement GaAs/AlGaAs QDs system. To determine a suitable laser detuning for the FTPE measurements, we measured the X intensity under high-power single-pulse excitation as a function of detuning. As shown in Supplementary Fig. 5, for detunings smaller than about 3 meV, the X state can still be populated through LA-phonon-assisted excitation. At larger detunings than 4.5 meV, several resonant features appear, which we attribute to hole-state excitation [S3]. We therefore chose a detuning of 3.75 meV for demonstrating the reliability of the FTPE scheme as a compromise, where phonon-assisted excitation is largely suppressed while prominent hole-state excitation features are avoided.
>
> The hole-state excitation could arise from structural asymmetry, strain-induced band mixing, and many-body configuration mixing [S4, S5], and is not included in our four-level theoretical model. This restriction is specific to the present material system rather than an intrinsic limitation of the FTPE scheme. For more strongly confined InAs/GaAs QD systems, the unwanted hole-state excitation is expected to

be weaker, which should allow FTPE to be implemented at larger detunings and over a broader practical detuning window. ...

Review comment 1.12: The authors report to measure 96% BX occupation at 37.7 µW, -1.5 ps and 3.75 meV detuning. From Fig 3a it seems that the theoretical maximum for this detuning and timing is at about 92%. How can the experimental value be over the theoretical achievable one?

Response: We thank the reviewer for this question. In the previous version of the manuscript, the TPE and FTPE datasets were measured at different times, which may have introduced a small inconsistency in the comparison due to changes in the overall experimental efficiency over time. To eliminate this issue, we remeasured the relevant TPE and FTPE data within a short time interval under consistent experimental conditions. In the revised manuscript, for $\tau = -2.9$ ps and $\hbar\delta = 3.75$ meV, the simulated peak value is 95.7%, while the experimentally measured values are 96.6%. Since phonon coupling is the only mechanism included in the present model that limits the maximum BX population below unity, we attribute the remaining small discrepancy mainly to the uncertainty in determining the effective phonon-coupling coefficient. As shown in Fig. R5, the relevant coefficient a_e is obtained by fitting the damping of the TPE Rabi oscillation, which may introduce some uncertainty in the simulated maximum BX population.

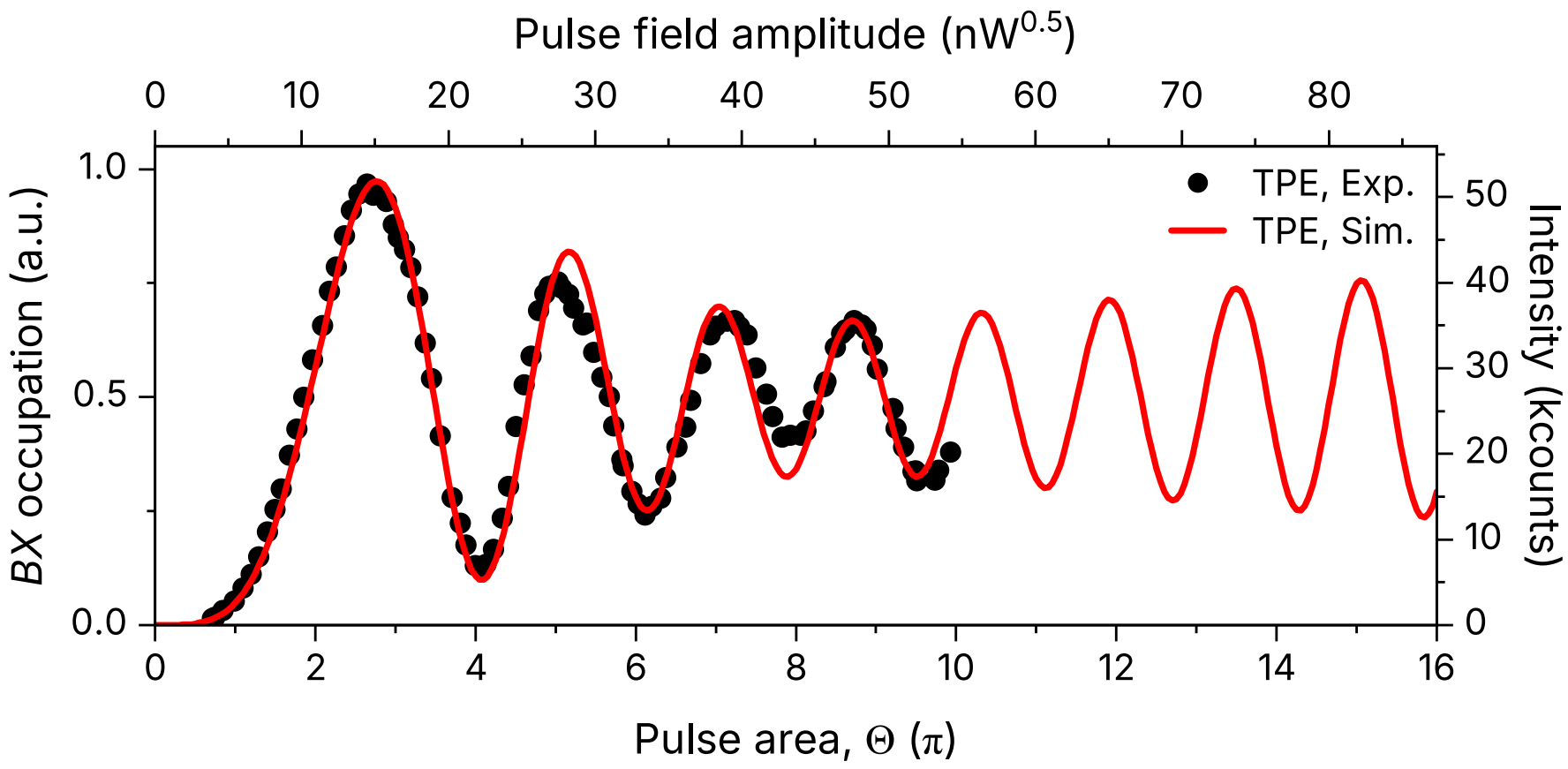

Figure R5: Experimental TPE Rabi oscillation data are fitted to the simulated BX occupation as a function of pulse area. The fitting procedure determines the conversion factor from measured intensity to BX occupation, the calibration factor from pulse field amplitude to pulse area, and the radius of the electronic wavefunction.

Action taken:

We have replaced the previous TPE and FTPE datasets with remeasured data acquired within a short time interval under consistent experimental conditions. We have also added a clarifying

discussion in the revised manuscript noting that the remaining small difference between the measured and simulated maximum BX population can arise from the approximate determination of the phonon coupling coefficient.

> **Main text:** ... The small discrepancy between the theoretical and experimental peak occupations may arise from the uncertainty in the effective phonon-coupling coefficient extraction, as discussed in Supplementary Section IV. ...
>
> **Supplementary Information:** ... We note that since phonon effects are not very pronounced at the moderate excitation powers used for this fitting, the extracted effective phonon-coupling parameters may be subject to some uncertainty, which could introduce a small error in the calibration.

Review comment 1.13: I further wonder, how the authors experimentally verify the occupation probability. It would benefit the paper a lot if they can elaborate on that. Often the experimental data is compared to a theoretical fit to determine the inversion efficiency, but here it seems that that number was found independent, as it is comparing experiment to theory.

Response: We agree that the procedure of occupation probability normalization should be explained more clearly. Experimentally, the occupation probability was calibrated by fitting the experimental TPE map to the simulated one using two scaling factors along the horizontal and vertical axes, as shown in Fig. R5. From this calibration, we obtained the proportionality factor between the measured BX emission intensity and the corresponding BX state population, as well as between the experimental pulse amplitude and the pulse area used in the simulations. These proportionality factors are then used to normalize the power-dependence curve under FTPE.

Action taken: We have added a discussion in the revised manuscript to note that the small difference between the measured and simulated maximum BX population can arise from the approximate determination of the effective phonon coupling matrix.

We have also added a Supplementary Section to explain how the occupation probability is extracted from the experimental data.

> **Main text:** ... The experimentally achieved occupations and pulse area are calibrated by fitting the TPE Rabi oscillation data to numerical results with two scaling factors, as shown in Supplementary Fig. 4. ...
>
> **Supplementary Information:** ... To quantitatively compare the experimentally measured emission intensity with the simulated occupation, we calibrated the experimental TPE data by establishing the correspondence between the experimental observables and the theoretical parameters. As shown in Supplementary Fig. 4,

the measured TPE emission intensity was fitted to the simulated occupation as a function of pulse area. In this fitting procedure, the conversion from measured photon counts to occupation and the conversion from the square root of the excitation power to pulse area were treated as free scaling parameters. In addition, the radius of the electronic wavefunction, (a_e), was also included as a fitted parameter in the simulation model.

The fit yields a conversion factor of ($0.0188\,\mathrm{kcounts}^{-1}$) from the measured emission intensity to the occupation, a pulse-area calibration factor of ($0.1832\,\pi/\mathrm{nW}^{0.5}$), and an electronic wavefunction radius of ($a_e = 5.5$ nm). These fitted parameters were then used in all relevant analyses throughout this work. ...

Review comment 1.14: - The authors find -1.5 ps to be the optimal delay. I wonder how this changes with excitation pulse length and detuning, as these were fixed parameters.

Response: We thank the reviewer for this question. Qualitatively, robust excitation is indicated by a large patch in parameter space where near-unity state preparation fidelity is observed. How exactly to quantify the precise optimum is not a well-defined question as there may be a trade-off between the size of the patch and the admissible distance to unity state preparation fidelity. Moreover, even using Magnus expansion, the expressions for finite detuning are complicated and depend on many parameters. This makes providing an analytical expression extremely difficult.

We find that the dependence of robust values of τ on the pulse duration are quite intuitive. As the pulses become longer they need longer delays to stay robust. In contrast, a higher detuning δ needs a smaller delay.

Action taken: In order to give a visual impression about the robustness for different delay times, we provide two additional figures in the Supplementary Section IX showing color plots of population for various values of τ.

Review comment 1.15: - On the entanglement measurement: if the authors apply such strong power to drive the system, I wonder how this affects the AC stark shift of the driving laser itself, as found to be detrimental for entanglement generation. (https://doi.org/10.1103/PhysRevLett.131.166901). I don't know if the authors have enough counts and time resolution to look at this effect in the time domain, but it should also be observable in the spectrum as a shoulder on the biexciton line. Maybe having 2 lasers with red and blue detuning to the exciton is partly cancelling the AC stark shift of the exciton state.

Response: We thank the reviewer for this comment. In our system, the excitation-pulse duration (~12.5 ps) is much shorter than the biexciton lifetime (~200 ps). As a result, only a

small fraction of the biexciton decay takes place during the time window in which the pulsed excitation field can induce an ac Stark shift. We therefore expect the excitation-pulse-induced ac Stark effect to have a much weaker impact on the measured entanglement than in systems where the pulse duration is comparable to the radiative lifetime.

Consistent with this expectation, we do not observe an obvious shoulder feature associated with the pulsed excitation in the measured emission spectra (see Fig. 1c and 1e in the main text). This is in contrast to Ref. [Phys. Rev. Lett. 131, 166901 (2023)], where the biexciton lifetime (~35 ps) is comparable to the laser-pulse duration (~20 ps), such that a substantially larger fraction of the emission is generated while the ac Stark shift is active.

To quantify this effect, we carried out simulations of the entanglement fidelity under FTPE with and without the CW laser used for FSS compensation, as shown in Fig. R7. In the absence of the CW laser, the calculated fidelity is 97.7%. This level is very close to that obtained for TPE (98.2%), indicating that FTPE does not introduce a significantly stronger ac Stark than conventional TPE. When the auxiliary CW laser is included, the entanglement fidelity is further reduced to 95.3%.

Overall, these results indicate that FTPE pulse-induced ac Stark effects impose a modest limitation on the fidelity, reducing it from the ideal value to a level comparable to that of TPE, but without severely compromising the entanglement performance. The additional CW laser used for FSS compensation leads to a further 2.4% reduction.

Action taken: We have included simulation results in the Supplementary Section VII to separately assess the influence of the pulsed FTPE driving and of the auxiliary CW laser used for FSS compensation on the entanglement fidelity. We have also revised the discussion in the manuscript to clarify that pulse-induced ac Stark effects reduce the fidelity to a level comparable to TPE. While the additional reduction in the present experiment is caused by the CW-induced re-excitation channel.

> **Main text:**... The measured fidelity is close to our simulated value (95.3%) (Supplementary Section VIII), from which we attribute about 2.4% of the fidelity reduction to re-excitation induced by the CW laser. This effect is also supported by the power dependence of the autocorrelation measurements (see Supplementary Section III). The remaining reduction of about 2.3% arises from the intrinsic pulse-induced transient spectral shift during the FTPE driving itself. ...

Review comment 1.16: It would be very interesting to see whether the proposed scheme also works in the case where the laser that is less detuned with respect to the exciton is much weaker, such that the two laser's AC stark shifts cancel each other out. Maybe this could be investigated in theory?

Response: Analyzing Stark shifts is very useful for excitations using a single laser pulse. There, Stark shifts are obtained by going into the rotating frame of the laser and diagonalizing the Hamiltonian. However, the situation is much more complicated for two simultaneous pulses of similar strength because there is no rotating frame that eliminates both laser oscillations simultaneously. Consequently, also the concept of Stark shifts becomes ambiguous and requires more care. In Floquet theory, the closest analogy to Stark shifts are the shifts of the diagonal elements in the stroboscopic Hamiltonian. However, also the oscillatory dynamics on the fast time scale may affect the properties of the emitted photons. A comprehensive picture of the impact of various tuning parameters is much more challenging than the analysis of Stark shift for TPE.
To address the question of different pulse areas for the two pulses, we show in Fig. R14 in this response the excitation efficiency obtained when varying the pulse areas independently. The highly symmetric structure suggests that deviating too much from excitation with equal pulse areas likely suppresses the efficiency of the protocol.

Review comment 1.17: I further wonder how far detuned the additional, quite strong cw laser is, that the authors use for the compensation of the FSS.

Response: The detuning of the additional CW laser used for FSS compensation was set to 300 μeV. This information is now explicitly stated in the main text and summarized in the Supplementary Table 1.

Action taken: We have clarified in the revised main text and Supplementary Table that the detuning of the CW laser used for FSS compensation is 300 μeV.

> **Main text:** ... The intrinsic FSS of 3.4(2) μeV is eliminated by the ac Stark effect induced by the 13.5 μW CW laser with a red detuning of 300 μeV. ...

Review comment 1.18: And I wonder whether this laser interacts somehow with the exitation pulses for additional exitation mechanisms, as it will beat with the applied pulsed laser as well. Is this taken into account into the presented theory?

Addressing the AC stark shift on the entanglement is one of the major missing pieces in the manuscript, as the proposed scheme needs quite a lot of power and if it therefore introduces a large AC stark shift, it would be hindering the creation of high fidelity entanglement. This becomes even more an issue if dealing with high-performance sources such as QDs in Purcell enhanced microcavities, as when the lifetime is significantly shortened, the portion of the light that experiences the stark shift is becoming larger. A scheme that could compensate for that would be of utmost interest to the community! I think this has to be properly discussed to evaluate the real strengths and weaknesses of the proposed scheme.

I think if the above points are addressed properly, the manuscript should be published as it shows very interesting physics.

With best regards, Prof. Dr. Tobias Huber-Loyola

Response:

As far as the excitation efficiency of FTPE is concerned, we find that the laser compensating the FSS interferes only little with the driving fields. The slight mixing between the ground state, the exciton state with the same polarization of the laser, and the biexciton state yields a slightly renormalized three-level system, whose eigenstates can be prepared using TPE or FTPE just like a system without FSS and without compensating laser. This is shown here by numerical simulations of FTPE in Fig. R6.

If, however, the second exciton state is accounted for, e.g., to obtain the entanglement of the emitted photons, the mixing of states due to the CW laser leads to additional re-excitation channels which partially reduce the entanglement. In analyze the impact of the CW laser, we show in Fig. R7 the simulated concurrence of the emitted photons for TPE and FTPE with and without time delay, where in the upper panel we account for the FSS and the CW laser, while in the lower panel, we consider no FSS and no compensating laser. We find that the concurrence of the emitted photons suffers somewhat from a finite FSS even if it is compensated by a CW laser. This also indicates that devices that allow for, e.g., strain tuning of FSS would yield a higher concurrence when excited with FTPE. The same holds for TPE. Photons emitted after FTPE have only slightly lower concurrence then photons emitted after TPE.

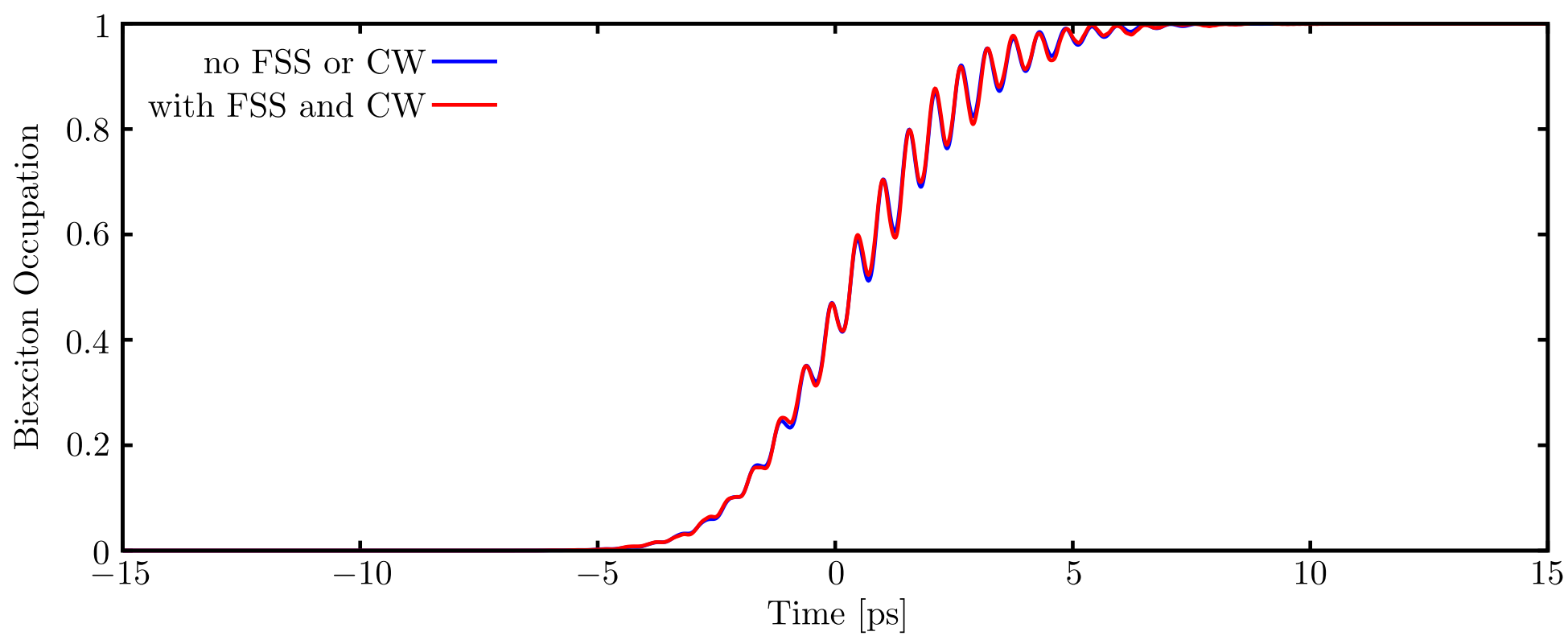

Figure R6: Time-dependent behavior of the biexciton occupation without (blue) and with (red) FSS or CW. The delay was chosen as $\tau = -2.9$ ps. These particular calculations include neither losses nor phonons.

Action taken: We have added the investigation of the entanglement fidelity in the Supplementary Information on the influence of the CW laser on the entanglement fidelity. In particular, we included simulations comparing the BX preparation dynamics with and without the CW laser, as well as calculated entanglement fidelities for TPE and FTPE in both cases.

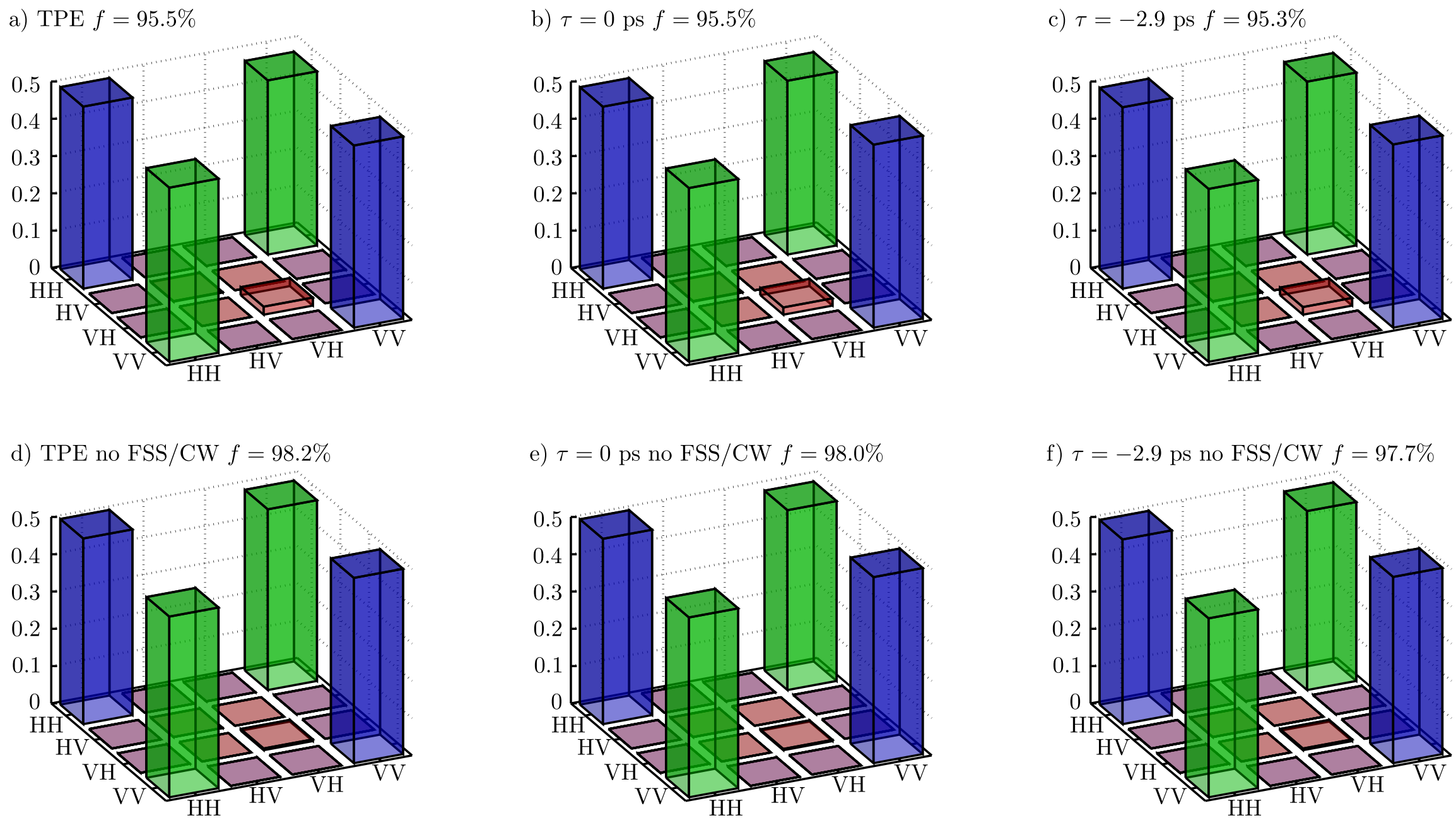

Figure R7: Entanglement fidelities and density matrices in the polarization basis for TPE, as well as FTPE with $\tau = 0$ ps and $\tau = -2.9$ ps. All calculations were done without phonons. a)-c) are calculated with FSS and the CW laser to compensate it and d)-f) are calculated without either.

Supplementary Information: Theoretical entanglement fidelity

We calculated the entanglement fidelity of FTPE for $\tau = 0$ ps and $\tau = -2.9$ ps and compared them to TPE. This is done for two cases: In one, the FSS from the experiment is compensated for using a CW laser and in the other, no FSS was present. The resulting fidelities and density matrices in the polarization basis are shown in Supplementary Fig. 7. The entanglement fidelities achieved by FTPE are on par with TPE, though a small decrease of a fraction of a percent can be observed when using delayed pulses. When the FSS is compensated for by the CW laser, the fidelity generally decreases for all considered protocols. We attribute this to the laser-induced dressing of the QD states. In particular, the exciton gains a slight biexcitonic component, which allows for rate transitions between the excitons. This decreases fidelity. This shows that the experimentally obtained value for the fidelity can be increased considerably when using other mechanisms to compensate for the FSS.

Reviewer #2 :

The manuscript describes the theory and experiment addressing a very important problem in photonic quantum information technology: the generation of robust, on-demand, high-fidelity entangled photon pairs. Although this is a well-studied topic, the manuscript introduces a genuinely new perspective to the field and merits publication in Nature Communications. The material is well presented, easy to follow, and both the experimental and theoretical methods and data appear convincing. The material is also well illustrated.

Response: We thank Reviewer #2 for the positive and encouraging assessment of our work. We greatly appreciate the reviewer's recognition that the manuscript addresses an important problem in photonic quantum information technology, and that it offers a genuinely new perspective with convincing theoretical and experimental results.

We have carefully addressed the reviewer's comments and suggestions, and have revised the manuscript accordingly. Our detailed responses are provided below.

Review comment 2.1: I have two minor points concerning readability: -T=2\pi / delta should be defined upon the first mention of T, for instance, at the beginning of the section "Theory on Floquet-engineered TPE," as this clarifies its physical meaning.

Response: We politely draw the reviewer's attention to the first occurrence of the period T in the context of FTPE in

> **Main text:** ...The working principle of our FTPE scheme can be understood by Floquet theory with respect to the period T = $2\pi/\delta$. ...

In the earlier occurrence of the period in the introduction, we merely explain Floquet theory and not our specific system, such that the reader is not yet familiar with δ at that point.

Review comment 2.2: -On page 3, after Eq. 2, the physical meaning of σ_x and σ_z should be properly introduced (even if known to most readers).

Response: We thank the reviewer for this helpful suggestion. We agree that the physical meaning of σ_x and σ_z should be stated explicitly, even though these operators are commonly used in effective two-level descriptions. In our case, they act in the reduced two-level subspace spanned by the ground and biexciton states. Here, σ_x represents the effective transverse coupling between these two states, while σ_z describes their effective energy detuning. We have clarified this point in the revised main text.

Action taken: We have added an explicit explanation of σ_x and σ_z in the main text after Eq. (2).

> **Main text:** ... Here σ_x and σ_z are Pauli matrices acting in the reduced two-level

subspace spanned by the ground and biexciton states. The σ_x describes the effective coupling between these states, while the σ_z represents their effective detuning. ...

Review comment 2.3: I also have three points–important in my view–that should be addressed before the paper is published:

a) Given the vast amount of work on entanglement in quantum dot emission (which uses similar material compositions), I–and I assume many readers not directly involved in this specific field of research–would appreciate a comparison of fidelity, preparation efficiency, and other metrics from different works/methods. Including experimental and theoretical figures in the "Discussion and Conclusion" section would help us judge the superiority of this work, as many proposals exist, also explained in the supplement.

Response: We thank the reviewer for this valuable suggestion. We have therefore added a table summary in the revised "Discussion and Conclusion" section to compare FTPE with representative excitation schemes used for entangled-photon generation in QDs, including resonant TPE / phonon-assisted TPE / ARP / SUPER. In particular, we summarize the preparation efficiency in theory and experiment, entanglement fidelity, robustness, and practical filtering difficulties, as shown in Fig. R8.

Action taken: We have added a comparison summary table in the "Discussion and Conclusion" section, positioning FTPE relative to previously reported excitation methods for QD-based entangled-photon generation.

TABLE I. Comparison of existing BX preparation schemes in semiconductor QDs

Scheme	Preparation efficiency		Entanglement fidelity	Ref.	Robustness	Filtering difficulty
	Theory	Experiment	Experiment			
TPE	> 99% without phonons	~ 82%	–	[12]	Weak	Moderate
	–	~ 90%	88%	[14]		
	–	n.q.	97.8%	[13]		
Phonon-assisted TPE	> 99%	–	–	[44]	Strong	Difficult
	–	> 80%	–	[29]		
	–	95%	–	[28]		
	–	> 80%	90%	[60]		
ARP	> 90% at 4 K<br>> 98% at 1 K	–	–	[24]	Strong	Difficult
	–	> 95%	–	[61]		
SUPER	> 99% without phonons	–	–	[62]	Moderate	Easy
	> 95% at 4 K	–	–	[30]		
	–	n.q.	–	[63]		
FTPE	> 99% without phonons<br>96.7% at 3.6 K	96.6%	93.4% (97.7%)[a]	This work	Strong	Easy

[a] Ideal fidelity without FSS and CW laser. n.q., not quantified. –, not reported.

Figure R8: Table 1. Comparison of existing BX preparation schemes in semiconductor QD.

Review comment 2.4: b) Can the authors comment on the influence of zero-phonon line broadening on the scheme? This is often a critical aspect that differs from one dot to another.

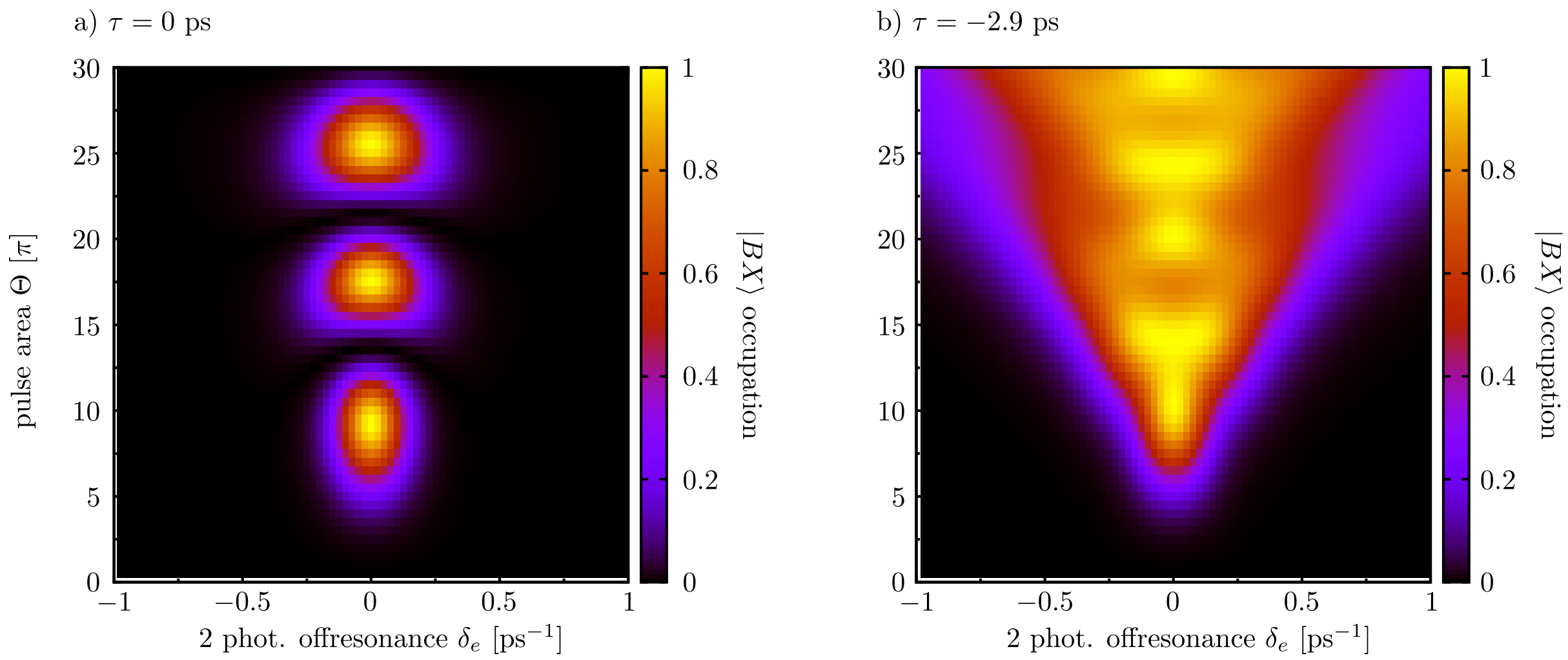

Figure R9: Biexciton occupation occupation for different pulse areas and detunings from the 2-photon resonance for $\tau = 0$ ps and $\tau = -2.9$ ps. These calculations were done without phonons or losses.

Response: If charges near the QD shift the energy levels slightly, then the two-photon resonance that FTPE depends on can be broken. To investigate the severity of this spectral-wandering effect, we calculated the influence of such a detuning, we shifted both lasers by a frequency δ_e. The resulting heatmap, where the pulse area was also varied, is shown in Fig. R9. When the pulses are simultaneous, detunings of $\delta_e \sim 0.1$ ps lead to significantly worse excitation efficiency. If, however, a finite delay, like $\tau = -2.9$ ps is used, the excitation becomes much more robust not just against changes in the pulse area, but also against errors in the two-photon resonance.

An in-depth investigation into inhomogeneous line broadening would need to be a subject for a different paper and we could not address it quickly.

Action taken: We added an investigation of the effect of spectral wandering to the Supplementary Section X.

Review comment 2.5: c) B_x and B_z should be connected to an explicit equation in the appendix. Additionally, would it not be more reasonable to connect B_x to an effective optical field?

All in all, this is nice work and the authors will be able to address these question in a convincing way.

Response:

The term "effective magnetic field" is conventionally used to express matrix elements of a 2x2 Hamiltonian in a way that facilitates interpretation of the dynamics, because the state vector on the Bloch sphere precesses around it. It is not to be confused with any real physical magnetic field and simply characterizes the effective driving in the more abstract

two-level system after obtaining the stroboscopic Hamiltonian and applying Schrieffer-Wolff transformation.

Action taken: We added the definition of B_x and B_z using an explicit equation in the appendix. We also rephrased the corresponding paragraph in the main text to clarify the nature of the effective magnetic field.

> **Main text:** Doing this and introducing the time dependence of the laser envelopes, $f \to f(t)$, we obtain the effective Hamiltonian $H^{\text{eff.}} = \frac{\hbar}{2}(B_x(t)\sigma_x + B_z(t)\sigma_z)$ with components $B_x(t)$ and $B_z(t)$ obtained by calculating the inner product
>
> $$B_{x/z}(t) = \frac{1}{\hbar}\mathrm{Tr}[\sigma_{x/z}H^{\text{eff.}}]. \tag{3}$$

Reviewer #3 :

This manuscript reports a novel excitation protocol–single-shot Floquet-engineered two-photon excitation (FTPE)–for robust biexciton preparation in a semiconductor quantum dot. By using two symmetrically detuned ultrafast pulses, the authors approximate an effective stroboscopic Hamiltonian that directly couples the ground and biexciton states. Both theoretical modeling including phonon interactions and convincing experimental demonstrations are presented, achieving biexciton preparation efficiencies exceeding 96% and polarization-entanglement fidelities of 93.4%. The work addresses challenges in quantum-dot–based entangled photon sources. The proposed approach is original, and experimentally executed. Overall, the manuscript is well within the scope of Nature Communications and represents a significant advance in quantum photonics and coherent control.

Response: We thank Reviewer #3 for the thoughtful and positive evaluation of our work. We are grateful for the reviewer's recognition that the proposed FTPE protocol is original, experimentally well implemented, and represents a significant advance in quantum photonics and coherent control.

We have carefully considered all of the reviewer's comments and suggestions, and have revised the manuscript accordingly. Below, we respond to each point in detail.

Review comment 3.1: That said, there are several important points that require clarification, strengthening, or deeper discussion, particularly regarding the theoretical assumptions, the generality of the protocol, and comparison with existing schemes.

The effective Hamiltonian relies on a higher order expansion in the 1/ delta, assuming detuning delta large. While this condition appears satisfied for the chosen parameters, the manuscript would benefit from a more explicit quantitative discussion of the regime of validity, especially since second- and third-order terms are said to be relevant. For example authors can clarify how close one can approach with smaller detunings before the effective two-level description breaks down.

Response:

Indeed, the limits of applicability of our scheme relies on assumptions on the parameters. First, we would like to point out that one has to distinguish the applicability of Floquet theory in general form the convergence of the Magnus expansion, and from the validity of an effective two-level description. While all are related to the magnitude of the detuning, the applicability of Floquet theory requires the pulse envelopes to vary more slowly than oscillations with frequency δ. The corresponding stroboscopic Floquet Hamiltonian can be calculated numerically without relying on Magnus expansion. However, low-order Magnus expansion leads to a clearer picture, since one obtains analytical expressions for the stroboscopic Hamiltonian. As can be seen in the second-order expression for the stroboscopic

Hamiltonian in the Methods section, higher-order corrections contribute as powers of $\frac{E_b}{\delta}$ and/or $\frac{f}{\delta}$, i.e., the detuning should be larger than both, the driving strength and the biexciton binding energy.
We compare in Fig. R10 the dynamics of the full Hamiltonian, the results obtained by numerically calculating the stroboscopic Floquet Hamiltonian, and second-order Magnus expansion. For these calculations, for which we used a pulse duration of $\tau_0 = 12.49$ ps, Floquet theory is found to apply for almost the full parameter space. As long as the detuning is larger than the biexciton binding energy $\delta \gtrsim E_b$, we find second-order Magnus expansion to be appropriate.

Regarding the reduction of the dynamics to a two-level system, our analysis based on Magnus expansion suggests that, in the stroboscopic picture, the exciton state occupation remains low if $\delta \gg E_b/\hbar$. Note that in our description, we also apply a Schrieffer-Wolff transformation to arrive at an effective two-level description where a residual mixing with exciton states is absorbed into the new basis states. This extends the range of applicability of a two-level description.

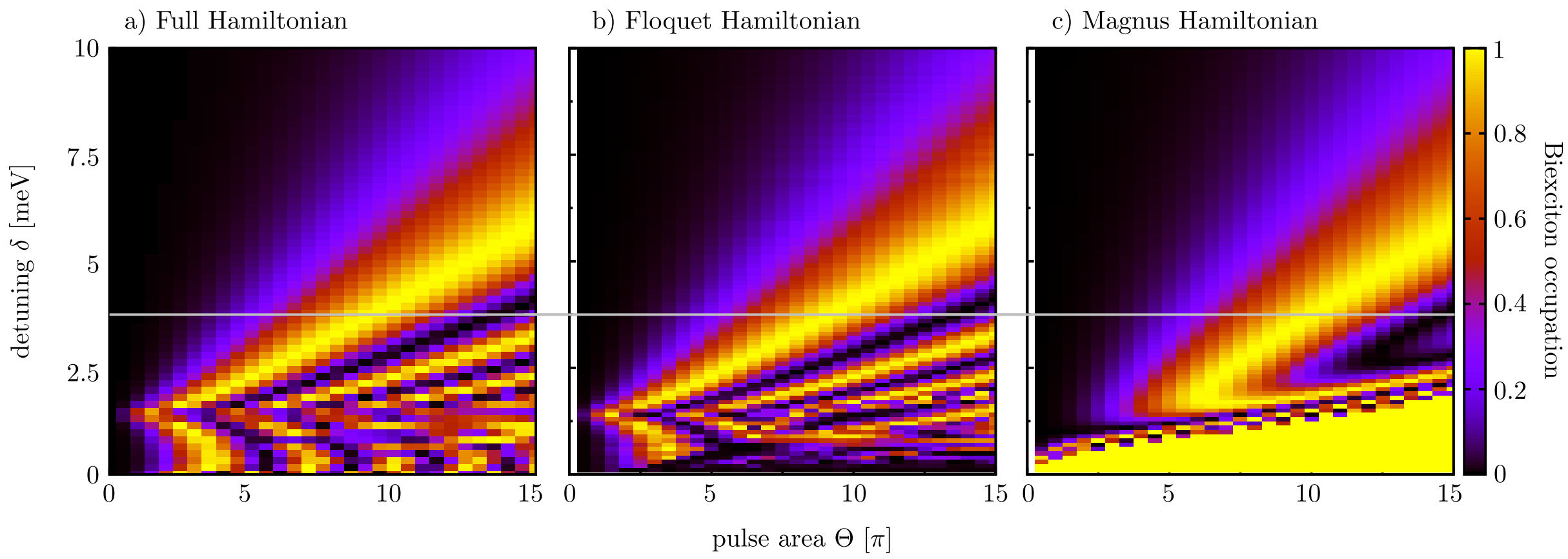

Figure R10: Biexciton occupation in its dependence on the pulse area and detuning for a) the full Hamiltonian, b) the numerically calculated Floquet Hamiltonian and c) the Magnus-expanded Hamiltonian, second order in $1/\delta$. The horizontal gray line indicates the δ used in the experiment. $\tau = 0$ ps and no phonons were used in these calculations.

Action taken: We added the investigation of the validity of our approximation to Supplementary Section VII.

Review comment 3.2: Although the exciton is claimed to remain only weakly populated, it still plays a crucial virtual role. The Schrieffer–Wolff elimination assumes a strong separation of energy scales. Include a quantitative bound (maximum transient exciton occupation) across the parameter space explored experimentally. Discuss whether residual exciton population could contribute to dephasing or timing jitter in other regimes.

Response:

We thank the reviewer for his question. We found that the exciton population remains negligible at stroboscopic times. However, during the fast oscillations in between stroboscopic times, sizable exciton occupations are observed, which can reach around 1/3 occupation for $\tau = 0$ ps. This can be seen in Fig. 2 of the main text and Fig. R11 in this response. As a consequence of this, it would not be sensible to apply the SW approximation to the original Hamiltonian directly, but it is admissible in the Floquet picture.
As the state of the system follows the stroboscopic dynamics, the impact of exciton population on the state preparation is very weak, but we agree that mixing between the exciton and biexciton states may have an impact on the emitted photon properties. Investigations of the entanglement of the emitted photons (see Fig. R7) indicate that the entanglement after FTPE is very similar to that after TPE, and hence, transient exciton occupations likely behave similar.

Fig. R12 shows the transient exciton population for the full model as well as the stroboscopic model. As we have seen before, the exciton is never meaningfully occupied in the stroboscopic model, which is also what enables the use of the Schrieffer-Wolff transformation of the Floquet Hamiltonian. For the full model, where the excitonic occupation oscillates over time, there are different regimes. For the one we primarily investigate in our work, which is at $\tau = -2.9$ ps and $\Theta \simeq 13\pi$, the exciton occupation is negligible.

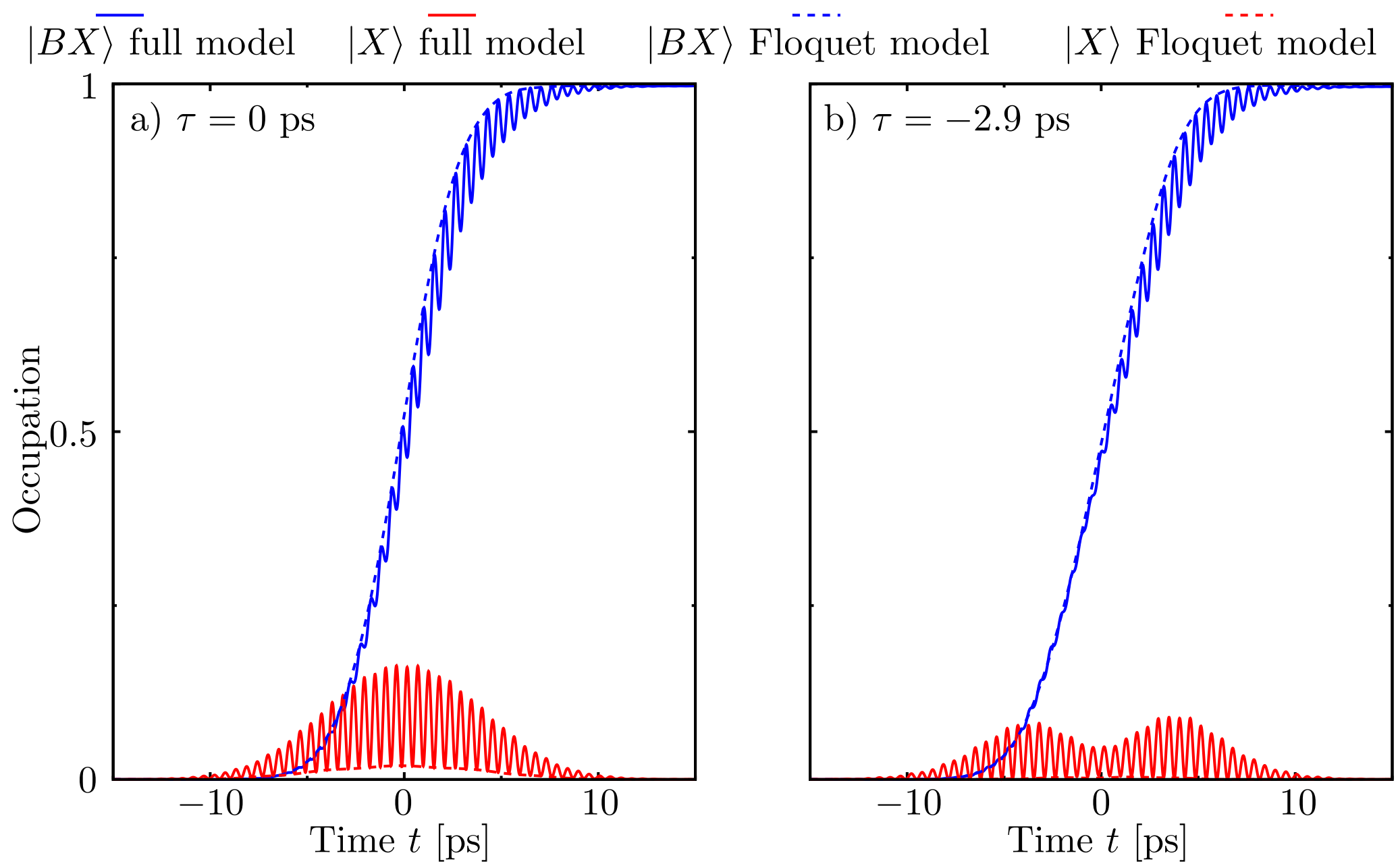

Figure R11: Comparison between the full model and the Floquet model. The Floquet Hamiltonian was calculated numerically and thus does not have any inaccuracies from the Magnus expansion. The exciton and biexciton occupations are displayed for a) no delay and b) a delay of −2.9 ps. These calculations were done without losses or phonons.

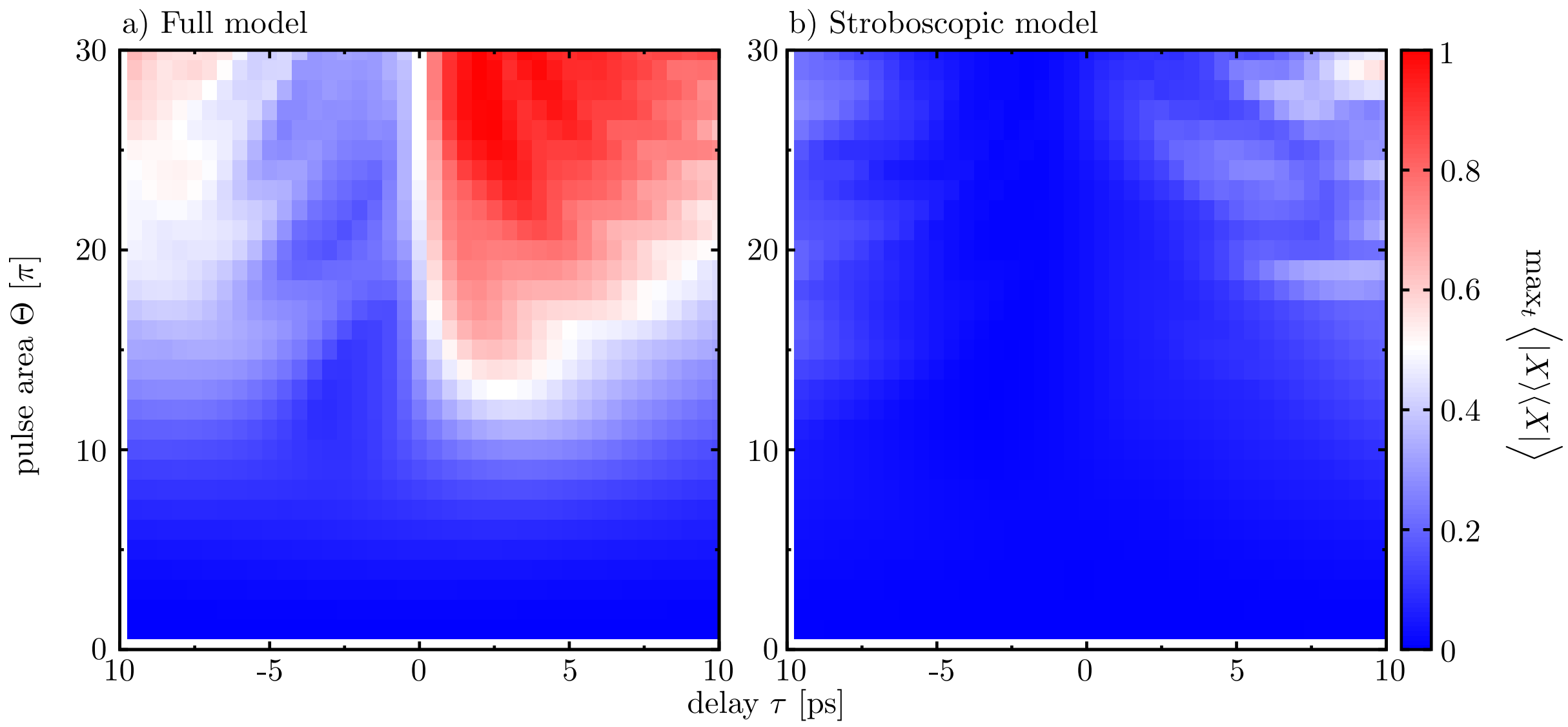

Figure R12: The maximal exciton occupation for a) all times and b) only for stroboscopic times in dependence on the pulse area and the delay. The calculations were done without phonons or losses.

Review comment 3.3: The manuscript states that FTPE is distinct from, phonon-assisted excitation, STIRAP, and SUPER, but the comparison remains largely qualitative. Clarifying explicitly in what sense FTPE offers a unique advantage rather than an incremental improvement, will be helpful.

Response:

We thank the reviewer for this comment. We emphasize the qualitative distinction to other protocols because FTPE is a fundamentally new approach to designing a class of excitation protocols. For practical relevance is that high excitation fidelity (comparable to TPE) is achieved while it is simultaneously much more robust against changes in the pulse area and it is experimentally easy to implement. Moreover, no polarization filtering is needed due to the large detunings involved. Also the entanglement fidelity lies in a similar range as those achievable using TPE. Being the first of its kind, our FTPE-based source is a prototype, which could be improved on in the future by combining it with other approaches, such as strongly coupling the QD to a microcavity.

Action taken: We have added a Table I. to compare the FTPE scheme with references in the literature.

Review comment 3.4: Authors claim that the fidelity is limited by re-excitation induced by the CW ac-Stark laser used to cancel FSS, rather than by FTPE itself. It weakens the claim of intrinsic robustness. A comment on whether FTPE could be combined with alternative FSS-removal techniques (e.g., strain or magnetic fields) and an estimate of the achievable fidelity in

the absence of CW-induced re-excitation, should be added.

Response: We thank the reviewer for this valuable point. The FTPE scheme is compatible with other FSS-erasure techniques, such as strain, electric-field, and magnetic-field tuning. These alternative approaches could remove the need for the additional CW ac-Stark laser and thereby avoid the associated re-excitation process. We have added a corresponding discussion in the revised manuscript to clarify the compatibility of the FTPE protocol with other FSS-erasure techniques.

In the revised Supplementary Section VIII, we have carried out simulations to estimate the achievable entanglement fidelity in the absence of CW-induced re-excitation, as shown in Fig. R13 f). The simulated fidelity in the absence of CW laser is 97.7%, which is limited by the ac-Stark effect induced by FTPE pulses itself.

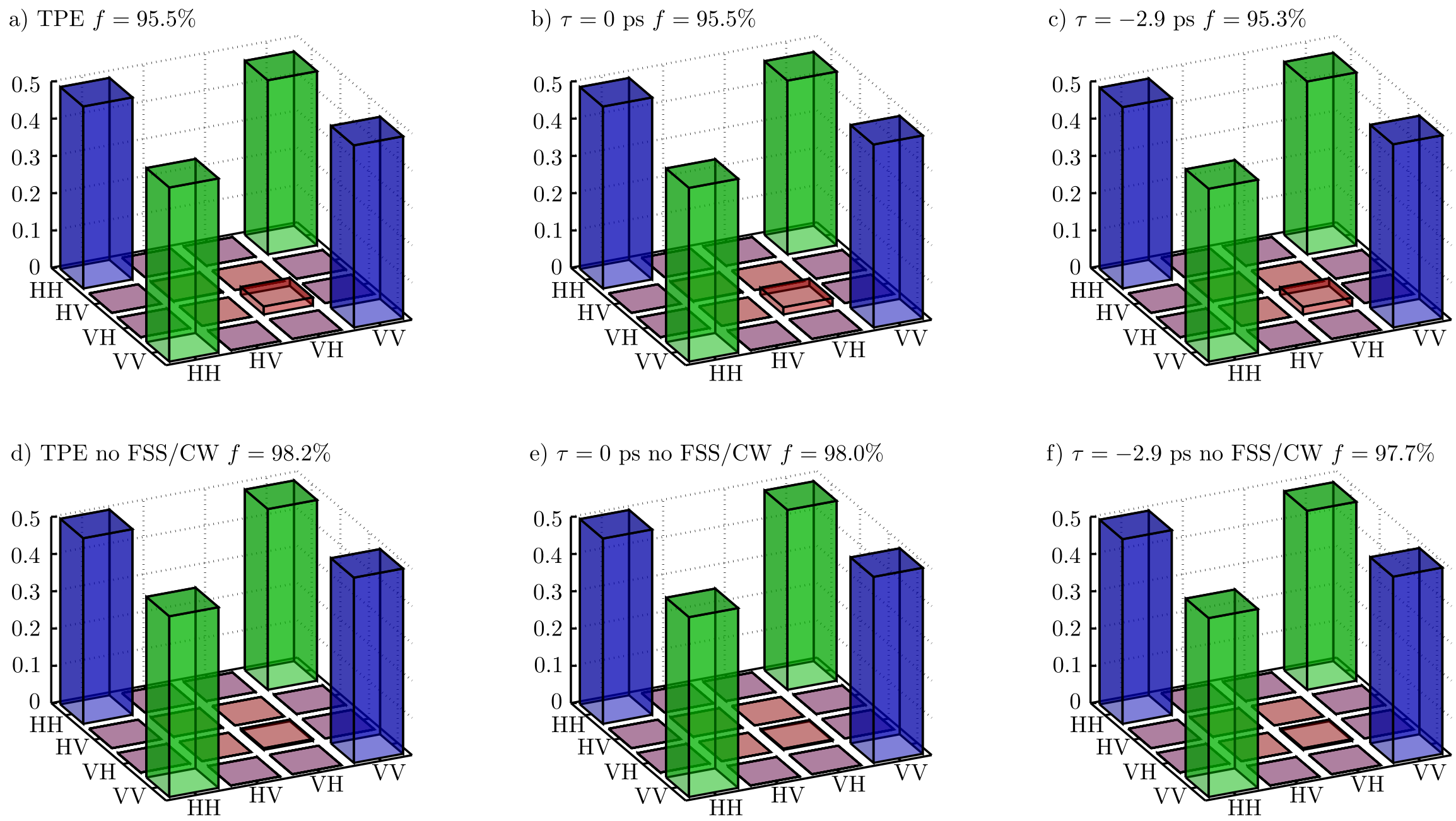

Figure R13: Entanglement fidelities and density matrices in the polarization basis for TPE, as well as FTPE with $\tau = 0$ ps and $\tau = -2.9$ ps. All calculations were done without phonons. a)-c) are calculated with FSS and the CW laser to compensate it and d)-f) are calculated without either.

Action taken: We have added a corresponding discussion in the revised manuscript to clarify that the compatibility of the FTPE protocol with other FSS-erasure techniques. We have also included simulation results estimating the achievable entanglement fidelity in the absence of CW-induced re-excitation in the Supplementary Information.

> **Main text:** The measured fidelity is close to our simulated value (95.3%) (Supplementary Section VIII), from which we attribute about 2.4% of the fidelity reduction to re-excitation induced by the CW laser. This effect is also supported by the power dependence of the autocorrelation measurements (see Supplementary Section III).

> The remaining reduction of about 2.3% arises from the intrinsic pulse-induced transient spectral shift during the FTPE driving itself, which is comparable with the TPE scheme. Importantly, the FTPE protocol is compatible with other established FSS-erasure techniques, such as strain [7], magnetic [53], and static electric-field [54] tuning, which could avoid the associated re-excitation and thereby enable higher entanglement fidelity. ...

Review comment 3.5: The conclusion suggests applicability to molecules, atoms, color centers, and superconducting qubits, but no concrete examples are discussed. Briefly outline what physical requirements (level structure, selection rules, achievable detunings) are essential for FTPE.

Response: We thank the reviewer for this important comment. We agree that the applicability to other physical platforms should be stated more concretely. In general, FTPE requires a suitable ladder-type or effective multilevel structure in which the initial and target states are coupled only indirectly via one or more intermediate states. In addition, the relevant optical selection rules must allow the two driving fields to couple the intermediate transitions, while the laser detunings must be large enough to suppress real population of the intermediate state but still permit an appreciable effective coupling between the initial and target states. Finally, the coherence time of the system should be sufficiently long compared with the single-shot driving window.

Beyond these general requirements, the detailed implementation will depend on the concrete level structure and selection rules of the specific platform. We have clarified this point in the revised conclusion.

Action taken: We have revised the conclusion to specify the physical requirements for extending FTPE to other systems, namely a suitable ladder-type structure, compatible selection rules, sufficiently large but experimentally accessible detunings, and coherence times long compared with the driving duration.

> **Main text:** ... Hence, the concept of all-optical single-shot Floquet driving provides a basis for developing platform-tailored excitation protocols for quantum emitters with suitable ladder-type structure, whenever one wants to drive transitions between states that are only indirectly coupled via off-resonant intermediate states. The essential requirements are compatible optical selection rules, sufficiently large but experimentally accessible detunings to suppress real occupation of the intermediate state while retaining effective coupling, and coherence times long compared with the single-shot driving window. Beyond the semiconductor QD system demonstrated here, this concept may also be applicable to selected molecular [56], atomic [57], colour-center [8], and superconducting artificial-atom systems [59].

Review comment 3.6: Minor Comments: Clarify the physical interpretation of the "effective magnetic field" analogy for readers less familiar with Floquet spin mappings.

Response: We thank the reviewer for this helpful suggestion. We have therefore rephrased the corresponding discussion around Eq. (2) in the main text to clarify that this is a formal analogy for the effective two-level dynamics in the reduced ground–biexciton subspace.

Action taken: We rephrased the corresponding text around Eq. (2) of the main text.

> **Main text:** ... This procedure yields a term that directly couples the ground to the biexciton state. The residual impact of the excitonic state is further eliminated by a Schrieffer-Wolff transformation, and we arrive at an effective Hamiltonian involving only ground and biexciton-like states with slowly varying driving envelopes. The details of this calculation can be found in the methods section. Formally, such a two-level system can be regarded as a spin precessing in a fictitious time-dependent effective magnetic field $B(t)$, which is described by the Hamiltonian
>
> $$H^{\text{eff.}}(t) = \frac{\hbar}{2}\big(B_x(t)\sigma_x + B_z(t)\sigma_z\big), \tag{4}$$

Review comment 3.7: Typographical issues are minor but should be corrected during revision e.g. occasional spacing and hyphenation inconsistencies.

Response: We thank the reviewer for pointing this out. We have carefully proofread the manuscript and corrected the typographical and formatting inconsistencies.

Action taken: Typographical and formatting issues have been corrected.

Reviewer #4 :

This manuscript proposes and experimentally demonstrates Floquet-engineered two-photon excitation (FTPE) as a robust protocol for biexciton preparation in semiconductor quantum dots, enabling high-fidelity entangled photon-pair generation. By employing symmetrically detuned dichromatic ultrafast pulses and interpreting the dynamics within a stroboscopic Floquet Hamiltonian framework, the authors report biexciton preparation efficiencies exceeding 96% and a polarisation-entanglement fidelity of 93.4%. The combination of rigorous theoretical modelling, including non-Markovian phonon simulations, and convincing experimental demonstrations represents a substantial and timely contribution to the field of solid-state quantum light sources. The work is well positioned with respect to existing excitation strategies, including resonant two-photon excitation, rapid adiabatic passage, phonon-assisted excitation, and SUPER-like dichromatic schemes ("red–red", "red–blue", and "blue–blue"). Subject to clarification of the points below, the manuscript might be suitable for publication in Nature Communications.

Response: We thank Reviewer #4 for the positive assessment of our work and for the constructive comments. We appreciate the reviewer's recognition that the combination of rigorous theoretical modelling and convincing experimental demonstrations makes this work a timely contribution to the field of solid-state quantum light sources.

We have carefully addressed the points raised by the reviewer and revised the manuscript accordingly. Our point-by-point responses are given below.

Review comment 4.1: Major points: The two pulses used in FTPE appear formally identical to those employed in the "red–blue" SUPER-like excitation protocol (Refs.S8, S9, S10, S11). This raises the question of whether FTPE and SUPER represent equivalent physical mechanisms described in different theoretical languages, or whether they operate in genuinely distinct regimes. The authors are encouraged to explicitly clarify the conceptual and operational differences between FTPE and SUPER.

Response:

We would like to point out that a discussion on the distinction to SUPER is given in the Supplementary Section XI D. In short, the working principles are quite different. In SUPER, one laser effectively dresses the two-level scheme. The second laser drives coherent transitions in the laser-dressed bases with modified energies. In contrast, laser dressing and Stark shifts are not relevant in FTPE, and also the resonance conditions are quite different. Several articles discuss dichromatic excitation with red and blue detuned pulses, which however can also be understood by SUPER as they also involve coherent excitation between laser-dressed states. In FTPE, on the other hand, the concept of laser-dressed state becomes ill-defined as no single rotating frame exists that suppresses fast oscillations.

Moreover, in tests where we applied Floquet theory to SUPER, we find that low-order Magnus expansion does not yield a sensible description for parameters of the SUPER scheme. Finally, SUPER is designed to drive transitions in a two-level system, while FTPE drives a transition between two states in a three-level system.

Action taken: We have changed the Supplementary Section title to explicitly contain the word "SUPER". We also added the clarification that other dichromatic excitation schemes using red and blue detuned pulses are of the SUPER-type rather than FTPE-like.

> **Supplementary Information:** SUPER and dichromatic excitation
> ... FTPE has in common with SUPER mainly the fact that two-colour off-resonant excitation is employed. But in contrast to SUPER, Stark shifts do not set the resonance condition or constitute the working mechanism of FTPE. For the same reason, FTPE also differs from dichromatic excitation with one red- and one blue-detuned laser [S12].

Review comment 4.2: When discussing the detuning conditions, the manuscript frequently references the biexciton binding energy. However, since FTPE can also be applied to exciton preparation, it would seem more natural to compare the detuning scale to the exciton radiative linewidth (or decay rate). The authors are encouraged to clarify which energy scale is most relevant for assessing the operational regime of FTPE and to justify their choice.

Response: We thank the reviewer for this comment. The goal of FTPE is to introduce unconventional terms into an effective stroboscopic Hamiltonian that do not exist in the original Hamiltonian, here, direct transition matrix elements between the ground and the biexciton state. One could certainly investigate a stroboscopic Hamiltonian for a two-level system comprised of only ground and exciton state, but since for this systems the transitions can already be well controlled using an appropriately chosen laser pulse, the benefit of FTPE for a two-level system is unclear.
In order to apply the Magnus expansion, the detuning δ has to be the largest energy scale in the Hamiltonian. As typically the biexciton energy E_b is larger than the driving f, the condition $\delta \gtrsim E_b$ is the more relevant bound. Radiative line broadenings are orders of magnitude smaller and thus no issue. The non-Markovian phonon bath for QDs typically features sidebands with strongest coupling at a frequency of about 2 meV, which is roughly in the same order of magnitude as the biexciton binding energy. Choosing larger values of δ thus ensures both, the working principle of FTPE, which requires $\delta \gtrsim E_b$ and reduced phonon-assisted leakage into the exciton state (see Fig. R15).

Action taken: We added the clarification that δ has to be the largest energy scale in the system for the Magnus expansion to converge.

Main text: ... For detunings δ large enough so that $T \ll \tau_0$, fast oscillations can be eliminated and H is replaced by a stroboscopic Hamiltonian $H^{\text{eff.}}$. If moreover δ is the dominant energy scale in the Hamiltonian, $\delta \gg E_b/\hbar, f$, the effective Hamiltonian $H^{\text{eff.}}$ can be obtained by systematic expansion in $1/\delta$ [48,49]. ...

Review comment 4.3: Are the two pulses in FTPE required to have equal peak intensities? If so, is this a strict requirement or merely an optimal condition? A brief discussion of the tolerance to intensity imbalance, both theoretically and experimentally, would be valuable.

Response: We thank the reviewer for this question. Equal peak intensities are not a strict requirement of FTPE, but they represent the most natural and typically near-optimal symmetric operating condition for the protocol. When the two pulse amplitudes are imbalanced, the effective coupling and the associated stroboscopic dynamics become asymmetric, which generally shifts the optimal working point and can shift the robustness window.

In Fig. R14, we present simulations of the preparation fidelity when varying the driving strengths of red and blue detuned pulses independently of each other. We find a highly symmetric structure, indicating that the most desirable protocols are likely those with equal driving strengths. In the main text, we identified particular robustness of high population fidelity with respect to the change of driving strengths of both pulses simultaneously when a finite time delay is used. Here, our simulations indicate robustness for finite delays even when the driving strength of the individual pulse is varied.

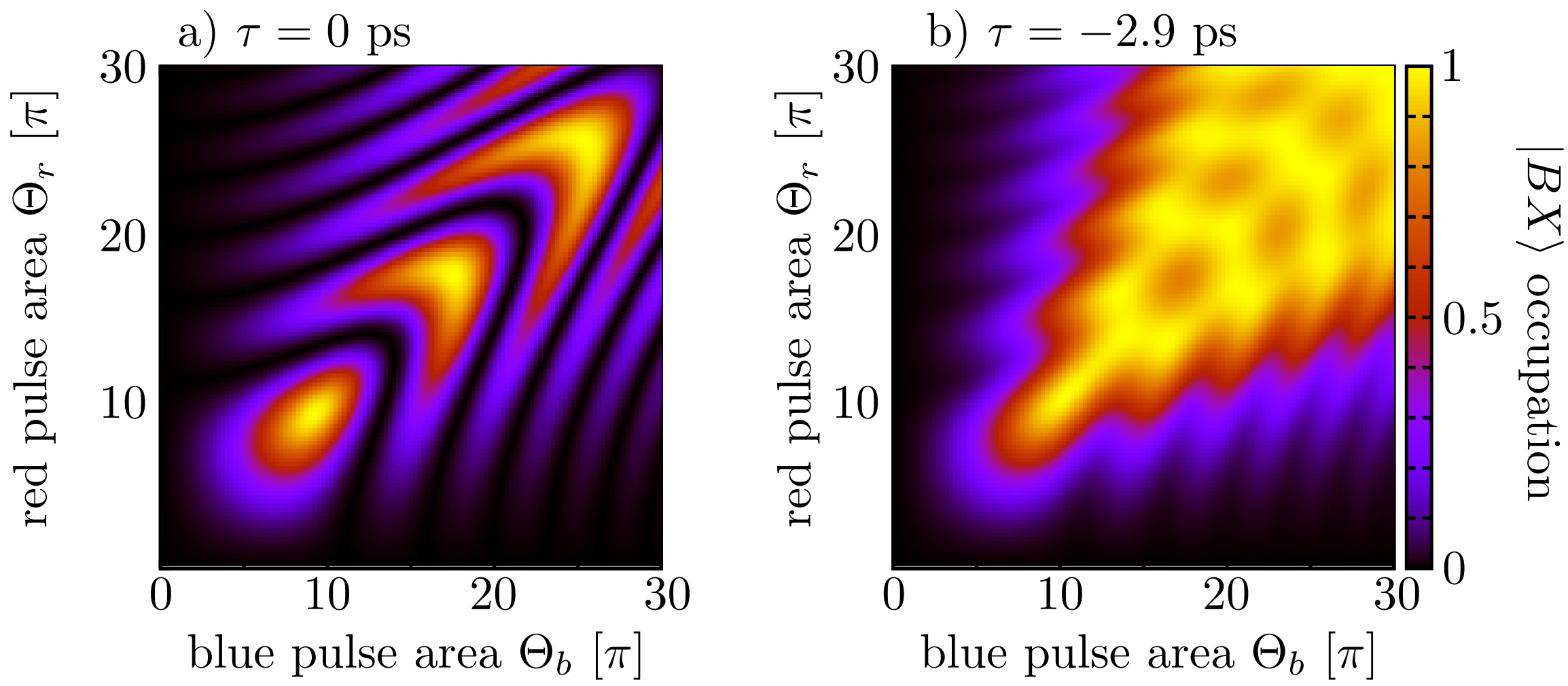

Figure R14: Biexciton occupation after the FTPE excitation with $\tau = 0$ ps (a) and $\tau = -2.9$ ps (b). The calculations were done without phonons or losses.

Review comment 4.4: Unlike red-detuned excitation, blue-detuned laser pulses can induce phonon-assisted population of exciton and biexciton states. How does such phonon-assisted excitation influence FTPE dynamics? In addition, since both FTPE and SUPER-like protocols

inevitably populate the exciton state during excitation, the authors should clarify how residual exciton population affects the biexciton–exciton cascade and whether this has any measurable impact on entanglement generation or fidelity.

Response: Fig. R15 shows heatmaps of the exciton population after the FTPE pulses passed. When phonons are not taken into account, the exciton only retains excitation for a range of detunings $\hbar\delta \in [-E_b/2, E_b/2]$, where both lasers are energetically below the exciton. For $\hbar\delta = E_b/2$, the blue laser is resonant to the exciton and thus, we observe Rabi rotations. For larger δ, the phonon-free case in Fig. R15 a) does not show any excitation. When phonons are included in Fig. R15 b), we find phonon-assisted exciton population, reflecting the fact that the phonon spectral density has a maximum at about 2 meV. Phonon-assisted excitation becomes negligible at larger values for δ, in particular for the one we use in the main text and in the experiments, which is represented by the gray line in the figure.
We note that in Fig. 3a in the main text, detrimental phonon effects for biexciton state preparation can be seen for detunings below ~ 3 meV. The origin is precisely the phonon-assisted population of the exciton state.

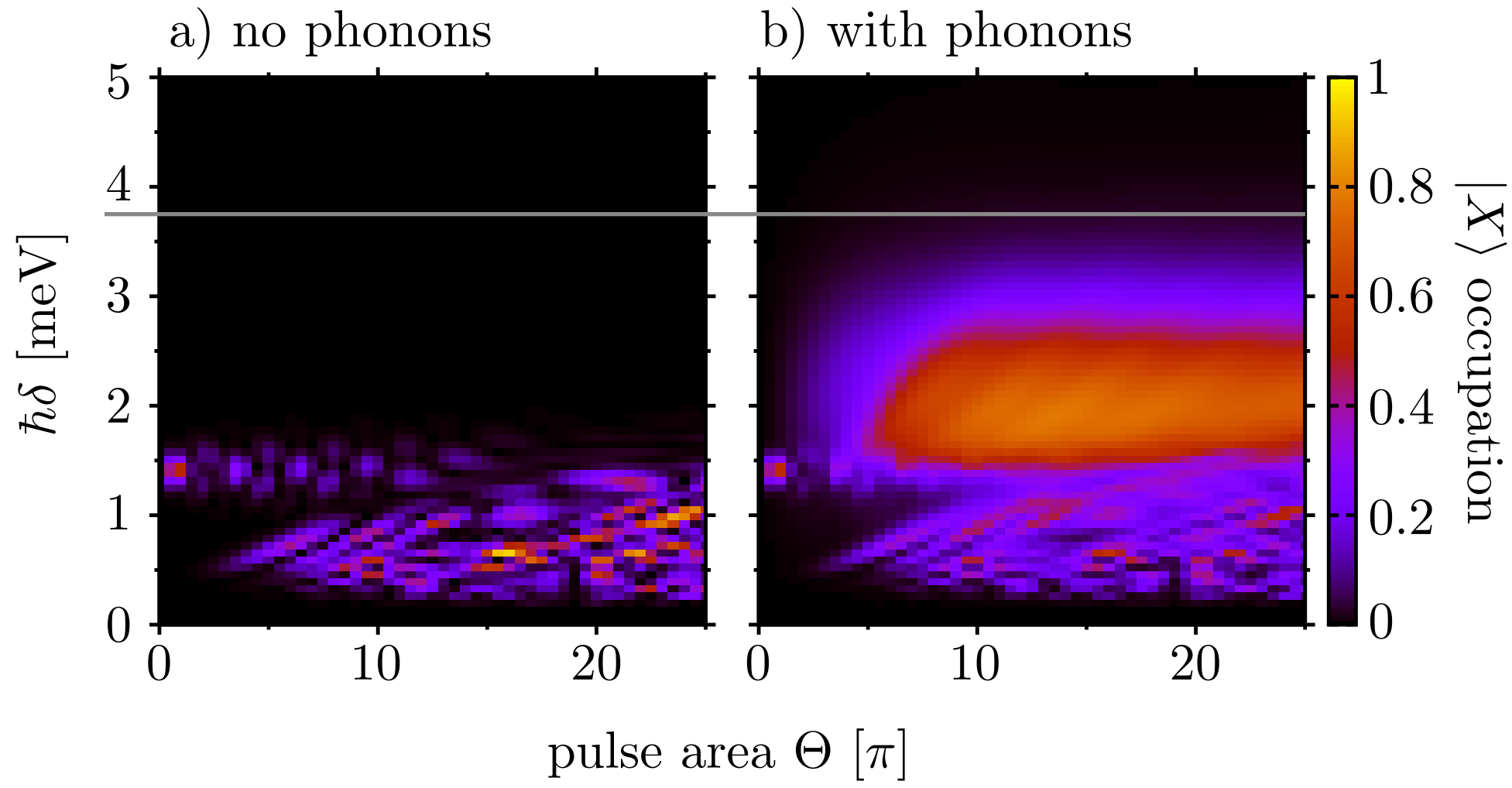

Figure R15: The heatmaps represent the exciton occupation after the FTPE pulses passed for different pulse areas ($\Theta_b = \Theta_r$) and detunings δ from the TPE frame without (a) and with (b) phonons. To avoid any non-FTPE or phonon-assisted excitation of the exciton, the spontaneous decay via the rate γ was turned off. The gray line represents the δ-value chosen in the experiments and the main manuscript and is equal to 3.75 meV. For these calculations, we used $\tau = -2.9$ ps.

Action taken: We added a discussion of phonon-assisted exciton population in the discussion of Fig. 3(a).

> ... The inset of Fig. 3(a) shows calculated results without phonon effects, confirming that phonon assistance is not essential for BX preparation. We attribute detrimental

effects due to phonon coupling to phonon-assisted population of the exciton state, which are significant for detunings below about 3 meV, where the coupling to phonons is strongest. Choosing a larger detuning is therefore advisable to decouple from phonon effects. Without phonons, the reachable biexciton occupations are even higher, making the scheme even more attractive for emitters not affected by phonons, such as atomic systems. ...

Review comment 4.5: The pulse delay is a key control parameter in FTPE. What physical factors determine the optimal delay values (e.g., detuning, pulse duration, amplitude)? If possible, the authors are encouraged to provide:an analytical expression, or a schematic or supplementary figure illustrating how the optimal delay depends on system parameters.

Response: Qualitatively, robust excitation is indicated by a large patch in parameter space where near-unity state preparation fidelity is observed. How exactly to quantify the precise optimum is not a well-defined question as there may be a trade-off between the size of the patch and the admissible distance to unity state preparation fidelity. Moreover, even using Magnus expansion, the expressions for finite detuning are complicated and depend on many parameters. This makes providing an analytical expression extremely difficult.

Action taken: In order to give a visual impression about the robustness for different delay times, we provide an additional figure in the Supplementary Section IX showing color plots of population for various values of τ.

Review comment 4.6: As the biexciton population is a central metric for FTPE performance, more experimental detail is required. The authors should clarify how the biexciton population is extracted from the measured signals, how calibration and normalization are performed, and what assumptions enter the conversion from photon counts to population.

Response: We thank the reviewer for this important comment. Since the biexciton population is a central metric for evaluating FTPE performance, we agree that the calibration and normalization procedure should be described more explicitly.

Experimentally, the BX occupation is calibrated by fitting the experimental TPE map to the corresponding simulated one using two scaling factors along the horizontal and vertical axes, as shown in Fig. R16. The vertical scaling factor establishes the proportionality between the measured BX emission intensity and the corresponding final BX population in the simulation, while the horizontal scaling factor relates the experimentally controlled pulse amplitude to the pulse area used in the model.

This conversion relies on the following assumptions: first, within the reference TPE dataset, the integrated BX emission intensity is proportional to the final BX population under fixed

collection and detection conditions; second, the experimental pulse amplitude is related to the simulated pulse area by a single global scaling factor; and third, once this calibration is established from TPE, the same normalization can be consistently applied to the FTPE data, allowing a direct comparison between experiment and theory on the same scale.

For clarity, we have added a brief explanation in the main text and included a dedicated section in the Supplementary Information describing in detail how the BX population is extracted, calibrated, and normalized.

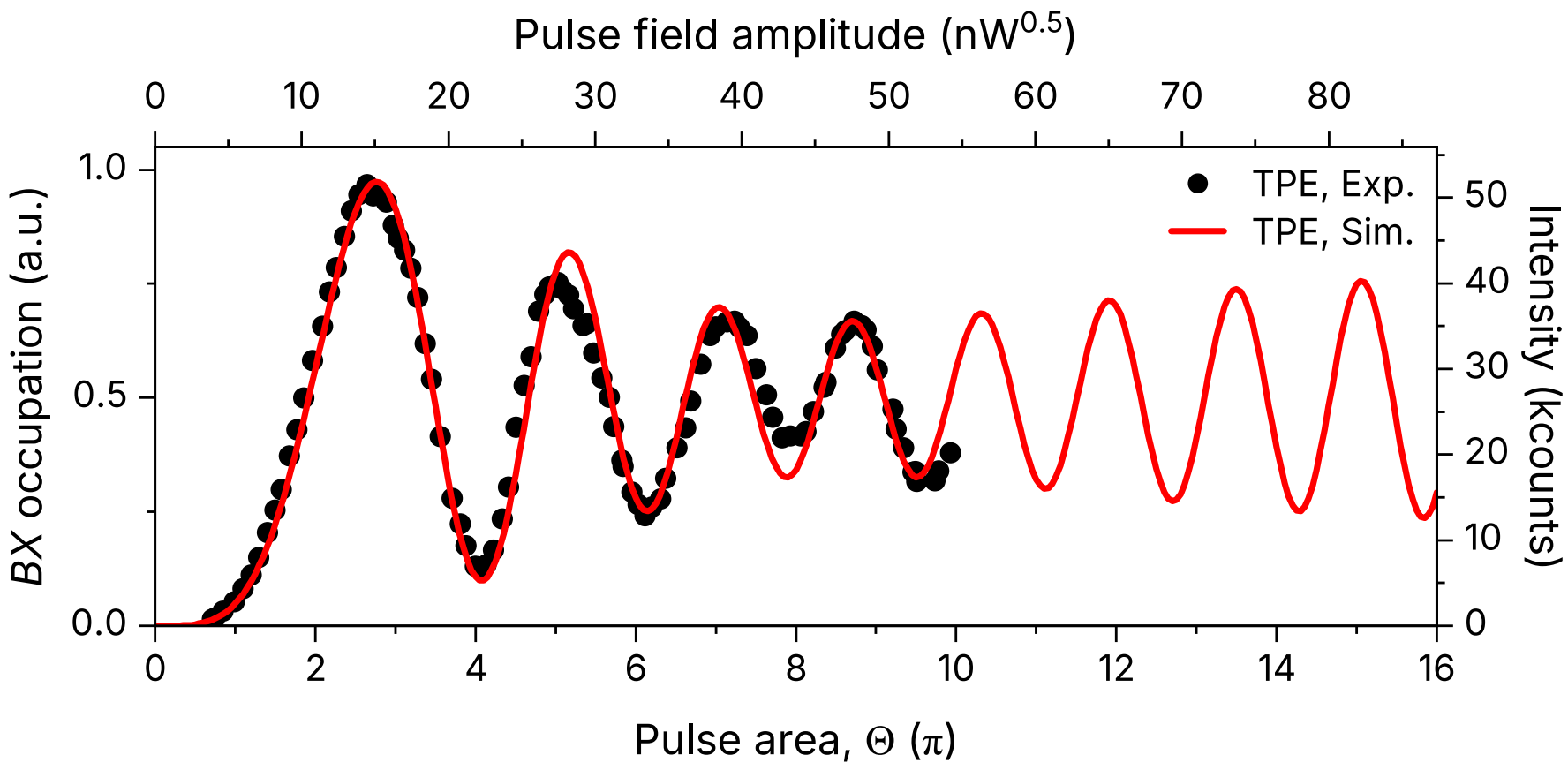

Figure R16: Experimental TPE Rabi oscillation data are fitted to the simulated BX occupation as a function of pulse area. The fitting procedure determines the conversion factor from measured intensity to BX occupation, the calibration factor from pulse field amplitude to pulse area, and the radius of the electronic wavefunction.

Action taken: We have included a clarifying statement in the main text explaining how the occupation probability is extracted from the experimental data, and we have added a section in the Supplementary Information describing the calibration and normalization procedure in detail.

> **Main text:** ... The experimentally achieved occupations and pulse area are calibrated by fitting the TPE Rabi-oscillation data to numerical results with two scaling factors, as shown in Supplementary Fig. 4. ...
>
> **Supplementary Information:** ... This calibration procedure relies on the following assumptions. First, within the TPE reference dataset, the integrated emission intensity is proportional to the final occupation under fixed collection and detection conditions. Second, the experimentally controlled pulse amplitude is related to the pulse area used in the simulation by a single global scaling factor. Third, once the calibration is established from the TPE dataset, the same normalization can be consistently applied to the FTPE measurements, allowing direct comparison between experiment and theory on the same scale. ...

Review comment 4.7: The authors state that phonon effects play only a minor role in FTPE. In contrast, SUPER requires operation in the phonon-decoupling regime to reach its full potential. A clear physical explanation for this difference would significantly strengthen the manuscript and help position FTPE relative to existing off-resonant excitation schemes.

Response: See our response to Question 4.4. We indeed also use a detuning large enough to decouple from phonons. This is in line with the basic requirements for FTPE, which also requires the detuning to be the largest energy scale in the system.

Review comment 4.8: Minor points: Can FTPE operate efficiently in quantum dots with small or negative biexciton binding energy? A brief discussion would be helpful.

Response: We thank the reviewer for this question. For small biexciton binding energy, the resonance condition for two-photon excitation would also make single-photon transitions resonant. Hence, the exciton state can no longer be decoupled from the dynamics of ground and biexciton states.
FTPE works almost the same when the biexciton binding energy is negative. However, phonon-assisted exciton population is suppressed at low temperatures, because scattering from the biexciton to the exciton requires the absorption of energy from phonons in the environment.

Action taken: We added a brief discussion in the revised in discussion section to clarify the behavior of FTPE for negative and small biexciton binding energy.

> ... FTPE works almost the same when the binding energy is negative. As binding energy decreases, more laser power is needed and for zero binding energy no robust excitation can be obtained. ...

Review comment 4.9: Reference 32 appears to be incomplete and should be corrected.

Response: We thank the reviewer for pointing this out. The reference list has been corrected.

Action taken: Reference 32 has been completed.

Review comment 4.10: The acronym STIRAP should be defined upon first appearance in the main text.

Response: We thank the reviewer for this comment. We have now defined STIRAP (stimulated Raman adiabatic passage) upon first appearance in the main text.

Action taken: The acronym STIRAP has been defined upon first use.

> **Main text:** ... The optical selection rules differ from stimulated Raman adiabatic passage (STIRAP), and the resonance conditions of FTPE differ from those in other two-colour excitation schemes such as SUPER. ...

Review comment 4.11: In Fig. 1c and 1e, the laser excitation spectra exhibit three peaks. Are these features due to spectral interference between the two pulses? Clarification would be useful.

Response: We thank the reviewer for this comment. We believe that the ripple structures in the excitation spectra of Fig. 1c and 1e do not result from spectral interference between the two excitation pulses, since their center frequencies are well separated and they have no appreciable spectral overlap. Instead, the ripples most likely originate from residual spectral modulation introduced by the optical path, particularly the cross-polarization laser-filtering optics. In such a filtering scheme, weak birefringence, wavelength-dependent retardance, and residual multiple reflections in polarization optics can be converted into an oscillatory extinction/transmission spectrum for broadband pulses. This type of polarization-dependent spectral fringe has been discussed for wave plates or retarders placed between crossed polarizers in Ref. [Astronomy & Astrophysics, 434(1), 377 (2005)], and similar residual laser-background fringes have also been reported in polarization-based dark-field filtering, as shown in the Supplemental Material of Ref. [Phys. Rev. B 95, 241306(R)]. To avoid possible confusion, we have clarified this point in the revised main text.

Action taken: We have added a description in the main text clarifying that the ripple structures in the excitation spectra are most likely caused by the wavelength-dependent transmission and extinction characteristics of the cross-polarization filtering optics.

> **Main text:** ... Some ripples in the excitation pulse spectra are attributed to the polarization optics used in the setup. ...

Review comment 4.12: In the caption of Fig. 2e and 2f, the condition $\tau = 0$ is missing.

Response: We thank the reviewer for catching this omission. The figure caption has been corrected.

Action taken: The caption of Fig. 2 has been revised to include the missing contents.

Review comment 4.13: The curve shown in Fig. 4g appears not to be fully measured.

Response: We thank the reviewer for pointing this out. In the revised version, we have completed this measurement. Because the experimental conditions changed between the initial submission and the revised measurements, to ensure overall consistency across the

revised figures, we also repeated the relevant datasets in Fig. 3 and Fig. 4. The updated data are now complete, and the conclusions remain unchanged compared with the original version.

Action taken: We have replaced the incomplete dataset in Fig. 4g with a complete measurement and updated the relevant datasets in Fig. 3 and Fig. 4 to ensure consistency under the same experimental conditions. The revised figures now contain complete data and support the same conclusions as before.

Review comment 4.14: In Fig. 3b, the amplitude of the Rabi oscillations appears to increase with driving strength. A brief explanation would be appreciated.

Response: The non-monotonic reappearance behaviour of the Rabi oscillation amplitude can be attributed to dynamical decoupling. In short, stronger driving (larger Rabi splitting) probes the phonon spectral density at larger frequencies. If the driving exceeds the maximum of the phonon spectral density at about 2 meV, the efficacy of phonon-induced dephasing decreases, and one dynamically decouples from phonons. For a two-level system, this has been predicted theoretically in [Phys. Rev. Lett. 98, 227403 (2007)] and experimentally verified in [Phys. Rev. Lett. 135, 263602 (2025)]. While Rabi reappearance for TPE is indeed interesting, this effect concerns the conventional TPE reference curve rather than the FTPE mechanism; we have kept the main-text discussion focused on FTPE and only added this explanation in the response letter.

Review comment 4.15: In Figs. 3b,c and 4b,c, the x-axes use different parameterizations (pulse area vs laser amplitude). For clarity and consistency, the authors may consider unifying the x-axis definitions.

Response: We thank the reviewer for this suggestion. In the revised manuscript, we have unified the x-axes of both the experimental and theoretical panels using the "per-pulse area" as the common parameter. In addition, for the experimental data, we have also included a top x-axis indicating the corresponding per-pulse amplitude used in the measurements, as shown in Fig. R17.

Action taken: We have revised Figs. 3 and 4 so that both the experimental and theoretical x-axes are consistently presented in terms of "per-pulse area". For the experimental panels, we have also added a top axis showing the corresponding per-pulse amplitude used in the measurements.

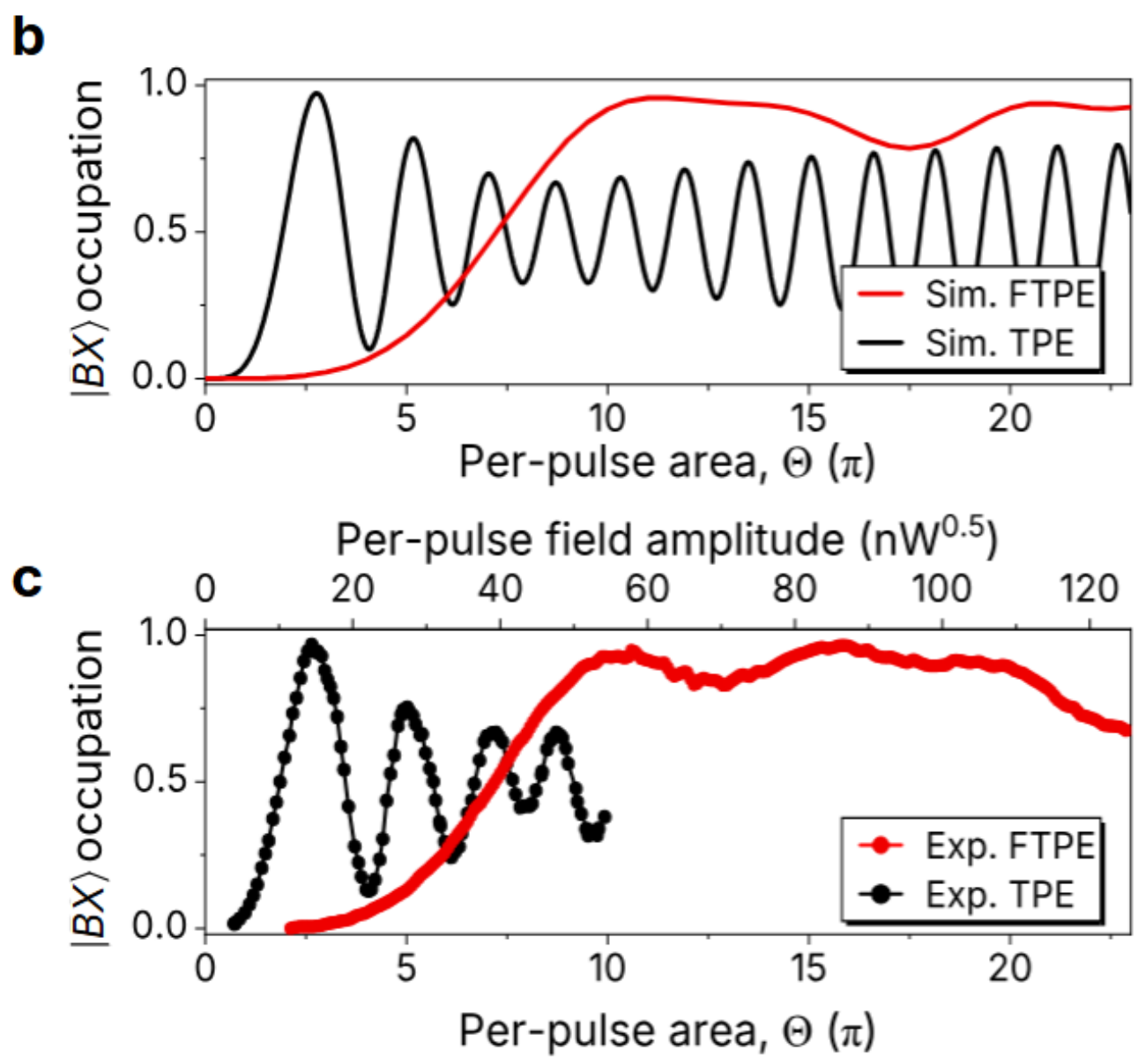

Figure R17: The revised Fig. 3(b) and (c).

Response letter

Reviewer #1 :

The manuscript "Robust entangled photon generation enabled by single-shot Floquet driving" by Yan et al. improved a lot in the review. Especially the supplemental, which is now showing really a lot of useful additional facts on the presented scheme, which helps a better understanding for the reader. I still think the manuscript is extremely interesting as it presents a novel excitation scheme, but some points, especially on the claims of robustness seem to be worse than initially presented. I have some additional comments, that I think should be addressed before a publication of the manuscript:

Response: We thank the reviewer for the careful assessment of our revised manuscript and for recognizing the novelty and interest of the work. We have carefully considered the remaining concerns and further clarified the applicability of the Floquet description and the precise meaning of robustness.

Review comment 1.1: ad Review comment 1.1: I want to bring this up again, as the claimed Floquet-regime is one of the central novelties of the manuscript. From a certain perspective, one could write the entire paper without ever invoking the word Floquet, and instead speak about a high-frequency averaging yielding an effective Hamiltonian. On the other hand, the conscious use of the wording Floquet might in fact help the community to spot the limit of applicability of the approach laid out in the manuscript. Here, the large envelope includes more than 10 oscillations, so that a Floquet setup at inverse time scales smaller than the envelope and bigger than the individual oscillations might be justified. It would certainly not be justified with significantly less oscillations under the envelope, and the lack of generality of the approach should be stressed clearly in the manuscript.

Response: Indeed, in order to make use of the stroboscopic Floquet Hamiltonian, we require the changes in the pulse envelopes to be longer than the oscillation period $T = 2\pi/\delta$. We address this point together with other comments raised by the reviewer in a new paragraph in the section “Robust biexciton preparation using FTPE” in the main text.

Action taken: In the newly added paragraph, we now mention explicitly the findings in Supplementary Section VII and Supplementary Fig.6:

> ... The stroboscopic Floquet Hamiltonian describes the dynamics appropriately, down to small detunings where the pulse envelope no longer changes slowly with respect to $1/\delta$ (see Supplementary Section VII).

Review comment 1.2: ad Review comment 1.3: My initial comment that it is not surprising that excitation of a spin 0 state works with two photons has not been addressed. The authors worked out differences to other existing schemes, like to SUPER and to dichromatic excitation of a spin 1 state. However, that the scheme at hand can be viewed (or maybe not) as the detuned case of TPE should be addressed or clarified.

Response: We would like to point out that the generic behaviour of "detuned TPE" is visible in Fig. 3a in the regime for detunings below 1 meV: Sizable population inversion is possible at certain parameter values, but these come in small, seemingly random speckles. Due to the acute sensitivity to parameter fluctuations "detuned TPE" at small detunings is impractical for real-world application. By contrast, the high-frequency regime of FTPE is qualitatively different as it allows near-unity population inversion for large patches in the parameter regime. It is this robustness of FTPE, especially compared to typical behaviour of weakly detuned TPE, which makes it useful and practicable.

Action taken: In the newly added paragraph we now emphasize that qualitatively different regimes are observed when the detuning is varied from weak and perturbative to strong.

> ... Qualitatively different parameter regimes can be identified: For TPE with small detunings, biexciton population is found at certain spots but the results are very sensitive to parameter fluctuations. At larger detunings, cusps at $\hbar\delta = E_b/2$ indicate the simultaneous resonance of the lasers with the $|0\rangle$ to $|X_V\rangle$ and $|X_V\rangle$ to $|BX\rangle$ transitions. Increasing the detuning further to about $\hbar\delta \approx E_b$, one reaches the FTPE regime. We note that low-order Magnus expansion captures only the FTPE regime well. The stroboscopic Floquet Hamiltonian describes the dynamics appropriately, down to small detunings where the pulse envelope no longer changes slowly with respect to $1/\delta$ (see Supplementary Section VII). Notably, TPE theory [12] involves an adiabatic approximation that breaks down for the large detunings required for FTPE (see Supplementary Section X A).

Review comment 1.3: ad Reviewer comment 1.7: To me it is unclear whether between the 3 presented cases in Figure R2 is any fundamental difference in theory, i.e. if there is an abrupt regime change to the FTPE regime somewhere after the double resonant excitation case, or even before, or if there is a smooth transition between the 3 presented cases. This is connected to the newly presented Figure S6 and Reviewer comment 3.1. To me it is still unclear at which point the Magnus-expanded Hamiltonian is not valid any more and whether all 3 presented cases can be described with the FTPE model or if one needs different models for different detunings? I am asking this, because to me all 3 presented cases in Figure R2 are the same thing, only with different detuning but not fundamentally different. As the main selling point of the paper is to present a new scheme, this should be clarified.

Response: As far as the experimental realization is concerned, all three cases indeed are implemented in a similar way as they require one or two lasers that fulfill the two-photon resonance conditions. Yet the physical situation is qualitatively different, as discussed in response to the last comment and in the newly added paragraph. The single-photon resonance is clearly a well defined and prominent point in parameter space. The transition to the other regimes is gradual and smooth.

As the Reviewer pointed out earlier, the Floquet description requires the detuning to be larger than the time scale of changes in the laser envelopes. For single-shot, i.e. pulsed, excitation, this naturally leads to a lower bound of δ to which Floquet theory is valid, as can be seen by comparing the Floquet result in Supplementary Fig.6b with the full numerical calculation in Supplementary Fig.6a. Thus, Floquet theory is **not** applicable to the case of TPE. Conversely, as discussed in detail in the Supplementary Section X A, the standard treatment of TPE (see Ref. [12]) involves an adiabatic approximation that breaks down with increasing detuning and thus does not cover the FTPE regime.

Magnus expansion is a high-frequency expansion, here, in f/δ as well as E_b/δ, and yields an approximation to the stroboscopic Floquet Hamiltonian. Magnus expansion is thus more limited than the stroboscopic Floquet description, as seen by comparing Supplementary Fig.6c to Supplementary Fig.6b. However, it yields easily interpretable analytical expression [e.g. Eq.(7) in the Methods Section] in the regime of most interest to us, namely the far-detuned regime where the single-exciton state can be eliminated.

Action taken: See Review comment 1.2.

Review comment 1.4: ad Reviewer comment 1.9: In their new text the authors write about robustness of the FTPE scheme with respect to experimental fluctuations. I think in light of Figure S5, this sentence should be modified to robustness against power fluctuations and quantified, i.e. how much fluctuation can be accepted to be e.g. within 1% of the targeted BX occupation. Furthermore, given the result from Figure R14, as it seems that the power fluctuations have to be in both lasers simultaneously. If only one of the colors fluctuates, then the plateau is left rather fast, so the scheme is maybe not as robust as it is claimed.

Response: In fact, as can be seen in S.Fig. 11b (formerly R14), FTPE with time delay (and only for this we claim robustness) is robust against fluctuation of one or both laser driving strengths. Quantifying the robustness with a single number is difficult because of the complexity of the landscape of population inversion versus driving strengths. However, a useful visual representation of the robustness against intensity fluctuations is given by contour lines corresponding to 80% and 95% of biexciton population, which we have added to S.Fig. 10 and 11. The area enclosed by the contour line in S.Fig.11b clearly shows that efficient population inversion is found in large parts of the parameter space. This is true for

variations of one or both laser intensities simultaneously. We also note that the robustness also extends to other parameter fluctuations, such as the departure for the exact two-photon resonance, which is shown in S.Fig.10, and which is particularly interesting in the presence of spectral wandering of QDs.

In order to further alleviate the Reviewer's concerns regarding the robustness when only one pulse area is increased, we now show additional panels in S.Fig.11 corresponding to cross sections through the colour plots. Specifically, for delay $\tau = -2.9$ ps and $\Theta_r = 25\pi$ we find that the area of the blue-detuned pulse can be varied in a range spanning 29π and still yield biexciton populations exceeding 80%. These results show that the robustness of FTPE is not restricted to two pulse strengths varying simultaneously, a much wider tolerance is also retained when only one of the two pulse strengths is varied.

Action taken: We have added contour lines in Supplementary Fig.10 and 11 to clearly identify the regions with sizable biexciton population and thus to visually assess the robustness against power and frequency fluctuations. Furthermore, we have added a new Supplementary Section IX C: "Influence of pulse-area imbalance", together with the corresponding figure and discussion.

Review comment 1.5: ad Reviewer comment 1.11: I think that the robustness of the scheme under realistic experimental conditions, i.e. the Figure S5 and the corresponding text should be in the main text. The title of the paper is "Robust" and all the presented calculations are backing the robustness with data, but then Figure S5 shows that a very specific detuning has to be used to not run into other excitation regimes such as LA-phonon assisted excitation or excited-state excitation, which is not included in the presented calculations. As this puts the presented scheme into the correct light of how robust it actually is, I believe this paragraph should be not hidden in the supplemental.

Response:

We thank the reviewer for emphasizing the importance of presenting the experimentally accessible detuning range clearly. We agree that this practical constraint should not be apparent only from the Supplementary Information. We have therefore added an explicit discussion in the main text stating that, for the weakly confined GaAs/AlGaAs QD investigated here, the usable detuning range is finite but remains sizable ($\gtrsim 1$ meV). When applied to systems with stricter selection rules and less dense level distribution, such as InAs/GaAs QDs, this range could potentially extend to above $\gtrsim 10$ meV [Phys. Rev. Lett. 95, 177403 (2005)].

We respectfully prefer to retain Fig. S5 in the Supplementary Information. The figure provides the detailed spectroscopic characterization used to identify these additional excitation channels, whereas the central result relevant to assessing the practical applicability

of FTPE—the existence and origin of the finite detuning window—is now stated explicitly in the main text. We would also like to clarify that the robustness emphasized in this work is primarily associated with tolerance to pulse-area fluctuations. The detuning window characterized in Fig. S5 therefore addresses a complementary practical consideration rather than the main robustness metric discussed in the manuscript. Keeping the full spectroscopy figure in the Supplementary Information allows the main text to remain focused on the working principle, robustness, and experimental demonstration of FTPE, while the corresponding experimental evidence remains readily accessible to the reader through the explicit reference to Supplementary Fig. S5.

Action taken: We have added a discussion of the experimentally accessible detuning window to the main text and clarified that the restriction observed here is specific to the present QD system.

> ... In the present weakly confined GaAs/AlGaAs QD, the practical detuning window is finite but remains sizable ($\gtrsim$ 1 meV, Supplementary Section VI). This restriction is specific to the present QD system rather than an intrinsic limitation of FTPE. ...

Review comment 1.6: ad Reviewer comment 1.15: I think my original comment was partly misunderstood: my question was whether there is a point on the FTPE parameters, where the effective AC stark shift cancels out and thus makes the entanglement even better than in TPE? As the authors pointed out in the answer to Reviewer Comment 1.16, maybe this is not the right picture, but then in the answer to comment 1.15, they calculated the entanglement fidelity. Thus, the theory is able to capture the effect of the lasers on the FSS, otherwise this calculation would not be feasible. It seems from the answer to comment 1.15 that FTPE is giving worse results for entanglement fidelity than TPE, weakening the presented paper. The answer to this question is thus quite central to the claims of a more robust and better scheme.

Response: We thank the reviewer for clarifying this point. Without a rotating frame which makes the Hamiltonian (at least approximately) constant, the concept of AC Stark shift is not well defined, and hence this is indeed not the right picture. Our calculations indeed show that the FTPE fidelity is slightly lower (0.2 percentage points) than that of TPE under the conditions considered. This small reduction arises from the longer effective driving duration and larger pulse areas in FTPE, which produce a slightly larger transient spectral shift. Importantly, the demonstrated robustness in this work refers to the BX preparation rather than to an enhancement of the entanglement fidelity over TPE. To avoid any ambiguity on this point, we have revised the paper title accordingly and added a quantitative comparison to the Supplementary Information.

Action taken: The title has been revised to “Robust biexciton preparation for entangled photon-pair generation by single-shot Floquet driving.” In addition, we have added the following

clarification to the Supplementary Information:

> ... The simulated entanglement fidelities achieved by FTPE are close to that of TPE, though a small decrease of a fraction of a percent can be observed when using delayed pulses. Without FSS and the CW laser, the calculated fidelities are 98.2% for TPE, 98.0% for FTPE at $\tau = 0$ ps, and 97.7% for FTPE at $\tau = -2.9$ ps. The slightly lower fidelity of delayed FTPE arises from the longer effective driving duration and larger pulse areas, which lead to a slightly larger pulse-induced transient spectral shift. ...

Review comment 1.7: ad Review comment 1.16: the additional figure R14 is quite interesting. Also another reviewer asked the same question. I think presenting Figure R14 with some description in the SI would be useful.

Response: We thank the reviewer for this suggestion. We agree that the independent scan of the two pulse amplitudes provides useful information about the robustness of FTPE.

Action taken: We have added Supplementary Fig. 11 (formerly R14) and the corresponding discussion to the Supplementary Section IX C. The new section clarifies that equal pulse amplitudes are near optimal but are not a strict requirement for obtaining high BX occupation.

Review comment 1.8: additional comments: - in Table 1 the robustness is classified as strong, whereas it is moderate for SUPER. I do disagree with this assessment after seeing the additional presented data. I would say that the robustness against common intensity fluctuations is maybe a bit better than in SUPER, but the robustness of differential intensity fluctuations looks like it is worse or maybe comparable to SUPER and the robustness of the detuning is actually much worse than in SUPER, as in SUPER both the pulses are red detuned and one can select freely whatever detuning one wants, as there is no problem with LA-phonons or higher excited states. This is a strong limitation of the presented scheme and should be presented as such. If SUPER is classified as moderate than FTPE should also be moderate or even weak-moderate. Maybe the title should also be reconsidered, as the additional presented data shows that the scheme is not as robust as initially claimed.

Response: We thank the reviewer for this careful comparison. Although we also presented robustness against detuning fluctuations/spectral wandering in Supplementary Section IX B, we specifically want to emphasize the robustness against pulse-area variations. We correspondingly changed the category in Table 1 from “Robustness” to “Pulse-area robustness”. As discussed above, Supplementary Fig. 11b clearly shows that FTPE can be classified as “Strong” in this category.

We note that SUPER actually requires a suitable ratio between the two pulse areas for

a chosen detuning. Deviations from this optimal pulse-area ratio cause the population to drop fast below 0.5, showing no plateau feature in the pulse area-detuning map. We calculate and present in Supplementary Fig. 15 (reproduced here as Fig. R1) the biexciton occupation for different pulse areas for SUPER biexciton excitation parameters from Ref. [Phys. Rev. B 107, 035425 (2023)]. The pulse-area regions of high biexciton population enclosed within the corresponding contour lines for SUPER is found to be significantly smaller than the corresponding areas for FTPE with finite delay. Thus, we find it indeed justified to label SUPER as "Moderate" compared to FTPE if the category is specified to "Pulse-area robustness".

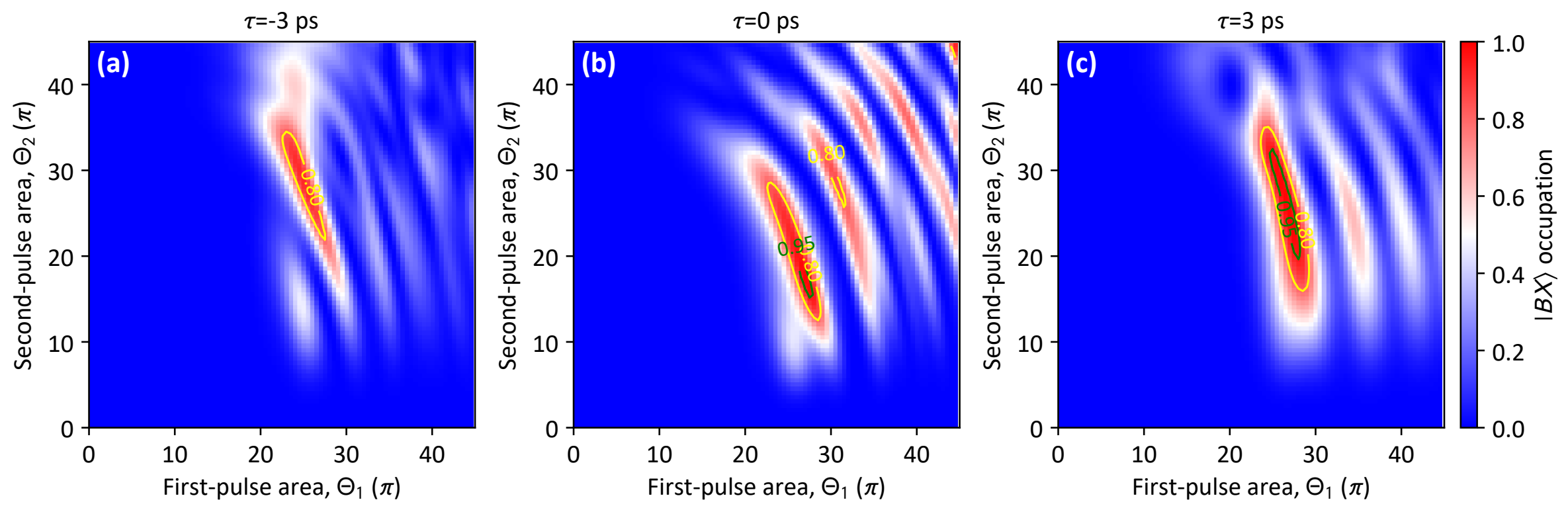

Figure R1: Displayed are the biexciton-occupation after the excitation using the SUPER scheme for different laser amplitudes Θ_1 and Θ_2 as well as delays τ. The yellow and green contour lines denote 80% and 95% excitation, respectively. The excitation parameters are taken from [Phys. Rev. B 107, 035425 (2023)]. The sign of τ is chosen such that $\tau < 0$ describes the red laser arriving early.

Action taken: We have renamed the corresponding column in Table 1 from "Robustness" to "Pulse-area robustness".

We added Supplementary Fig. 15 and described it in the Supplementary Section X D:

> In Ref. [S14] the use of SUPER for the excitation of the biexciton state was investigated. Starting with the same parameters as in Ref. [S14], we vary both pulse areas and depict the resulting biexciton population in Supplementary Fig. 15 for three different pulse delays. Compared to the corresponding results for FTPE, which are displayed in Supplementary Fig. 11b, SUPER is more sensitive to variations in the resonance conditions, as can be seen by the fact that the area enclosed in the 80% and the 95% is much considerably larger for FTPE.

Review comment 1.9: - I noticed that the robustness of the entanglement generation is only claimed and not shown, as only a single point of entanglement is measured. As robust entanglement generation is in the title, the authors could maybe measure at least one second

point in their parameter space to show that the entanglement generation is indeed robust, or alternatively also remove this claim from the title.

Response: We agree that the original title could imply robustness of the entanglement fidelity, whereas the demonstrated robustness refers mainly to biexciton preparation. We have therefore revised the title to make this distinction explicit.

Action taken: The title has been revised to "Robust biexciton preparation for entangled photon-pair generation by single-shot Floquet driving".

Reviewer #3 :

Dear Editors,

I have gone through the revised manuscript and authors responses. I feel the manuscript is suitable for publication and I have no further comments.

Response: We sincerely thank Reviewer #3 for carefully evaluating the revised manuscript and for concluding that it is suitable for publication. We greatly appreciate the reviewer's constructive comments during the first review round, which helped us improve the manuscript.

Reviewer #4 :

The authors have satisfactorily addressed all of the concerns raised during the review process. The manuscript has been significantly improved, and I am pleased to recommend it for publication in Nature Communications.

Response: We sincerely thank Reviewer #4 for the positive evaluation of the revised manuscript and for recommending it for publication. We greatly appreciate the reviewer's constructive comments during the first review round, which helped us improve the manuscript.
